# Meta-analysis of genome-wide association studies identifies ancestry-specific associations underlying circulating total tau levels

**DOI:** 10.1038/s42003-022-03287-y

**Published:** 2022-04-08

**Authors:** Chloé Sarnowski, Mohsen Ghanbari, Joshua C. Bis, Mark Logue, Myriam Fornage, Aniket Mishra, Shahzad Ahmad, Alexa S. Beiser, Eric Boerwinkle, Vincent Bouteloup, Vincent Chouraki, L Adrienne Cupples, Vincent Damotte, Charles S. DeCarli, Anita L. DeStefano, Luc Djoussé, Alison E. Fohner, Carol E. Franz, Tiffany F. Kautz, Jean-Charles Lambert, Michael J. Lyons, Thomas H. Mosley, Kenneth J. Mukamal, Matthew P. Pase, Eliana C. Portilla Fernandez, Robert A. Rissman, Claudia L. Satizabal, Ramachandran S. Vasan, Amber Yaqub, Stephanie Debette, Carole Dufouil, Lenore J. Launer, William S. Kremen, William T. Longstreth, M Arfan Ikram, Sudha Seshadri

**Affiliations:** 1 Department of Epidemiology, Human Genetics, and Environmental Sciences, University of Texas Health Science Center at Houston, Houston, TX USA; 2 Department of Epidemiology, Erasmus University Medical Center, Rotterdam, The Netherlands; 3 Department of Genetics, School of Medicine, Mashhad University of Medical Sciences, Mashhad, Iran; 4 Cardiovascular Health Research Unit, Department of Medicine, University of Washington, Seattle, WA USA; 5 National Center for PTSD, Behavioral Sciences Division, VA Boston Healthcare System, Boston, MA USA; 6 Department of Psychiatry and Biomedical Genetics, Boston University School of Medicine, Boston, MA USA; 7 University of Texas Health Sciences Center at Houston, Houston, TX USA; 8 University of Bordeaux, Inserm, Bordeaux Population Health Research Center, team VINTAGE, UMR 1219, F-33000 Bordeaux, France; 9 Division of Systems Biomedicine and Pharmacology, Leiden Academic Centre for Drug Research, Leiden University, Leiden, The Netherlands; 10 Department of Biostatistics, Boston University School of Public Health, Boston, MA USA; 11 Boston University and the NHLBI’s Framingham Heart Study, Boston, MA USA; 12 Department of Neurology, Boston University School of Medicine, Boston, MA USA; 13 Centre Inserm U1219 Bordeaux Population Health, CIC1401-EC, Institut de Santé Publique, d’Epidémiologie et de Développement, Université de Bordeaux, CHU de Bordeaux, Pôle Santé Publique, Bordeaux, France; 14 Univ. Lille, Inserm, CHU Lille, Institut Pasteur de Lille, U1167 - RID-AGE- LabEx DISTALZ - Risk factors and molecular determinants of aging diseases, F-59000 Lille, France; 15 Department of Neurology and Center for Neuroscience, University of California at Davis, Davis, CA USA; 16 Department of Medicine, Division of Aging, Brigham and Women’s Hospital and Harvard Medical School, Boston, MA USA; 17 Institute of Public Health Genetics and Department of Epidemiology and Cardiovascular Health Research Unit, University of Washington, Seattle, WA USA; 18 Department of Psychiatry and Center for Behavior Genetics of Aging, University of California, San Diego, La Jolla, CA USA; 19 Glenn Biggs Institute for Alzheimer’s & Neurodegenerative Diseases, University of Texas Health Sciences Center, San Antonio, TX USA; 20 Department of Psychology and Brain Sciences, Boston University, Boston, MA USA; 21 University of Mississippi Medical Center, Jackson, MS USA; 22 Beth Israel Deaconess Medical Center, Harvard Medical School, Boston, MA USA; 23 Turner Institute for Brain and Mental Health, Monash University, Melbourne, VIC Australia; 24 Harvard T.H. Chan School of Public Health, Harvard University, Boston, MA USA; 25 Department of Neurosciences, University of California, San Diego, La Jolla, CA USA; 26 Preventive Medicine & Epidemiology, Boston University School of Medicine, Boston, MA USA; 27 Bordeaux University Hospital, Department of Neurology, Institute for Neurodegenerative Diseases, Bordeaux, France; 28 National Institute on Aging, Baltimore, MD USA; 29 Departments of Neurology and Epidemiology, University of Washington, Seattle, WA USA

**Keywords:** Genome-wide association studies, Genetic variation

## Abstract

Circulating total-tau levels can be used as an endophenotype to identify genetic risk factors for tauopathies and related neurological disorders. Here, we confirmed and better characterized the association of the 17q21 *MAPT* locus with circulating total-tau in 14,721 European participants and identified three novel loci in 953 African American participants (4q31, 5p13, and 6q25) at *P* < 5 × 10^−8^. We additionally detected 14 novel loci at *P* < 5 × 10^−7^, specific to either Europeans or African Americans. Using whole-exome sequence data in 2,279 European participants, we identified ten genes associated with circulating total-tau when aggregating rare variants. Our genetic study sheds light on genes reported to be associated with neurological diseases including stroke, Alzheimer’s, and Parkinson’s (*F*5, *MAP1B*, and *BCAS3*), with Alzheimer’s pathological hallmarks (*ADAMTS12*, *IL15*, and *FHIT*), or with an important function in the brain (*PARD3*, *ELFN2*, *UBASH3B*, *SLIT3*, and *NSD3*), and suggests that the genetic architecture of circulating total-tau may differ according to ancestry.

## Abstract (web summary)

A meta-analysis of genome-wide association studies of circulating total-tau levels identifies loci specific to European or African American ancestries, providing further insight to the genetic etiology of neurological diseases.

## Introduction

The protein tau is an important biomarker of neuronal injury and neurodegeneration. Alzheimer’s disease (AD) and other dementias or related neurological disorders are associated with abnormal intraneuronal tau aggregates (collectively known as tauopathies)^1^. Newer techniques to diagnose AD now examine CSF biomarkers to improve diagnostic certainty and aid in earlier diagnosis^2–4^. However, their collection is invasive and user variability can be large in the downstream quantification assays. In addition, CSF tau levels are normal or low in tauopathies like Progressive Supranuclear Palsy (PSP) and in frontotemporal dementia patients with tau mutations^5,6^.

Using blood biomarkers with high specificity and sensitivity for AD is ideal to lower cost, risk, and burden^3^. Circulating total-tau (t-tau) levels can be quantified in serum or in plasma^7^ early in AD due to blood brain barrier breakdown^8,9^. Particularly, they show promise as a predictive biomarker for dementia and related endophenotypes^10^, with higher levels in patients with dementia or mild cognitive impairment compared to controls^11,12^, and higher levels associated with poorer cognitive performance, and smaller hippocampal volumes^13,14^. However, elevated levels may lack diagnostic specificity for AD, and simply indicate that brain injury is common to several neurological diseases. A recent paper showed for example that higher circulating t-tau predicted a higher risk of incident stroke^15^. Importantly, circulating biomarkers do not need to mirror their level in CSF to be useful. Altogether, the recent literature suggests that circulating t-tau levels may be a predictive biomarker to improve risk stratification for dementia and assess AD’s progression, help with enrollment of high-risk individuals into dementia prevention trials, be useful in addition to other blood biomarkers of neurodegeneration to determine cognitive improvements in clinical trials, and represent a useful biomarker for AD when added to CSF tau measures^10,15–17^.

CSF t-tau and phosphorylated tau (p-tau) levels have been used as endophenotypes in genome-wide association studies (GWAS) to detect genetic variants associated with AD risk. Similarly, circulating t-tau levels may be used as an endophenotype to identify genetic risk factors for tauopathies and related neurological disorders. Only two GWAS, conducted in the Alzheimer’s Disease Neuroimaging Initiative (ADNI) study, were published for plasma t-tau or p-tau levels^18,19^. The modest sample size and the inclusion of only European participants has limited the statistical power to identify potential novel associations (only *MAPT* and *APOE* loci associations were statistically significant for t-tau and p-tau respectively), the ability to explore less frequent genetic variation, as well as the generalization of the findings to other ancestries. Therefore, the aim of our study was to perform large-scale meta-analyses of circulating t-tau levels, using 15,674 participants from eight studies representing two ancestries (Europeans and African Americans), to explore genetic variation underlying circulating t-tau levels and assess their overlap with known genetic determinants of neurological diseases. We detected four ancestry-specific loci at the genome-wide significance (17q21 in Europeans, and 4q31, 5p13, and 6q25 in African Americans). We identified pleiotropic associations at 17q21 and 1q24 which, combined with the detection of an enrichment of genes associated with neurological diseases or related traits, suggested that a potential overlap exists between genetic determinants of circulating t-tau levels and several neurological disorders and traits including AD and stroke.

## Results

### Populations and participants

We included in our meta-analyses 15,674 participants from eight studies representing two major ancestries: Europeans (*N* = 14,721) and African Americans (*N* = 953) (Table 1, 2). A description of each study is included in the Supplementary Notes 1–8. Proportion of males varied from 35% (The Cardiovascular Health Study, African Americans) to 100% (The Vietnam Era Twin Study of Aging). Mean age ranged from 49 years (3.5) for The Coronary Artery Risk Development in Young Adults, African Americans to 78 years (4.3) for The Cardiovascular Health Study, African Americans (Table 2).

**Table 1:** Description of the European-ancestry participants included in the meta-analysis of circulating total-tau levels.

	FHS (*N* = 6018)	RSI (*N* = 2169)	RSII (*N* = 960)	MEM1 (*N* = 336)	MEM2 (*N* = 1738)	CARDIA (*N* = 315)	CHS (*N* = 1396)	ARIC (*N* = 549)	VETSA (*N* = 754)	ADNI (*N* = 486)
Men, *N* (%)	2790 (46)	1696 (59)	951 (44)	153 (45)	666 (37)	149 (47)	523 (37)	257 (47)	754 (100)	292 (60)
Age, mean (SD)	56.81 (13.98)	75.30 (6.10)	67.80 (7.10)	67.60 (8.58)	71.47 (8.56)	51.02 (3.25)	77.91 (4.3)	63.52 (4.38)	67.50 (2.51)	75.44 (6.71)
Age, median [25–75%]	56.1 [46.4–66.3]	74.4 [70.5–79.4]	65.5 [62.8–70.8]	68.0 [62.9–73.6]	72.2 [66.1–77.6]	52.0 [49.0–53.5]	77.0 [75.0–80.3]	64.0 [60.0–67.0]	68.1 [65.4–69.7]	75.8 [71.5–80.1]
Circulating t-tau, mean (SD)	4.13 (2.12)	2.60 (0.90)	2.60 (3.10)	2.10 (0.88)	2.23 (1.84)	0.54 (1.26)	0.46 (2.18)	0.41 (0.54)	2.02 (1.23)	2.88 (1.64)
Circulating t-tau, median [25–75%]	3.91 [3.24–4.73]	2.50 [1.90–3.0]	2.40 [1.90–2.90]	2.01 [1.55–2.60]	1.86 [1.41–2.60]	0.34 [0.20–0.57]	0.27 [0.17–0.43]	0.18 [0.09–0.34]	1.76 [1.29–2.32]	2.71 [1.93–3.45]
Circulating t-tau (log), mean (SD)	1.97 (0.46)	1.30 (0.50)	1.20 (0.50)	0.91 (0.81)	0.95 (0.73)	−1.54 (1.14)	−1.31 (0.83)	−1.68 (0.96)	0.82 (0.72)	0.92 (0.57)
Circulating t-tau (log), median [25–75%]	1.97 [1.70–2.24]	1.30 [0.90–1.60]	1.20 [0.90–1.60]	1.01 [0.63–1.38]	0.90 [0.50–1.38]	−1.56 [−2.32, −0.81]	−1.30 [−1.75, −0.85]	−1.71 [−2.34, −1.10]	0.81 [0.37–1.21]	1.00 [0.66–1.24]

*FHS* Framingham Heart Study, *RSI and RSII* The Rotterdam Study, *MEM1 and MEM2* The MEMENTO Study, *CARDIA* The Coronary Artery Risk Development in Young Adults Study, *CHS* The Cardiovascular Health Study, *VETSA* The Vietnam Era Twin Study of Aging Study, *ARIC* The Atherosclerosis Risk in Communities Study, *ADNI* The Alzheimer’s Disease Neuroimaging Initiative Study.

**Table 2:** Description of the African American participants included in the meta-analysis of circulating total-tau levels.

	CARDIA (*N* = 111)	CHS (*N* = 273)	ARIC (*N* = 569)
Men, *N* (%)	51 (46)	96 (35)	218 (38)
Age, mean (SD)	48.78 (3.46)	76.32 (4.93)	61.68 (4.47)
Age, median [25–75%]	48.0 [46.0–51.0]	75.0 [72.0–80.0]	61.0 [58.0–65.0]
Circulating t-tau, mean (SD)	0.57 (1.41)	0.46 (0.94)	0.50 (0.77)
Circulating t-tau, median [25–75%]	0.37 [0.22–0.60]	0.28 [0.18–0.46]	0.31 [0.20–0.50]
Circulating t-tau (log), mean (SD)	−1.54 (1.03)	−1.24 (0.85)	−1.57 (1.10)
Circulating t-tau (log), median [25–75%]	−1.43 [−2.24, −0.78]	−1.27 [−1.73, −0.78]	−1.69 [−2.36, −0.99]

*CARDIA* The Coronary Artery Risk Development in Young Adults Study, *CHS* The Cardiovascular Health Study, *ARIC* The Atherosclerosis Risk in Communities Study.

### Main meta-analysis results

**Fig. 1:**
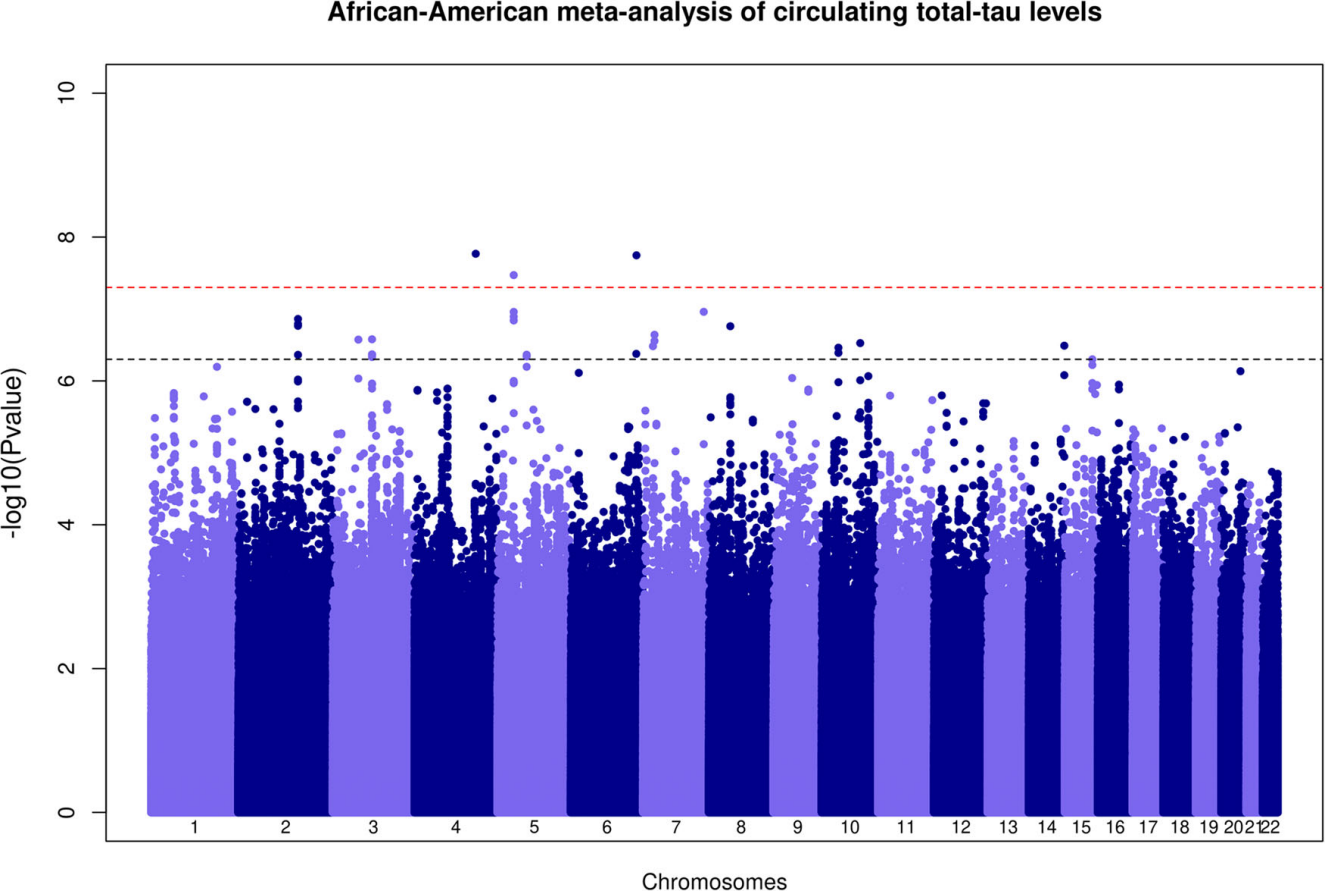
Manhattan plot of association *P* values for the African American specific meta-analysis of GWAS of circulating total-tau levels. The –log10(*P*)-value for each single nucleotide variant on the *y* axis is plotted against the build 37 genomic position on the *x* axis (chromosomal coordinate). The dashed horizontal red line indicates the genome-wide significance threshold of *P* = 5 × 10^−8^ and the dashed horizontal black line indicates the threshold of *P* = 5 × 10^−7^.

**Fig. 2:**
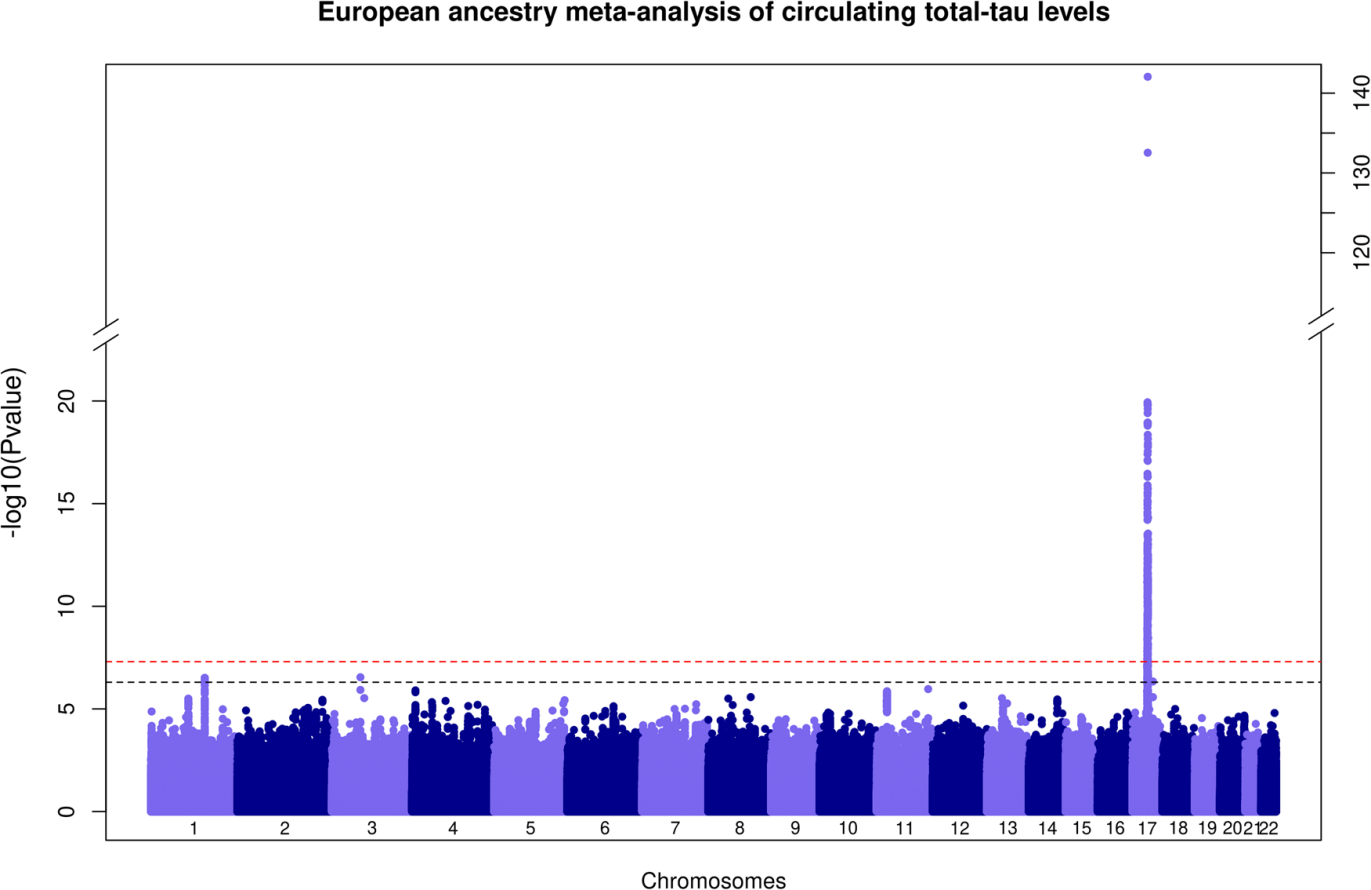
Manhattan plot of association *P* values for the European ancestry specific meta-analysis of GWAS of circulating total-tau levels. The –log10(*P*)-value for each single nucleotide variant on the *y* axis is plotted against the build 37 genomic position on the *x* axis (chromosomal coordinate). The dashed horizontal red line indicates the genome-wide significance threshold of *P* = 5 × 10^−8^ and the dashed horizontal black line indicates the threshold of *P* = 5 × 10^−7^. The *y* axis was truncated for ease of interpretation.

**Fig. 3:**
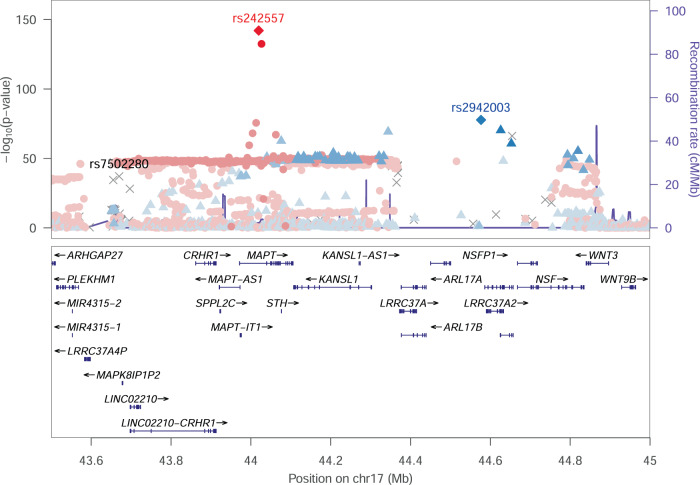
Regional association plot at the *MAPT* 17q21 locus. Genetic variants are plotted with their *P* values (-log10 values, left *y* axis) as a function of the build 37 genomic position (*x* axis). Estimated recombination rates (right *y* axis) reflect the local linkage disequilibrium (LD) structure around the top distinct associated genetic variants (red and blue diamonds, denoting rs242557 and rs2942003, respectively), identified using stepwise model selection procedure, and their correlated proxies. Genetic variants in LD with rs242557 are indicated with circles according to a light to dark red scale, from r² = 0 to 1. Genetic variants in LD with rs2942003 are indicated with triangles according to a light to dark blue scale, from r² = 0 to 1. Gray crosses (X) represent SNPs with missing LD. The third distinct genetic variant (rs7502280), indicated in black on the plot, was not present in the reference panel to calculate the LD with the other variants in the region. Reference panel used was the 1000 Genomes November 2014 European population.

Quantile–Quantile and Manhattan plots for each ancestry-specific meta-analysis of circulating t-tau levels are presented in Figs. 1, 2 and Supplementary Fig. 1. We confirmed and better characterized the strong association of genetic variants at the microtubule associated protein tau (*MAPT*) 17q21 locus in European participants (lead genetic variant rs242557-A, effect allele frequency = 0.38, beta = 0.20, *P* = 8.9 × 10^−143^, proportion of variance explained (PVE) ~4.2%, Table 3). Stepwise model selection procedure at the 17q21 locus identified three distinct signals (rs242557-A, beta = 0.20, *P* = 2.3 × 10^−143^; rs7502280-T, beta = 0.17, *P* = 5.7 × 10^−38^, PVE ~ 1.1%, and rs2942003-T, beta = 0.16, *P* = 3.9 × 10^−78^, PVE ~ 2.3%), Table 3, Supplementary Table 1 and Fig. 3. We also detected, at the genome-wide threshold (*P* < 5 × 10^−8^), three potential novel loci in African American participants at 4q31 (lead genetic variant rs111836296-T, effect allele frequency = 0.06, beta = −0.54, *P* = 1.7 × 10^−8^), 5p13 (rs74710969-T, effect allele frequency = 0.11, beta = −0.53, *P* = 3.4 × 10^−8^, PVE ~ 3%) and 6q25 (rs674432-C, effect allele frequency = 0.97, beta = 0.68, *P* = 1.8 × 10^−8^, Table 3). The variants rs111836296 and rs74710969 are extremely rare or monomorphic in European populations. We detected additional associations at *P* < 5 × 10^−7^ at eleven loci (2q23, 3p14, 3q11, 5q13, 7p21, 7p15, 7q36, 8p11, 10p11, 10q23, and 14q32) in African American participants and three loci (1q24, 3p14, and 17q23) in European participants (Supplementary Tables 2, 3). All the identified hits were ancestry specific (absence of association or monomorphic variant in one ancestry) as indicated by the high heterogeneity (Table 3 and Supplementary Table 3). Forest plots are presented in Supplementary Figs. 2–11 and indicated consistent direction of effects across studies. Regional association plots for all loci are presented in Supplementary Figs. 12–27.

**Table 3:** Lead genetic variants passing the genome-wide significance threshold (*P* < 5 × 10^−8^) in the meta-analysis of GWAS of circulating total-tau levels in European-ancestry participants (*N* = 14,721) or African American participants (*N* = 953).

			Europeans (*N* = 14,721)						African Americans (*N* = 953)						Multi-ancestry				
rsid	Chr:b37Pos	Alleles	EAF	Beta	SE	*P*	I^2^	P_Q_	EAF	Beta	SE	*P*	I^2^	P_Q_	*P*_RE2_	I^2^	P_Q_		Gene
rs111836296*	4:142551581	T/C	--	--	--	--	--	--	0.06	−0.54	0.10	**1.7E-08**	0	0.37	--	--	--		intergenic
rs74710969*	5:33618142	T/G	--	--	--	--	--	--	0.11	−0.53	0.10	**3.4E-08**	0	0.88	--	--	--		*ADAMTS12*
rs674432	6:158101153	C/G	0.83	−0.001	0.01	0.90	0	0.45	0.97	0.68	0.12	**1.8E-08**	0	0.32	7.6E-06	96.84	**1.8E-08**		intergenic
rs7502280*	17:43670221	T/G	0.88	0.17	0.01	**7.8E-38**	0	0.53	--	--	--	--	--	--	--	--	--		intergenic
rs242557	17:44019712	A/G	0.38	0.20	0.01	**8.9E-143**	42.5	0.07	0.28	0.05	0.07	0.50	76.2	0.04	**8.5E-142**	76.46	0.04		*MAPT*
rs2942003	17:44576704	T/G	0.34	0.16	0.01	**1.9E-78**	44.1	0.07	0.66	0.09	0.13	0.51	0	1.00	**1.5E-77**	0.00	0.57		intergenic

*EAF* effect allele frequency, Alleles: effect (Alternate) allele/non-effect (Reference) allele.

I^2^: I-square heterogeneity statistic; P_Q_: Cochran’s Q statistic’s *P* value.

*P* values in bold pass the genome-wide significance threshold of *P* < 5 × 10^−8^.

*rs111836296 on chr4 and rs74710969 on chr5 are extremely rare in Europeans and thus they were not included in the multi-ancestry meta-analysis; rs7502280 is not present in the 1000 Genomes reference panel and thus was not present in the African American GWAS results.

*P*_RE2_: Han and Eskin’s random effects model to detect associations under heterogeneity^59^.

We did not observe an association of the two SNPs defining *APOE* in the main circulating t-tau European meta-analysis, consistent with previous finding.^18^ In the additional *APOE4*-stratified analyses, we observed similar magnitude and consistent direction of effects for the main associations identified in Europeans (Table 4).

**Table 4:** Results of the *APOE4*-stratified analyses for the lead genetic variants in each locus passing the threshold of *P* < 5 × 10^−7^ in the European meta-analysis of GWAS of circulating total-tau levels.

						Main analysis (*N* = 14,721)		*APOE4* carriers (*N* = 3640)		*APOE4* Non-carriers (*N* = 10,574)		
rsid	Chr	Build 37 Pos (bp)	Eff	NEff	EAF	Beta	*P*	Beta	*P*	Beta	*P*	Gene
rs6686805	1	169,512,643	A	C	0.67	−0.04	3.4E-07	−0.02	0.21	−0.04	5.1E-07	*F5*
rs139843727	3	66,316,022	A	C	0.99	0.22	2.9E-07	0.08	0.44	0.24	6.5E-07	*SLC25A26*
rs7502280	17	43,670,221	T	G	0.88	0.17	8.0E-38	0.16	2.2E-09	0.18	1.4E-30	intergenic
rs242557	17	44,019,712	A	G	0.38	0.20	8.9E-143	0.19	1.3E-37	0.20	1.4E-105	*MAPT*
rs2942003	17	44,576,704	T	G	0.34	0.16	1.9E-78	0.17	3.5E-22	0.16	3.4E-58	intergenic
rs4968553	17	59,428,962	C	G	0.16	−0.05	4.6E-07	−0.05	0.02	−0.06	1.7E-07	*BCAS3*

*EAF* effect allele frequency, *Eff* effect (alternate) allele, *Neff* non-effect (reference) allele.

The association of the two SNPs defining *APOE* in the main European meta-analysis were: rs429358-T (Beta = −0.02, *P* = 0.10) and rs7412-T (Beta = 0.01, *P* = 0.49).

### Overlap of circulating t-tau genetic determinants with neurological diseases and traits

In addition to the strong associations at the *MAPT* locus that is known to be pleiotropic, we identified association at 1q24, a locus previously reported for stroke, Supplementary Table 4.^20^ The lead genetic variant in our analysis (rs6686805) was in linkage disequilibrium with rs1800594, a GWAS hit for ischemic stroke. Analyses conducted with FUMA, based on the main results from the European circulating t-tau meta-analysis, identified significantly differentially expressed genes in brain cerebellar hemisphere and brain cerebellum (Supplementary Fig. 28) and enrichment of genes in gene sets reported by GWAS of neurological diseases or traits including Parkinson Disease (PD), craniofacial microsomia, intracranial volume, cognitive function, subcortical brain region volumes, and AD in APOE E4- carriers, as well as risk factors such as body mass index (Supplementary Fig. 29). Finally, a genetic risk score (GRS) based on the distinct genome-wide associations (two *MAPT* genetic variants, rs242557 and rs376284405) from our European meta-analysis of circulating t-tau levels (excluding the Framingham Heart Study, FHS) was strongly associated with circulating t-tau levels (beta = 0.3, *P* = 4 × 10^−97^, PVE = 7%) and was associated with intracranial volume (beta = 15.1, *P* = 3 × 10^−4^) in FHS. We did not detect significant associations with the other traits tested (Supplementary Table 5). Altogether, these findings suggest an overlap of the genetic associations of circulating t-tau levels with known genetic determinants of neurological disorders and associated traits.

### Two sample Mendelian Randomization (MR) analyses

Using two sample MR and large GWAS summary statistics, we did not identify significant causal associations between circulating t-tau levels and AD, PD, stroke, or White Matter Hyperintensities (WMH) (Supplementary Table 6). We also tested the opposite hypothesis and did not find significant causal associations (Supplementary Table 7). Our results did not indicate significant heterogeneity or presence of directional horizontal pleiotropy, except for a few analyses (WMH or PD as exposure; stroke as outcome). We also performed power calculations for the MR where circulating t-tau levels was the exposure variable (Supplementary Table 8) that indicated that our analysis would be underpowered if the instruments had small effects on the neurological outcomes (especially for AD and PD analyses with smaller numbers of cases).

### Rare variant analyses using whole-exome sequence data

Using SKAT (variance component test) or CMC (burden test), our rare variant aggregation tests based on whole-exome sequence data identified 10 genes (*ELFN2*, *UBASH3B*, *RUSF1*, *ZFP28*, *LCT*, *REM1*, *DELE1*, *SLIT3*, *NSD3*, and *MYO1G*) significantly (*P* ≤ 1.25 × 10^−6^) associated with circulating t-tau levels when aggregating rare variants (MAF ≤ 5% or MAF ≤ 1%) with high or moderate impact, including some missense and loss of function variants (Supplementary Tables 9, 10 and Supplementary Figs. 30, 31). All except one gene (*MYO1G*) were detected with SKAT at the gene level significance threshold (*P* = 1.25 × 10^−6^), while at least nominally significant associations were observed for most genes with CMC (Supplementary Tables 9, 10). These results indicate that rare variants in those genes were likely to have different magnitudes and directions of effects, including no effect. This is a likely scenario as the number of rare variants aggregated for each gene was somewhat large, ranging from 13 to 64 variants. Similar results were observed for the two sets of annotations tested (missense or loss of function versus high or moderate impact). This observation, combined with the fact that one set of annotations is a subset of the other, and the number of genetic variants contributing to both analyses did not differ drastically, suggested that the same variants were selected to be aggregated in both analyses. Similar results were also observed for the two MAF thresholds tested, except for *NSD3* and *SLIT3*. For these two genes, the addition of a small number of more frequent variants (1% < MAF ≤ 5%) attenuated the association.

## Discussion

The goal of our study was to characterize genetic variation underlying circulating t-tau levels and to explore their overlap with known genetic determinants of neurological diseases. By performing large-scale meta-analyses in more than 15,000 participants from two major ancestries, we identified new genetic variants and genes associated with circulating t-tau levels, all associations being observed in only one ancestry (African Americans or Europeans). We identified pleiotropic signals at two regions (17q21 and 1q24) that were previously reported for plasma t-tau, AD, PD, WMH, and PSP (*MAPT*) or stroke (*F5*), respectively, and enrichment of genes associated with neurological diseases or related traits. Thus, our analyses highlighted that an overlap may exist between genetic determinants of circulating t-tau levels and several neurological disorders and traits including AD.

Our findings confirmed the importance of genes and pathways already well known to be involved in AD or other tauopathies and neurological diseases. Indeed, we first confirmed the strong association in Europeans of the 17q21 *MAPT* locus (lead genetic variant rs242557), which has been reported to be associated with circulating t-tau levels^18^. The *MAPT* locus has also been associated with AD, PD, and PSP, Supplementary Table 4^20–22^, indicating an important role of *MAPT* in many neurodegenerative diseases. This locus has also been associated with head size^23–25^ and notably child head circumference^23^, which may indicate possible effects of this inversion on brain development very early in life. We found an enrichment for gene sets reported associated with craniofacial microsomia, intracranial volume, and subcortical brain region volumes, which tend to also support this hypothesis (Supplementary Fig. 29). We also identified a significant positive association of a GRS, constructed based on two distinct *MAPT* genetic variants (rs242557 and rs376284405), with intracranial volume in the FHS, while these variants were distinct from the ones reported by GWAS of intracranial volume at this locus (rs199525, rs8072451, and rs17689882). Despite PD is not a tauopathy, PSP and corticobasal degeneration, two PD subtypes known as Parkinson-plus syndromes, are both associated with the formation of tau deposits^26^. Here we were also able to identify two additional and distinct signals at 17q21 (rs7502280 and rs2942003). The variant rs7502280 is located at 29 kb of the corticotropin releasing hormone receptor 1 (*CRHR1*) and at 7.3 kb of the mitogen-activated protein kinase 8 interacting protein 1 (*LOC644172*), and is a GWAS hit for relative carbohydrate intake^27^ and sleep duration^28^. The variant rs2942003 lies at 12 kb and tags the leucine rich repeat containing 37 member A2 (*LRRC37A2*) gene (Fig. 3). One 17q21 variant (rs439945) reported by the Parkinson Disease GWAS Consortium was found to be significantly associated with nearby gene expression probes targeting *LRRC37A* and *LRRC37A2* by a study investigating the modification of gene expression in prefrontal cortex brain samples of pathologically confirmed PD cases and controls^29^. Thus, genetic variations in both *MAPT* and *LRRC37A2* appear to be important determinants of tauopathies and neurodegenerative disorders. More research needs to be performed to understand more precisely the mechanisms underlying their contributions to other tauopathies. Particularly, the 17q21 region, a common inversion polymorphism, is complex and may affect the expression of other genes in the region that may also be involved in neurodegenerative disease pathology, possibly in a tissue-specific manner.

In addition to the *MAPT* 17q21 locus identified in Europeans, we detected three potential novel loci in African American participants (4q31, 5p13, and 6q25) at the genome-wide significance level. Two of the lead genetic variants (rs111836296 and rs74710969) were extremely rare in Europeans and lie in or tag candidate genes (*IL15* and *ADAMTS12*) linked to AD and other neurological disorders. The genetic variant rs111836296 at 4q31 lies at 6 kb and tags the interleukin 15 (*IL15*) gene (Supplementary Fig. 15). Serum IL15, a pro-inflammatory cytokine, has been studied as a possible marker of AD^30^. The genetic variant rs74710969 at 5p13 lies in an intron of the ADAM metallopeptidase with thrombospondin type 1 motif 12 (*ADAMTS12)* gene (Supplementary Fig. 16). Previous studies have associated ADAMTSs family of secreted metalloproteases with the repair of the central nervous system, through its ability to degrade neurocan, a novel component of brain extra-cellular matrix. Alterations in this degradation processes could be associated with the pathogenesis of neurological disorders^31^. Several studies also suggest a role for *ADAMTS12* in stroke^32,33^.

We also detected 14 loci at *P* < 5 × 10^−7^, eleven loci in African American participants and three loci in Europeans.

Three signals identified in African Americans (3p14 and 5q13) or Europeans (17q23) lie in genes related to the tubulin-microtubule system (*FHIT*, *MAP1B*, and *BCAS3*). The signal at 3p14 lies in the fragile histidine triad diadenosine triphosphatase (*FHIT*) gene (Supplementary Fig. 13) and the encoded protein interacts with tubulin. The signal at 5q13 lies in the microtubule associated protein 1B (*MAP1B*) gene (Supplementary Fig. 17). Proteins of this family may be involved in microtubule assembly, which is an essential step in neurogenesis. Gene knockout studies of the mouse *MAP1B* gene suggested an important role in development and function of the nervous system. Several studies are also in favor of a role of *MAP1B* in AD^34,35^. MAP1B is also a component of cortical Lewy bodies and binds alpha-synuclein filaments, which suggests that it may be involved in the pathogenesis of Lewy bodies^36^. The signal at 17q23 lies in the BCAS3 microtubule associated cell migration factor (*BCAS3*) gene (Supplementary Fig. 27) that is highly expressed in the brain (GTEx). In mice, Rudhira, a murine WD40 domain protein that is 98% identical to *BCAS3*, has been shown to bind to microtubules and vimentin intermediate filaments to promote cell migration for angiogenic remodeling^37^.

Furthermore, two signals (1q24 and 10p11) lie in genes reported to be associated with AD or stroke or have an important function in the brain (*F5*, and *PARD3*). The signal at 1q24 identified in Europeans lies in the coagulation factor 5 (*F5)* gene (Supplementary Fig. 24). The lead genetic variant rs6686805 is in linkage disequilibrium with rs1800594, a GWAS hit for blood protein levels and ischemic stroke (Supplementary Tables 4, 11),^38–40^ and with rs6030, a missense variant in *F5*. A rare protective variant in *F5* (rs2027885) has been reported to be associated with AD in African Americans^41^ and with hippocampal atrophy^42^. The signal at 10p11 identified in African Americans lies in the par-3 family cell polarity regulator (*PARD3*) gene (Supplementary Fig. 21) that is required for establishment of neuronal polarity and normal axon formation in cultured hippocampal neurons^43,44^. Par3 regulates microtubule stability and organization, crucial for neuronal polarization^45^. Moreover, atypical protein kinase C (aPKC) in complex with PAR-3/PAR-6 negatively regulates microtubule affinity-regulating kinases, which in turn causes dephosphorylation of microtubule-associated proteins, such as tau, leading to the assembly of microtubules and elongation of axons^46^. Par3 also regulates APP processing and trafficking^47^, polarized convergence between APP and BACE1 in hippocampal neurons^48^, and retrograde endosome-to- trans-Golgi network trafficking of BACE1 along with aPKC^49^. Brain regulatory marks (promoter and enhancer) are reported at the lead variant rs12245909 (HaploReg v4.1), which may suggest a functional role of this variant in the brain.

We looked up the main distinct lead genetic variants from the published ADNI GWAS of circulating tau levels^18^ in our European meta-analysis (excluding ADNI). We confirmed the strong association of the *MAPT* rs242557-A genetic variant (Table 5 and Supplementary Table 12). However, we did not find evidence of association for the three other loci that were detected at *P* < 10^−5^ in the original ADNI GWAS, suggesting that these signals may have been false positives.

**Table 5:** Look-up of the main hits (*P* < 10^−5^) from the published ADNI GWAS of circulating tau levels (Chen et al., 2017) in our European meta-analysis of GWAS of circulating total-tau levels (seven studies excluding ADNI).

						Main analysis (*N* = 14,235)		*APOE4* carriers (*N* = 3401)		*APOE4* Non-carriers (*N* = 10,327)		
rsid	Chr	Build 37 Pos (bp)	Eff	NEff	EAF	Beta	*P*	Beta	*P*	Beta	*P*	Gene
rs2187213	6	162,634,337	A	G	0.35	0.0005	0.95	0.005	0.77	−0.0006	0.95	*PARK2*
rs7047280	9	23,297,808	T	C	0.60	−0.0009	0.90	−0.008	0.56	0.003	0.71	*ELAVL2*
rs7072793	10	6,106,266	T	C	0.59	0.008	0.22	0.02	0.17	0.002	0.82	*IL2RA*
rs242557	17	44,019,712	A	G	0.36	0.20	6.4E-136	0.19	3.0E-35	0.20	1.4E-101	*MAPT*

*EAF* effect allele frequency, *Eff* effect (alternate) allele, *Neff* non-effect (reference) allele.

Among the 10 genes identified when leveraging whole exome sequence data and aggregating rare variants with high or moderate impact, four have a function relevant to the brain (*ELFN2*, *UBASH3B*, *SLIT3*, and *NSD3*). Interestingly*, SLIT3* and *NSD3* associations were more impacted by the choice of the MAF threshold to select rare variants to aggregate. For both genes, results were only significant with SKAT when using a MAF ≤ 1%, indicating that only rarer variations were contributing to the associations. The extracellular leucine rich repeat and fibronectin type III domain containing 2 gene (*ELFN2*), is overexpressed in the brain. The encoded protein is a postsynaptic adhesion molecule that selectively binds with group III metabotropic glutamate receptors^50,51^. ELFN1, a protein of the same family, has been reported to be associated with neuropsychiatric disorders (attention deficit hyperactivity disorder, post-traumatic stress disorder, and epilepsy). Distinct neuronal expression patterns are reported for *ELFN1* and *ELFN2*^51^. The ubiquitin associated and SH3 domain containing B gene (*UBASH3B*) is overexpressed in the brain. The encoded protein is a phosphatase, and the concerted action of protein kinases and phosphatases represents a critical signaling event controlling synaptic functions and higher-order brain functions, such as learning and memory^52^. The slit guidance ligand 3 gene (*SLIT3*) encodes an axon guidance molecule expressed by motor neurons^53,54^. *SLIT3* may also play a role in essential tremor disease pathogenesis^55^. The nuclear receptor binding SET domain protein 3 gene (*NSD3*) is highly expressed in the brain. The encoded protein is a SET domain-containing methyltransferase, an epigenetic regulator that is selectively expressed in primary microglia^56^. Follow-up studies are needed to characterize the potential role of these four genes in tauopathies.

A summary of the neurological traits reported in the GWAS catalog for genetic variants in the main genes identified in the meta-analyses of circulating t-tau levels (*IL15*, *FHIT*, *ADAMTS12*, *PARD3*, *F5*, *BCAS3*, *UBASH3B*, and *SLIT3*) and described above is available in Supplementary Table 11.

By performing meta-analyses separately in African Americans and European-ancestry participants, we were able to identify ancestry-specific associations for circulating t-tau levels. The lead variants at the three loci identified at the genome-wide threshold in African American participants were extremely rare in European populations. In addition, most loci identified in African American participants were driven by the largest study, ARIC. Two of the findings identified at the genome-wide threshold in African Americans were low frequency variants, with no linkage disequilibrium support (Supplementary Figs. 15, 18), and with only two of the three cohorts that contributed to the meta-analysis. Caution is thus needed regarding the interpretation of these findings as such results are typically seen in GWAS of admixed populations with small sample sizes and could be driven by a few outliers. We also found that the strong association of the *MAPT* locus with circulating t-tau levels was specific to European-ancestry participants. This result is consistent with the recent finding from the Florida Consortium for African American Alzheimer’s Disease studies^57^. The majority of loci identified in European-ancestry participants were driven by the two largest studies, FHS and RSI. The fact that we did not detect an association in the African American participants for the novel loci detected at *P* < 5 × 10^−7^ in the European-ancestry participants may be due to a lack of power because of the limited sample size of this subgroup. However, our multi-ancestry meta-analysis showed that the hits identified were ancestry specific (Supplementary Table 3). The high heterogeneity observed across ancestry suggests that the genetic architecture of circulating t-tau levels may differ between European-ancestry and African American populations.

We explored the potential pleiotropy of loci previously reported for several neurological disorders with circulating t-tau levels by performing a look-up of these loci in our European meta-analysis. We also used MR analyses to evaluate the potential causal associations between circulating t-tau levels with several neurological disorders and traits, but we did not identify significant causal associations. Limitations of these analyses are the availability of large, complete, and publicly available GWAS summary statistics, and the strength of the MR instruments due to the limited number of associations in the European circulating t-tau meta-analysis and the limited number of AD and PD cases.

Strengths of our study are the large sample size, with the inclusion of eight population-based cohorts with ancestral diversity, with genotype data and information on circulating t-tau levels measured with ultra-sensitive assays. We leveraged large imputations reference panels (1000 Genomes and the Haplotype Reference Consortium) to study common genetic variations complemented with whole exome sequence data to explore less frequent genetic variations. Some limitations include the modest sample size of the African American sample that has limited our ability to confirm the GWAS findings and perform secondary analyses in this subgroup, such as stratification on *APOE4* status, and the fact that the contributed studies only had circulating t-tau measurement available, and not specifically phosphorylated tau. The fact that the *APOE* locus was not associated with circulating t-tau levels may suggest that circulating t-tau and phosphorylated tau have different genetic architectures. Replication of the African American potential novel loci and the less common variant association identified in Europeans is needed to confirm our findings but the availability of samples with circulating t-tau levels and genetic data is limited.

In conclusion, our large multi-ancestry meta-analysis identified new genetic variants and loci associated with circulating t-tau levels. Notably, our study revealed that the genetic architecture underlying circulating t-tau levels might differs between African American and European-ancestry populations and that genetic variation underlying circulating t-tau levels may overlap with known genetic determinants of neurological disorders. To better understand how these variants may contribute to AD and other tauopathies, further investigations of these findings will be necessary, including cohorts with a broader ancestral diversity, biological experiments, functional and omic studies, and animal models.

## Methods

### Populations and participants

We included in our multi-ancestry and ancestry-specific meta-analyses of total-tau participants from eight studies: seven cohorts from the Cohorts for heart and aging research in genomic epidemiology (CHARGE) consortium (the Framingham Heart Study (FHS), the Rotterdam Study (RSI and RSII), the MEMENTO Study, the Coronary Artery Risk Development in Young Adults (CARDIA) Study, the Cardiovascular Health Study (CHS), the Vietnam Era Twin Study of Aging (VETSA) Study, and the Atherosclerosis Risk in Communities (ARIC) Study), and the ADNI Study. All participants included in this study provided written informed consent for genetic testing and analyses. Study-specific information including study description, and detailed information about genotyping and imputations and GWAS analysis is included in the Supplementary Notes 1–8.

### Tau quantification

Circulating (in plasma or in serum) t-tau levels were quantified using the Human Total Tau kit on the Simoa™ HD-1 analyzer (ADNI, plasma), the Simoa™ Tau 2.0 Kit and the Simoa™ HD-1 analyzer (FHS, plasma), the Simoa™ Tau 2.0 Kit (MEMENTO_1, plasma), the Simoa™ Human Neurology 4-Plex A assay with the Simoa™ HD-X analyzer (the Atherosclerosis Risk in Communities study, MEMENTO_2 and the Coronary Artery Risk Development in Young Adults, plasma and the Cardiovascular Health Study, serum), the Simoa™ Human Neurology 3-Plex A assay with the Simoa™ HD-1 analyzer (the Rotterdam Study, plasma) and high throughput bioassays platforms or single analyte assays using the Simoa™ HD-X or Fujirebio analyzer (the Vietnam Era Twin Study of Aging, plasma).

### Genotyping and imputation

Studies used the densest imputation reference panel available to them at the time of analyses, either 1000 Genomes or the Haplotype Reference Consortium. A description of the reference panel used by each study is provided in Supplementary Tables 13–15.

### Genome-wide association studies (GWAS) and quality control

Each study evaluated the association of single-nucleotide genetic variants with log2-transformed circulating t-tau levels under an additive model. Analyses were adjusted for age, sex, and additional study specific covariates to control for population structure (including principal components). Studies with both Europeans and African Americans analyzed each ancestry separately. The minimum sample size for each ancestry/phenotype combination for inclusion in this study was fixed to 100. Additional stratified analyses according to *APOE4* status (*APOE4* carriers vs non *APOE*4 carriers) were performed in European participants only, given the modest sample size of the African American sample. Study-specific GWAS results were filtered based on an imputation quality score greater or equal to 0.30 and a minor allele count greater or equal to 20. The minor allele frequency threshold to include a genetic variant in the meta-analyses is indicated for each study in Supplementary Tables 13–15.

### Multi-ancestry and ancestry-specific meta-analyses

GWAS results across all studies were meta-analyzed by ancestry with METAL using an inverse variance-weighted average method^58^. For each meta-analysis, we selected results for which two third of the studies contributed. Results from the European and the African American meta-analyses, and for which at least two studies contributed to each meta-analysis, were then meta-analyzed using Metasoft^59,60^.

### Conditional and joint association analysis based on summary statistics

Conditional and joint association analysis in loci associated with circulating t-tau levels at the genome-wide threshold (*P* < 5 × 10^−8^) were performed using the Genome-wide Complex Trait Analysis^61,62^ based on the European meta-analysis results. A stepwise model selection procedure was used to select distinct associated genetic variants (*P* < 5 × 10^−8^). The FHS Haplotype Reference Consortium imputed data was used as the reference panel with unrelated participants only.

### Overlap of circulating t-tau genetic determinants with neurological diseases and traits

We extracted from the GWAS Catalog^20^ (https://www.ebi.ac.uk/gwas/downloads/summary-statistics) all reported associations for AD, t-tau (in plasma or CSF), stroke, PSP, Parkinson’s Disease (PD), and White Matter Hyperintensities (WMH), and looked them up in our European meta-analysis results. We also used the FUMA GWAS platform (Functional Mapping and Annotation of Genome-Wide Association Studies, https://fuma.ctglab.nl/) using as input the summary statistics from the European circulating t-tau meta-analysis to leverage functional, biological information to prioritize genes and check expression patterns and shared molecular functions between these genes^63,64^. Finally, we performed a GRS association analysis in the FHS. We first re-ran the European circulating t-tau levels meta-analysis without FHS. We then used the Genome-wide Complex Trait Analysis to identify distinct genome-wide associations on chromosome 17. We computed the GRS using the distinct variants identified by the Genome-wide Complex Trait Analysis for all FHS participants using the Haplotype Reference Consortium imputed genotypes and weights from the new meta-analysis. We tested the association of the GRS with incident AD (140 cases, 2775 controls) and stroke (149 cases, 3461 controls), and with four brain MRI phenotypes (hippocampal volume, white matter hyperintensities, total brain volume, and intracranial volume, N ranging from 3489 to 4310). Details regarding trait measurements and definitions in the FHS have been published elsewhere^65–67^. We used logistic or linear mixed-effects model, adjusted for age at baseline or at MRI and sex, while accounting for relatedness. For the brain MRI analyses, we excluded participants with dementia, stroke, large brain infarcts, tumor or any other finding that could have affected the scan and additionally adjusted the hippocampal volume, white matter hyperintensities, and total brain volume analyses for intracranial volume. If a significant association was detected (*P* = 0.05/6 = 0.008), an additional adjustment for *APOE4* was performed.

### Two sample mendelian randomization (MR) analyses

We used the TwoSampleMR R package in R^68,69^ to assess the causal association of circulating t-tau levels with AD, PD, stroke, and WMH using publicly available large European GWAS summary statistics. We selected large GWAS with independent samples from the ones included our meta-analysis. Briefly, we used an arbitrary threshold of *P* < 5 × 10^−6^ in the European meta-analysis of circulating t-tau levels to select the genetic variants to be used in the MR and performing clumping to select distinct variants based on 1000 Genomes European linkage disequilibrium reference panel. We then extracted these SNPs from the outcome GWAS based on summary statistics from the GWAS catalog (https://www.ebi.ac.uk/gwas/downloads/summary-statistics) or the IEU GWAS database (IGD, https://gwas.mrcieu.ac.uk/) and performed harmonization of the alleles. As *MAPT* locus is known to be pleiotropic, we conducted the mendelian randomization using different methods and carefully check for presence of heterogeneity and horizontal pleiotropy. For this analysis, we conducted power calculations for a continuous exposure and a PVE on the exposure of 8%, using the power analysis calculator https://sb452.shinyapps.io/power/. Finally, we tested the opposite hypothesis that AD, PD, stroke, and WMH are causally associated with circulating t-tau levels.

### Rare variant analyses using whole exome sequence data

To explore the association of rare variations with circulating t-tau levels, we selected the two largest studies (FHS and RSI) to perform rare-variant aggregation tests based on whole exome sequence data from the Cohorts for heart and aging research in genomic epidemiology (CHARGE) consortium^70^. A total of 2279 participants were included in the analyses. Information on sequencing and quality control is provided in the Supplementary Notes 9 and 10. To make sure that the same allele was coded as the effect allele for FHS and RSI, we used the effect (alternate) allele from a consensus SNP info file from the Cohorts for heart and aging research in genomic epidemiology (CHARGE) consortium^71^. Annotations of the exome variants was performed with dbNSFP^72^. We selected variants with a MAF ≤ 1% or 5%, and (1) missense and loss of function variants only or (2) variants with high or moderate impact from Ensembl Variant Effect Predictor^73^ including missense and loss of function variants. The analyses were performed using the R package seqMeta (http://cran.r-project.org/web/packages/seqMeta/). Each cohort used the seqMeta prepScores function to generate single variant score statistics and genotype covariance matrices for all variants. Results were then meta-analyzed using the skatMeta and burdenMeta functions. We used a Bonferroni correction for the number of genes included in the analyses and the number of tests (*P* = 0.05/20,000/2 = 1.25 × 10^−6^) and filtered the results based on a cumulative minor allele count of 30, that accounts for the number of genetic variants per gene.

### Reporting summary

Further information on research design is available in the Nature Research Reporting Summary linked to this article.

## Supplementary information

Supplementary Information

NR Reporting Summary

## Data Availability

All data supporting the findings of this study are available either within the main article or the supplementary information. Summary statistics from the ancestry-specific meta-analyses of circulating levels of total-tau have been deposited and are publicly accessible on the GWAS catalog FTP (study accession numbers GCST90095138, and GCST90095139). Genome-wide summary statistics for complex disorders used in the secondary analyses were downloaded from public repositories (GWAS catalog: https://www.ebi.ac.uk/gwas/downloads/summary-statistics; and IEU GWAS database: https://gwas.mrcieu.ac.uk/).
