# Supplementary material for: Meta-analysis of genome-wide association studies identifies ancestry-specific associations underlying circulating total tau levels
Source: Commun Biol. 2022 Apr 8;5:336. doi: 10.1038/s42003-022-03287-y (PMC8993877; doi:10.1038/s42003-022-03287-y)
Supplement: Supplementary file 1 — Supplementary Information [file 42003_2022_3287_MOESM1_ESM.pdf]

Supplementary Information

Meta-analysis of genome-wide association studies identifies ancestry-specific associations underlying circulating total tau levels

Chloé Sarnowski et al.

Table of Contents

Supplementary Figures 2
Supplementary Tables 33
Supplementary Notes 48
Supplementary References 73

Supplementary Figures

Supplementary Figure 1: Quantile-Quantile plots of association P-values for the meta-analysis of GWAS of circulating total-tau levels stratified by ancestry

A) African-American

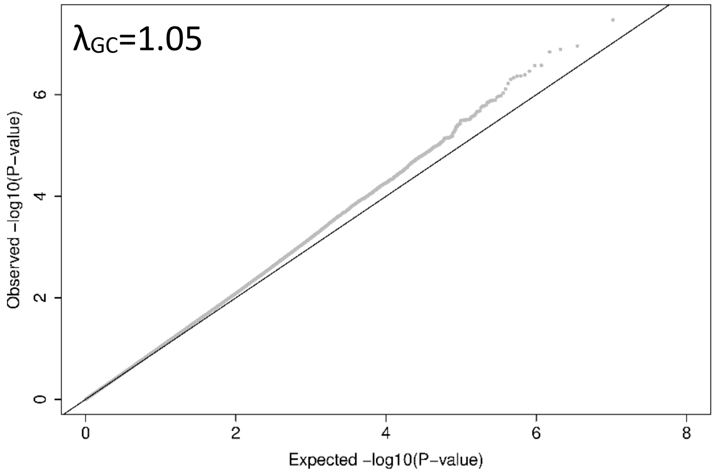

B) European-Ancestry

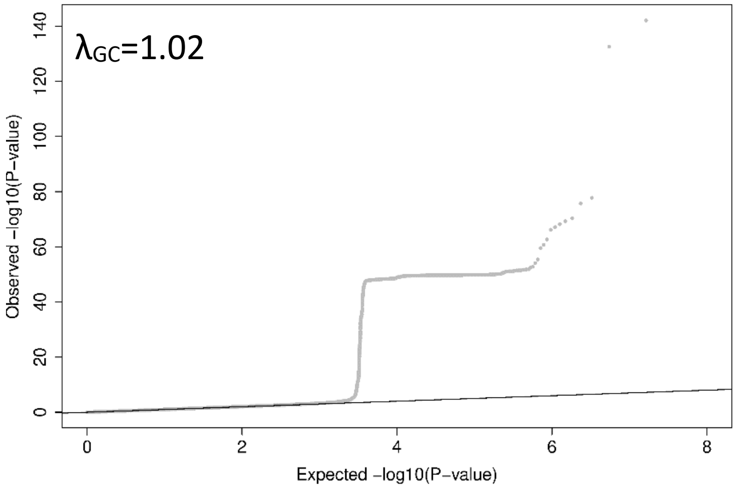

The dots represent the distribution of observed ordered –log10(*P*-values) against the theoretical model distribution of expected ordered –log10(*P*-values). The solid black line represents the theoretical model distribution of expected –log10(*P*-values) under the null distribution. λ_{GC} is the genomic inflation factor defined as the ratio of the median of the empirically observed distribution of the test statistic to the expected median, thus quantifying the extent of the inflation and the excess false positive rate.

Supplementary Figure 2: Forest plot for the lead genetic variant in *ADAMTS12* (T allele) in the African-ancestry meta-analysis of circulating total-tau levels.

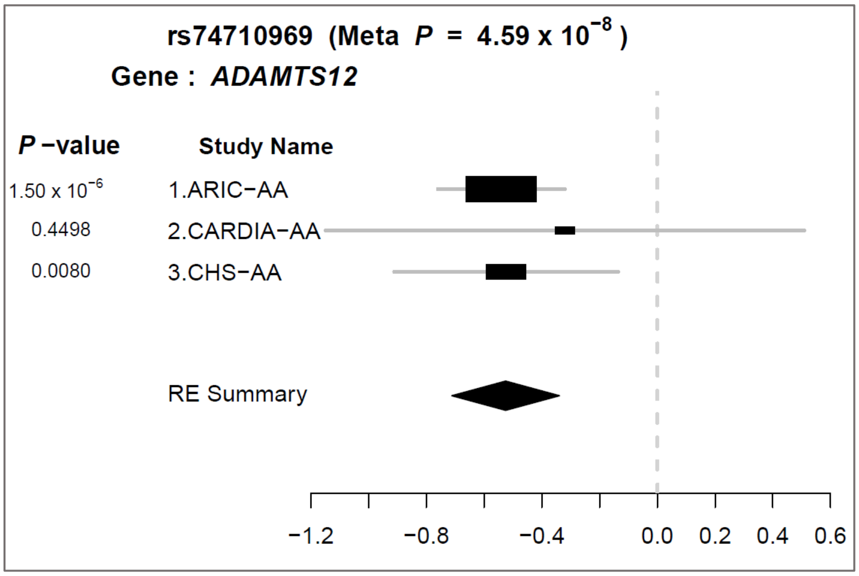

The plot was generated with ForestPMPlot based on Metasoft African-ancestry meta-analysis results. The meta-analysis P-value displayed corresponds to a random-effects model (very conservative). Each horizontal line represents an individual study with the effect size plotted as a box and the 95% confidence interval related to this effect size displayed as the line.

Supplementary Figure 3: Forest plot for the lead genetic variant near *IL15* (T allele) in the African-ancestry meta-analysis of circulating total-tau levels.

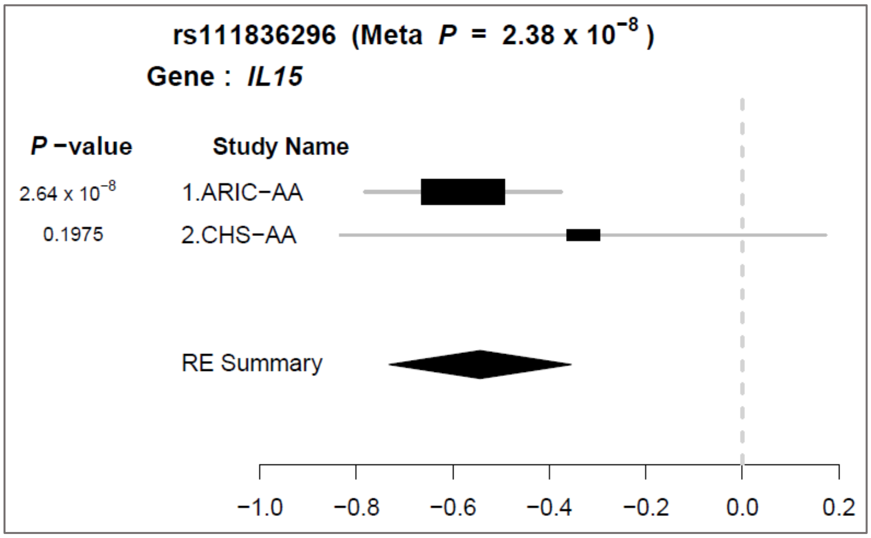

The plot was generated with ForestPMPlot based on Metasoft African-ancestry meta-analysis results. The meta-analysis P-value displayed corresponds to a random-effects model (very conservative). Each horizontal line represents an individual study with the effect size plotted as a box and the 95% confidence interval related to this effect size displayed as the line.

Supplementary Figure 4: Forest plot for the lead genetic variant in *FHIT* (A allele) in the African-ancestry meta-analysis of circulating total-tau levels.

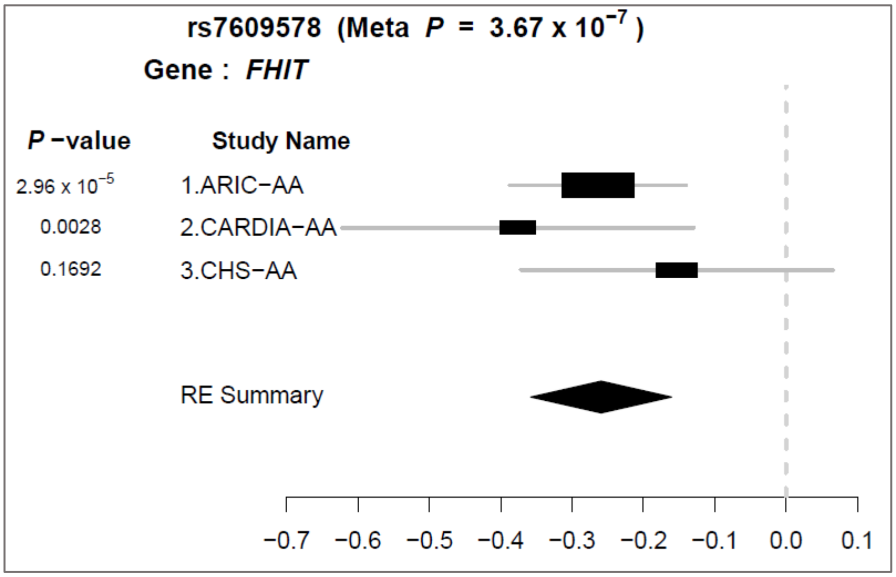

The plot was generated with ForestPMPlot based on Metasoft African-ancestry meta-analysis results. The meta-analysis P-value displayed corresponds to a random-effects model (very conservative). Each horizontal line represents an individual study with the effect size plotted as a box and the 95% confidence interval related to this effect size displayed as the line.

Supplementary Figure 5: Forest plot for the lead genetic variant in *PARD3* (A allele) in the African-ancestry meta-analysis of circulating total-tau levels.

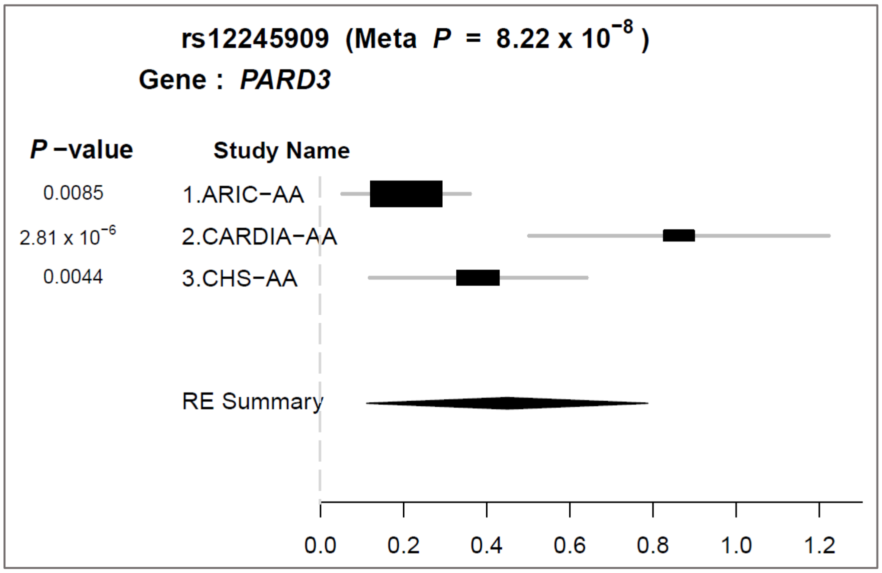

The plot was generated with ForestPMPlot based on Metasoft African-ancestry meta-analysis results. The meta-analysis P-value displayed corresponds to a random-effects model (very conservative). Each horizontal line represents an individual study with the effect size plotted as a box and the 95% confidence interval related to this effect size displayed as the line.

Supplementary Figure 6: Forest plot for the lead genetic variant in *MAP1B* (A allele) in the African-ancestry meta-analysis of circulating total-tau levels.

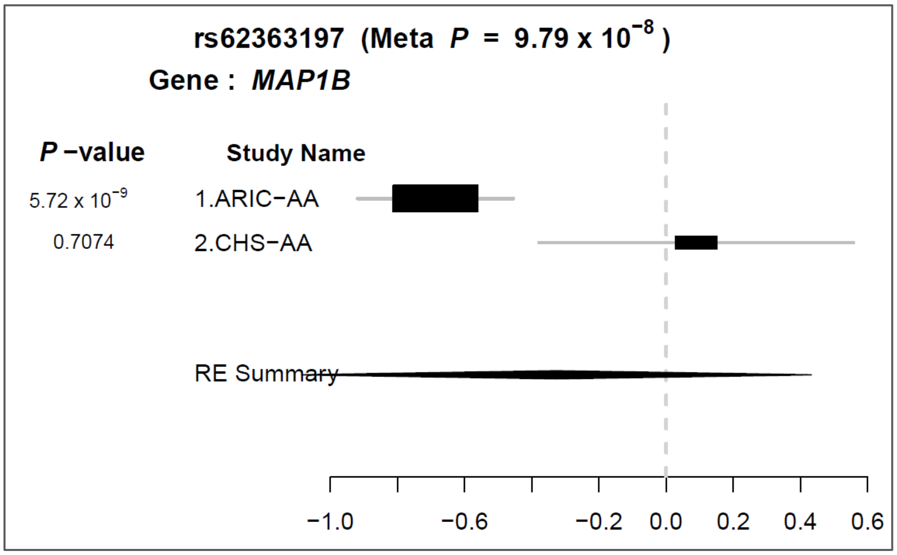

The plot was generated with ForestPMPlot based on Metasoft African-ancestry meta-analysis results. The meta-analysis P-value displayed corresponds to a random-effects model (very conservative). Each horizontal line represents an individual study with the effect size plotted as a box and the 95% confidence interval related to this effect size displayed as the line.

Supplementary Figure 7: Forest plot for the lead genetic variant in *AHNAK2* (A allele) in the African-ancestry meta-analysis of circulating total-tau levels.

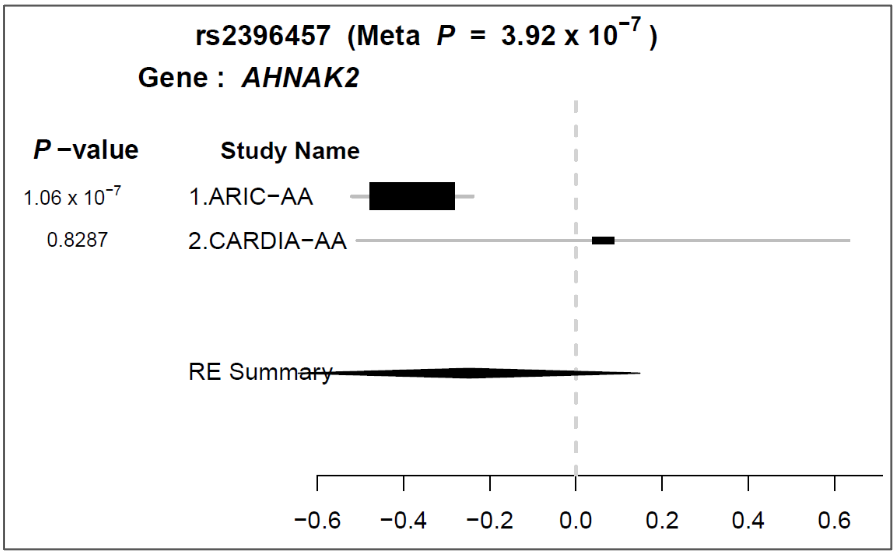

The plot was generated with ForestPMPlot based on Metasoft African-ancestry meta-analysis results. The meta-analysis P-value displayed corresponds to a random-effects model (very conservative). Each horizontal line represents an individual study with the effect size plotted as a box and the 95% confidence interval related to this effect size displayed as the line.

Supplementary Figure 8: Forest plot for the lead and known genetic variant in *MAPT* (A allele) in the European-ancestry meta-analysis of circulating total-tau levels.

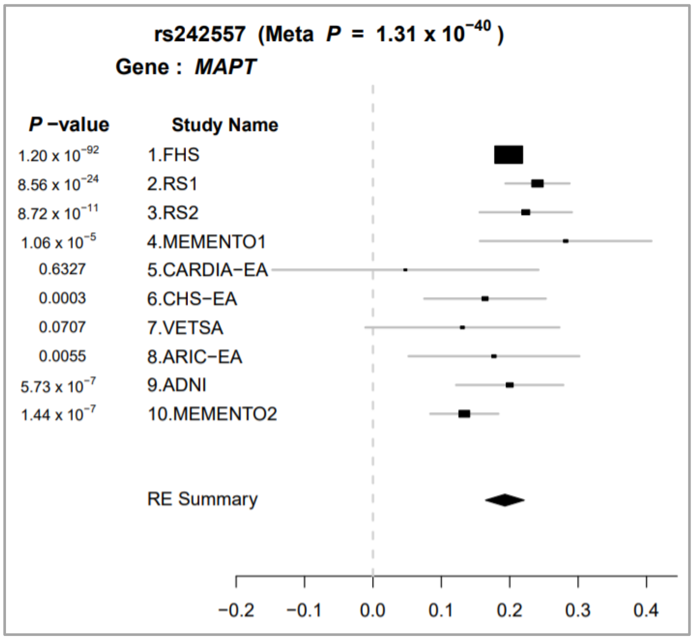

The plot was generated with ForestPMPlot based on Metasoft European-ancestry meta-analysis results. The meta-analysis P-value displayed corresponds to a random-effects model (very conservative). Each horizontal line represents an individual study with the effect size plotted as a box and the 95% confidence interval related to this effect size displayed as the line.

Supplementary Figure 9: Forest plot for the lead genetic variant in *F5* (A allele) in the European-ancestry meta-analysis of circulating total-tau levels.

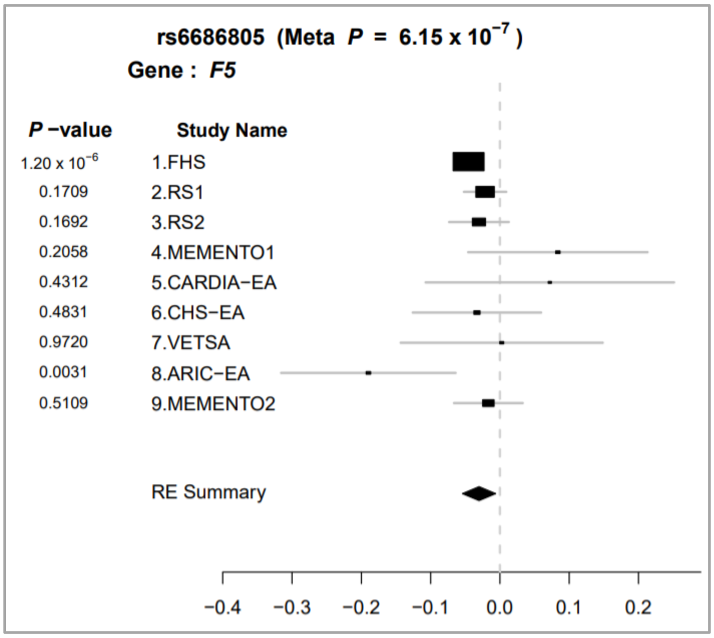

The plot was generated with ForestPMPlot based on Metasoft European-ancestry meta-analysis results. The meta-analysis P-value displayed corresponds to a random-effects model (very conservative). Each horizontal line represents an individual study with the effect size plotted as a box and the 95% confidence interval related to this effect size displayed as the line.

Supplementary Figure 10: Forest plot for the lead genetic variant in *SLC25A26* (A allele) in the European-ancestry meta-analysis of circulating total-tau levels.

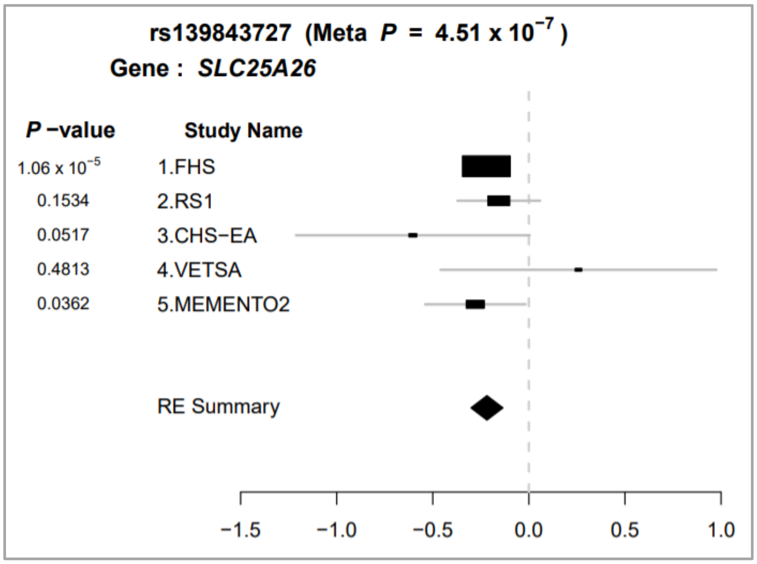

The plot was generated with ForestPMPlot based on Metasoft European-ancestry meta-analysis results. The meta-analysis P-value displayed corresponds to a random-effects model (very conservative). Each horizontal line represents an individual study with the effect size plotted as a box and the 95% confidence interval related to this effect size displayed as the line.

Supplementary Figure 11: Forest plot for the lead and known genetic variant in *BCAS3* (G allele) in the European-ancestry meta-analysis of circulating total-tau levels.

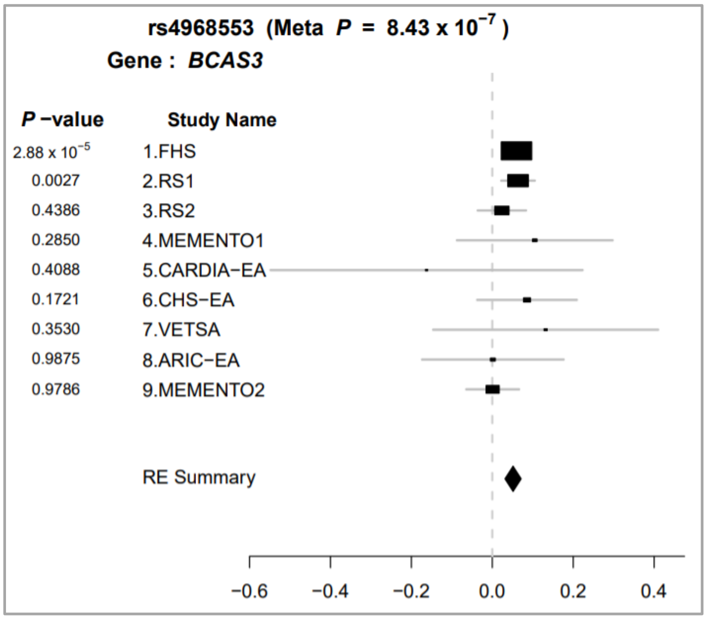

The plot was generated with ForestPMPlot based on Metasoft European-ancestry meta-analysis results. The meta-analysis P-value displayed corresponds to a random-effects model (very conservative). Each horizontal line represents an individual study with the effect size plotted as a box and the 95% confidence interval related to this effect size displayed as the line.

Supplementary Figure 12: Locuszoom regional association plot for the lead genetic variant on chr2 in the African-ancestry meta-analysis of circulating total-tau levels

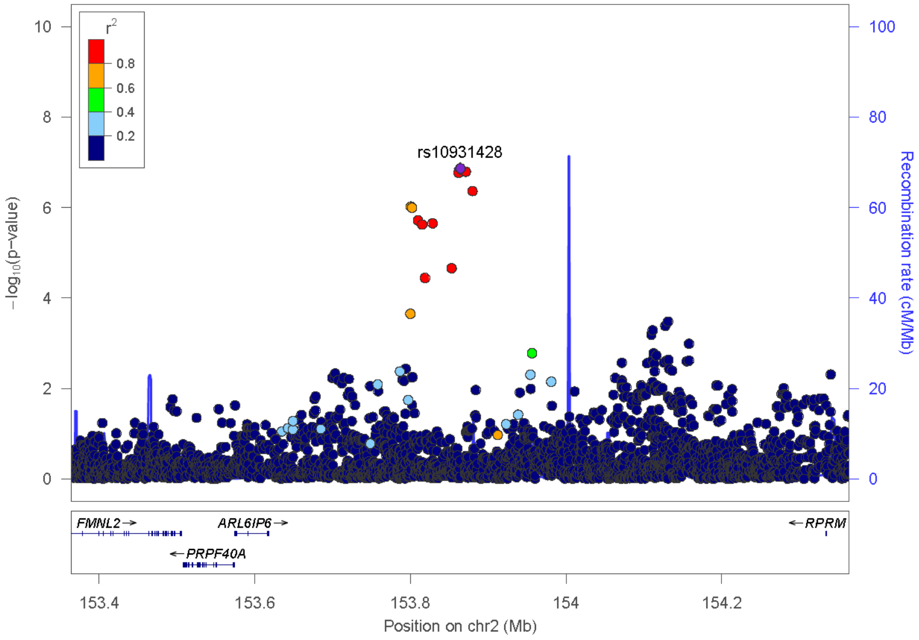

Single nucleotide variants are plotted with their P-values (-log10 values, left y-axis) as a function of build 37 genomic position on chromosome 2 (x-axis). Estimated recombination rates (right y-axis) are plotted to reflect the local linkage disequilibrium (LD) structure around the top associated single nucleotide variant (purple diamond) and correlated proxies (according to a blue to red scale from r2=0 to 1). LD was calculated in African ancestry samples from the 1000 Genomes Project.

Supplementary Figure 13: Locuszoom regional association plot for the lead genetic variant in *FHIT* in the African-ancestry meta-analysis of circulating total-tau levels

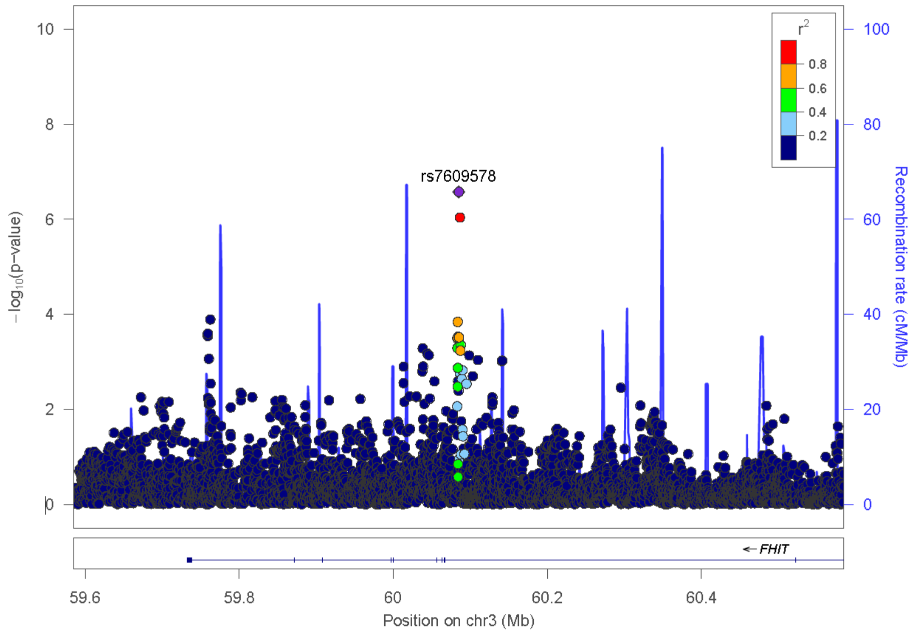

Single nucleotide variants are plotted with their P-values (-log10 values, left y-axis) as a function of build 37 genomic position on chromosome 3 (x-axis). Estimated recombination rates (right y-axis) are plotted to reflect the local linkage disequilibrium (LD) structure around the top associated single nucleotide variant (purple diamond) and correlated proxies (according to a blue to red scale from r2=0 to 1). LD was calculated in African ancestry samples from the 1000 Genomes Project.

Supplementary Figure 14: Locuszoom regional association plot for the lead genetic variant on chr3 in the African-ancestry meta-analysis of circulating total-tau levels

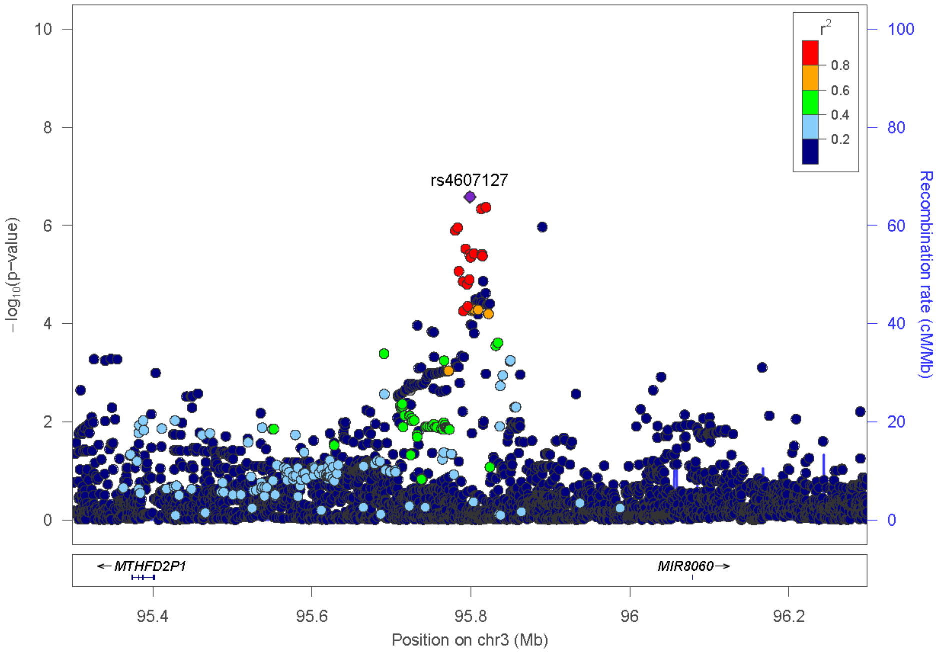

Single nucleotide variants are plotted with their P-values (-log10 values, left y-axis) as a function of build 37 genomic position on chromosome 3 (x-axis). Estimated recombination rates (right y-axis) are plotted to reflect the local linkage disequilibrium (LD) structure around the top associated single nucleotide variant (purple diamond) and correlated proxies (according to a blue to red scale from r2=0 to 1). LD was calculated in African ancestry samples from the 1000 Genomes Project.

Supplementary Figure 15: Locuszoom regional association plot for the lead genetic variant on chr4 in the African-ancestry meta-analysis of circulating total-tau levels

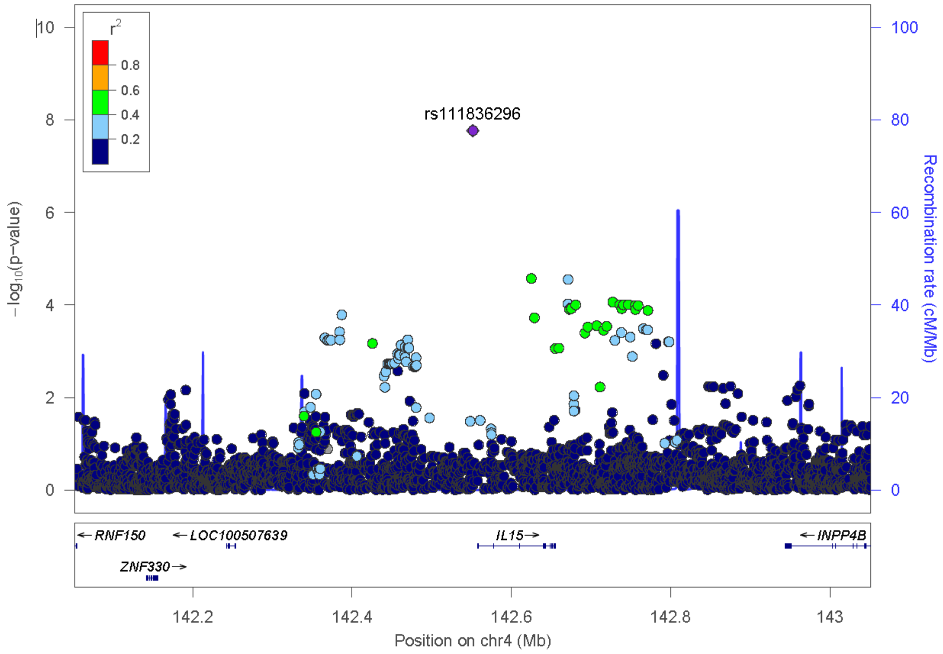

Single nucleotide variants are plotted with their P-values (-log10 values, left y-axis) as a function of build 37 genomic position on chromosome 4 (x-axis). Estimated recombination rates (right y-axis) are plotted to reflect the local linkage disequilibrium (LD) structure around the top associated single nucleotide variant (purple diamond) and correlated proxies (according to a blue to red scale from r2=0 to 1). LD was calculated in African ancestry samples from the 1000 Genomes Project.

Supplementary Figure 16: Locuszoom regional association plot for the lead genetic variant in *ADAMTS12* in the African-ancestry meta-analysis of circulating total-tau levels

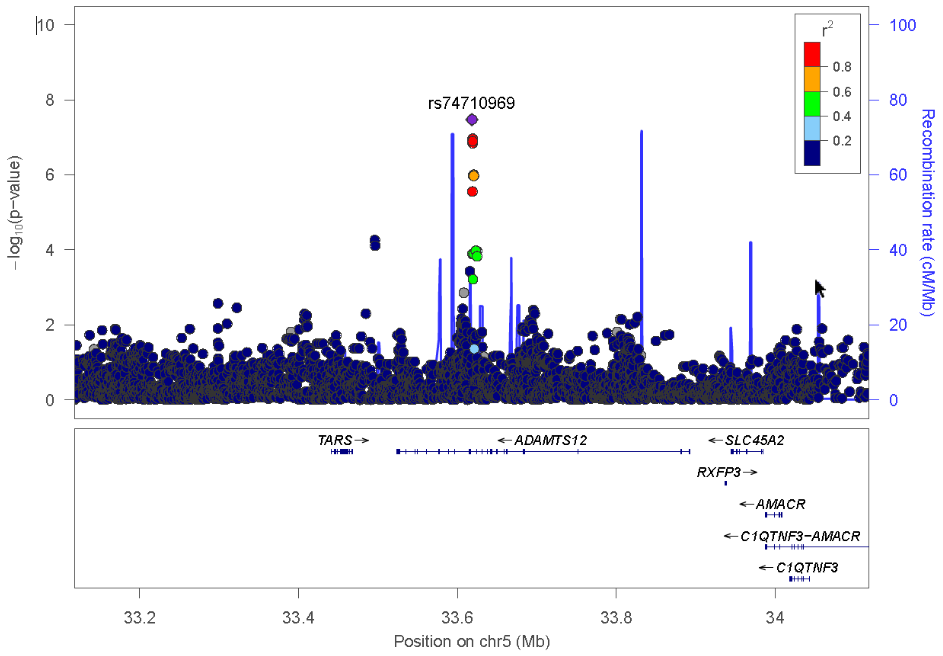

Single nucleotide variants are plotted with their P-values (-log10 values, left y-axis) as a function of build 37 genomic position on chromosome 5 (x-axis). Estimated recombination rates (right y-axis) are plotted to reflect the local linkage disequilibrium (LD) structure around the top associated single nucleotide variant (purple diamond) and correlated proxies (according to a blue to red scale from r2=0 to 1). LD was calculated in African ancestry samples from the 1000 Genomes Project.

Supplementary Figure 17: Locuszoom regional association plot for the lead genetic variant in *MAP1B* in the African-ancestry meta-analysis of circulating total-tau levels

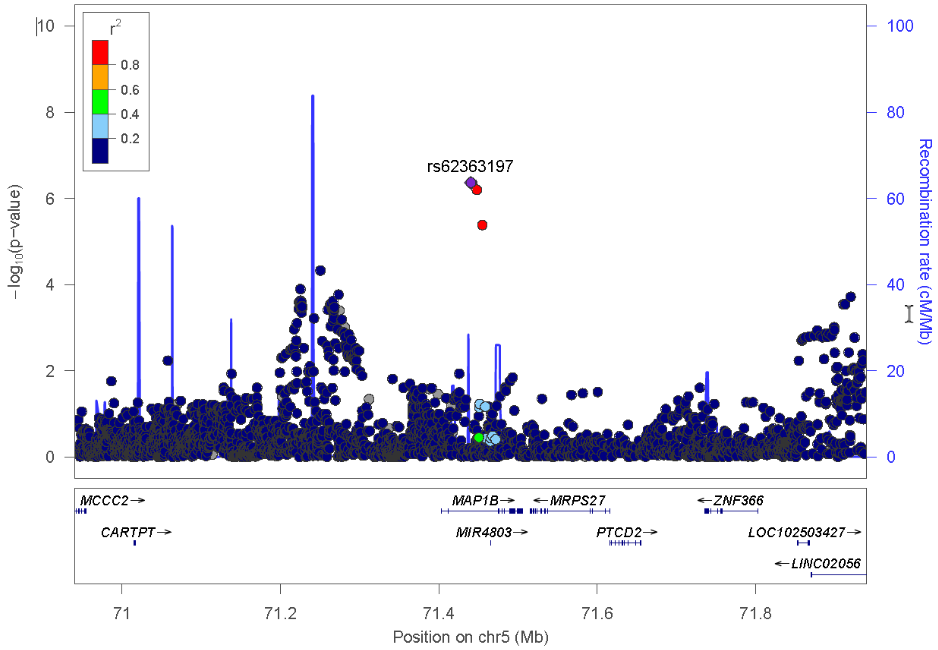

Single nucleotide variants are plotted with their P-values (-log10 values, left y-axis) as a function of build 37 genomic position on chromosome 5 (x-axis). Estimated recombination rates (right y-axis) are plotted to reflect the local linkage disequilibrium (LD) structure around the top associated single nucleotide variant (purple diamond) and correlated proxies (according to a blue to red scale from r2=0 to 1). LD was calculated in African ancestry samples from the 1000 Genomes Project.

Supplementary Figure 18: Locuszoom regional association plot for the lead genetic variant on chr6 in the African-ancestry meta-analysis of circulating total-tau levels

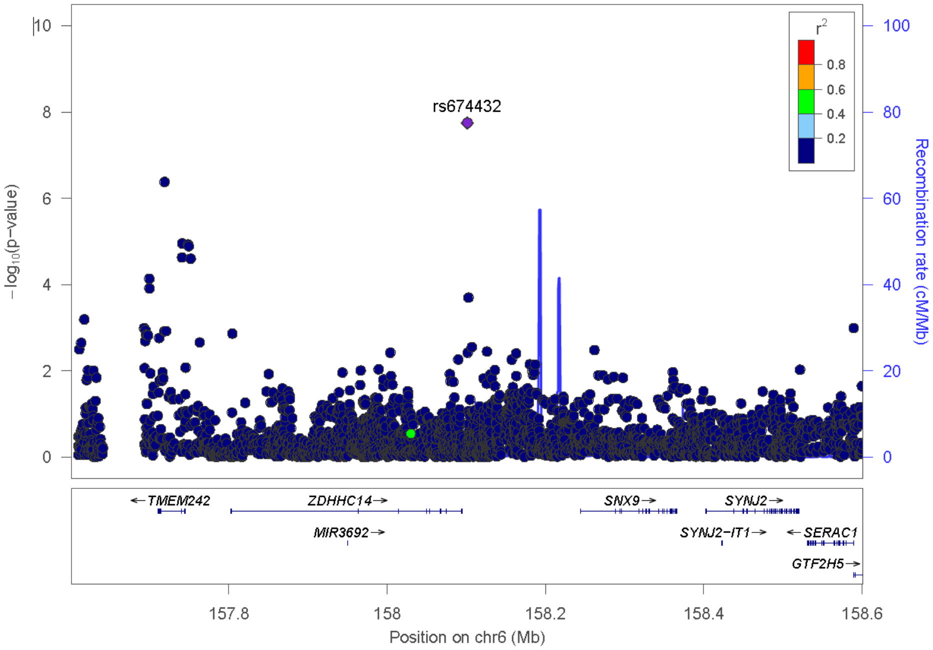

Single nucleotide variants are plotted with their P-values (-log10 values, left y-axis) as a function of build 37 genomic position on chromosome 6 (x-axis). Estimated recombination rates (right y-axis) are plotted to reflect the local linkage disequilibrium (LD) structure around the top associated single nucleotide variant (purple diamond) and correlated proxies (according to a blue to red scale from r2=0 to 1). LD was calculated in African ancestry samples from the 1000 Genomes Project.

Supplementary Figure 19: Locuszoom regional association plots for the lead genetic variants on chr7 in the African-ancestry meta-analysis of circulating total-tau levels

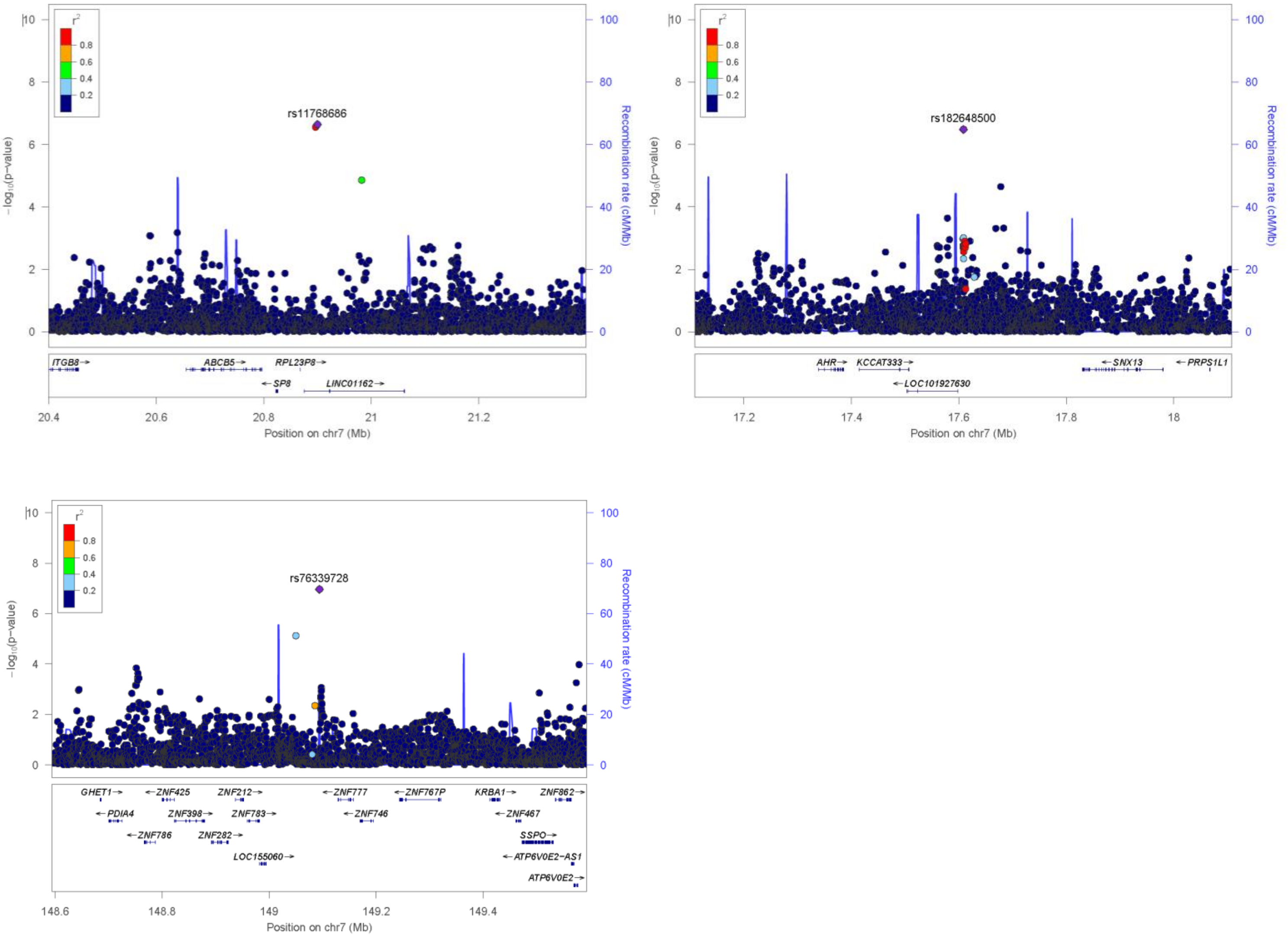

Single nucleotide variants are plotted with their P-values (-log10 values, left y-axis) as a function of build 37 genomic position on chromosome 7 (x-axis). Estimated recombination rates (right y-axis) are plotted to reflect the local linkage disequilibrium (LD) structure around the top associated single nucleotide variant (purple diamond) and correlated proxies (according to a blue to red scale from r2=0 to 1). LD was calculated in African ancestry samples from the 1000 Genomes Project.

Supplementary Figure 20: Locuszoom regional association plots for the lead genetic variant on chr8 in the African-ancestry meta-analysis of circulating total-tau levels

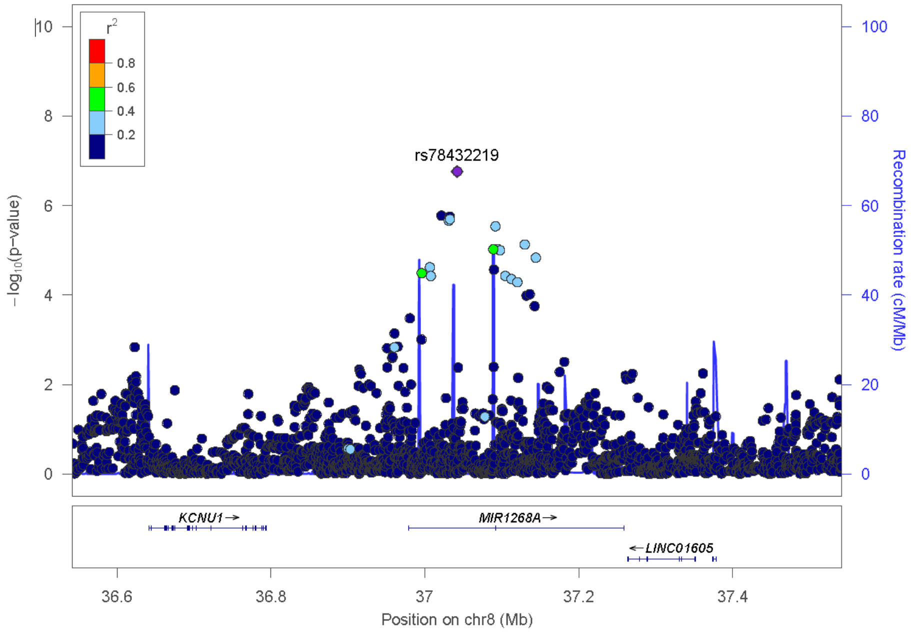

Single nucleotide variants are plotted with their P-values (-log10 values, left y-axis) as a function of build 37 genomic position on chromosome 8 (x-axis). Estimated recombination rates (right y-axis) are plotted to reflect the local linkage disequilibrium (LD) structure around the top associated single nucleotide variant (purple diamond) and correlated proxies (according to a blue to red scale from r2=0 to 1). LD was calculated in African ancestry samples from the 1000 Genomes Project.

Supplementary Figure 21: Locuszoom regional association plots for the lead genetic variant in *PARD3* in the African-ancestry meta-analysis of circulating total-tau levels

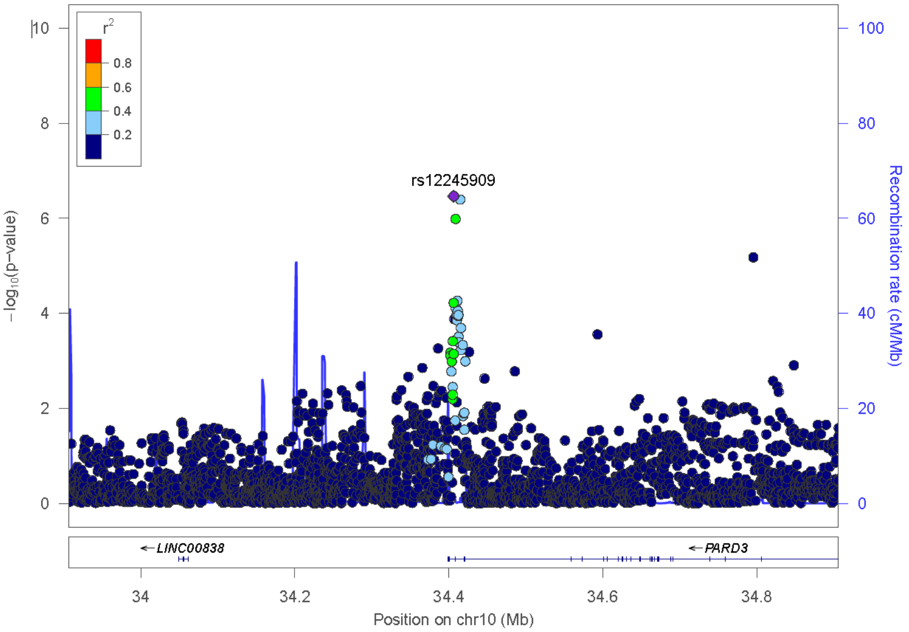

Single nucleotide variants are plotted with their P-values (-log10 values, left y-axis) as a function of build 37 genomic position on chromosome 10 (x-axis). Estimated recombination rates (right y-axis) are plotted to reflect the local linkage disequilibrium (LD) structure around the top associated single nucleotide variant (purple diamond) and correlated proxies (according to a blue to red scale from r2=0 to 1). LD was calculated in African ancestry samples from the 1000 Genomes Project.

Supplementary Figure 22: Locuszoom regional association plots for the lead genetic variant on chr10 in the African-ancestry meta-analysis of circulating total-tau levels

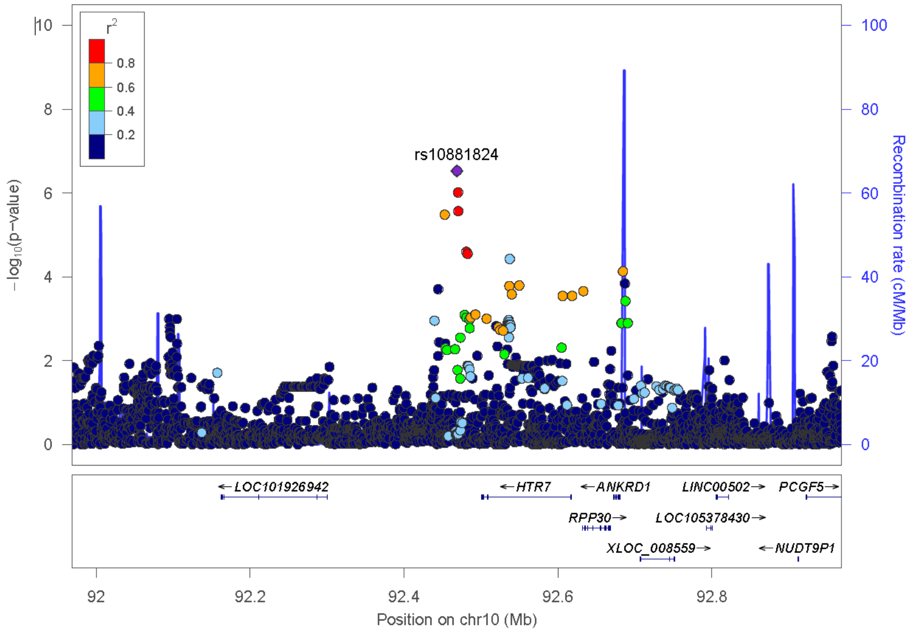

Single nucleotide variants are plotted with their P-values (-log10 values, left y-axis) as a function of build 37 genomic position on chromosome 10 (x-axis). Estimated recombination rates (right y-axis) are plotted to reflect the local linkage disequilibrium (LD) structure around the top associated single nucleotide variant (purple diamond) and correlated proxies (according to a blue to red scale from r2=0 to 1). LD was calculated in African ancestry samples from the 1000 Genomes Project.

Supplementary Figure 23: Locuszoom regional association plots for the lead genetic variant in *AHNAK2* in the African-ancestry meta-analysis of circulating total-tau levels

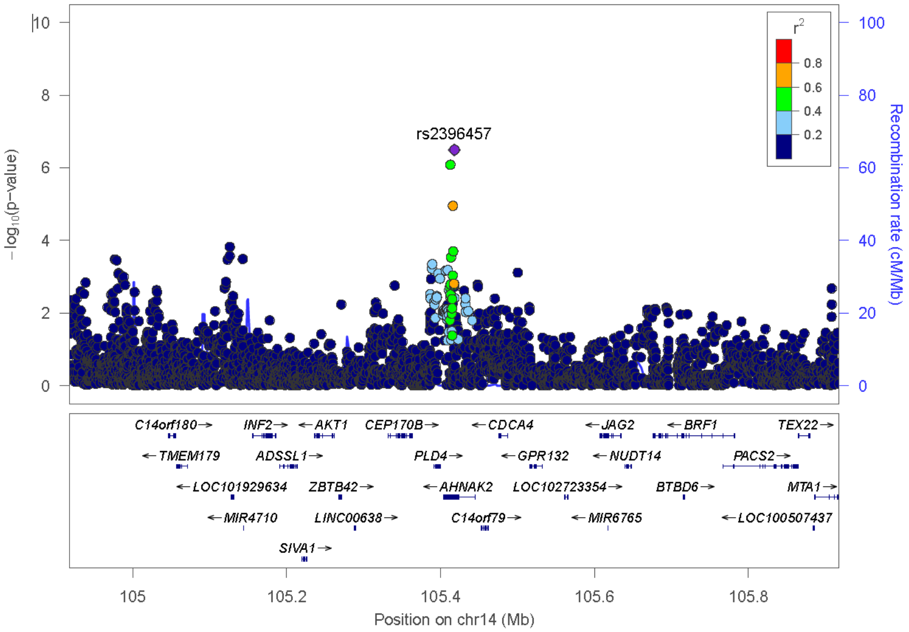

Single nucleotide variants are plotted with their P-values (-log10 values, left y-axis) as a function of build 37 genomic position on chromosome 14 (x-axis). Estimated recombination rates (right y-axis) are plotted to reflect the local linkage disequilibrium (LD) structure around the top associated single nucleotide variant (purple diamond) and correlated proxies (according to a blue to red scale from r2=0 to 1). LD was calculated in African ancestry samples from the 1000 Genomes Project.

Supplementary Figure 24: Locuszoom regional association plots for the lead genetic variant in *F5* in the European-ancestry meta-analysis of circulating total-tau levels

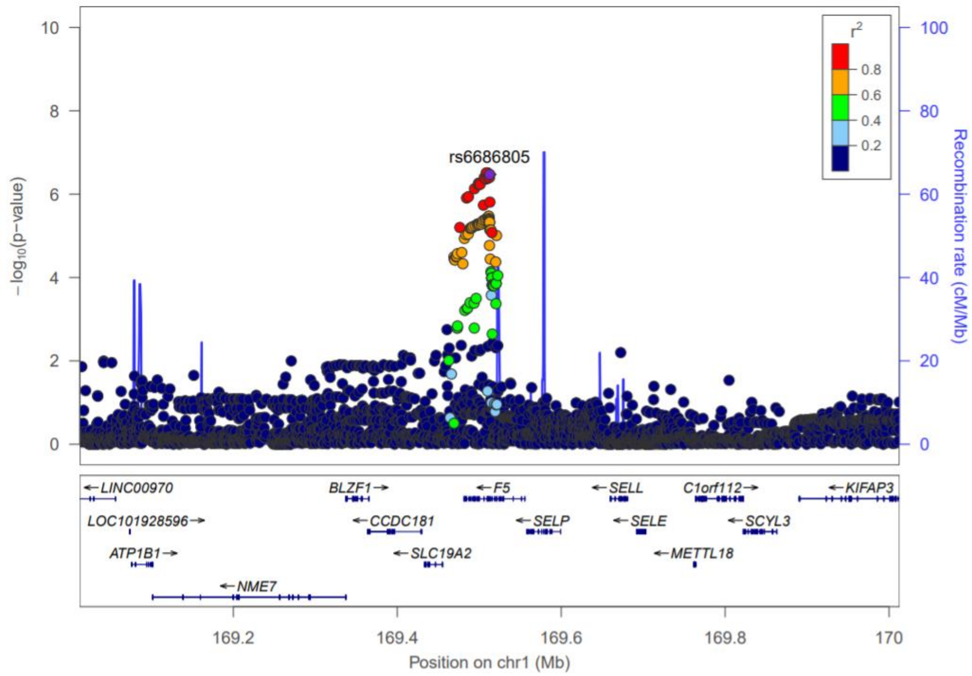

Single nucleotide variants are plotted with their P-values (-log10 values, left y-axis) as a function of build 37 genomic position on chromosome 1 (x-axis). Estimated recombination rates (right y-axis) are plotted to reflect the local linkage disequilibrium (LD) structure around the top associated single nucleotide variant (purple diamond) and correlated proxies (according to a blue to red scale from r2=0 to 1). LD was calculated in European ancestry samples from the 1000 Genomes Project.

Supplementary Figure 25: Locuszoom regional association plots for the lead genetic variant in *SLC25A26* in the European-ancestry meta-analysis of circulating total-tau levels

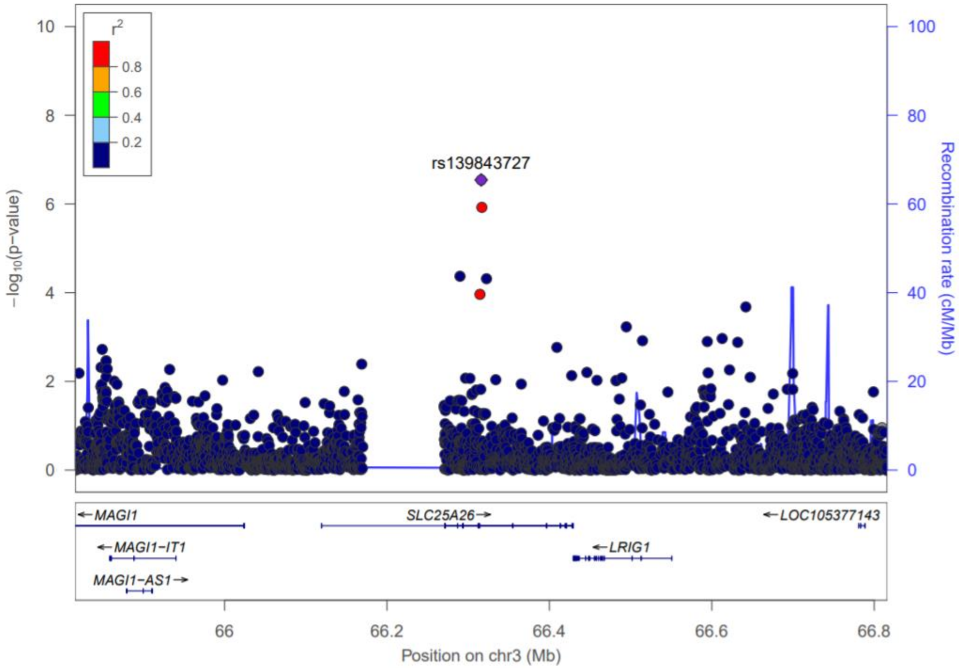

Single nucleotide variants are plotted with their P-values (-log10 values, left y-axis) as a function of build 37 genomic position on chromosome 3 (x-axis). Estimated recombination rates (right y-axis) are plotted to reflect the local linkage disequilibrium (LD) structure around the top associated single nucleotide variant (purple diamond) and correlated proxies (according to a blue to red scale from r2=0 to 1). LD was calculated in European ancestry samples from the 1000 Genomes Project.

Supplementary Figure 26: Locuszoom regional association plots for the lead genetic variant in *MAPT* in the European-ancestry meta-analysis of circulating total-tau levels

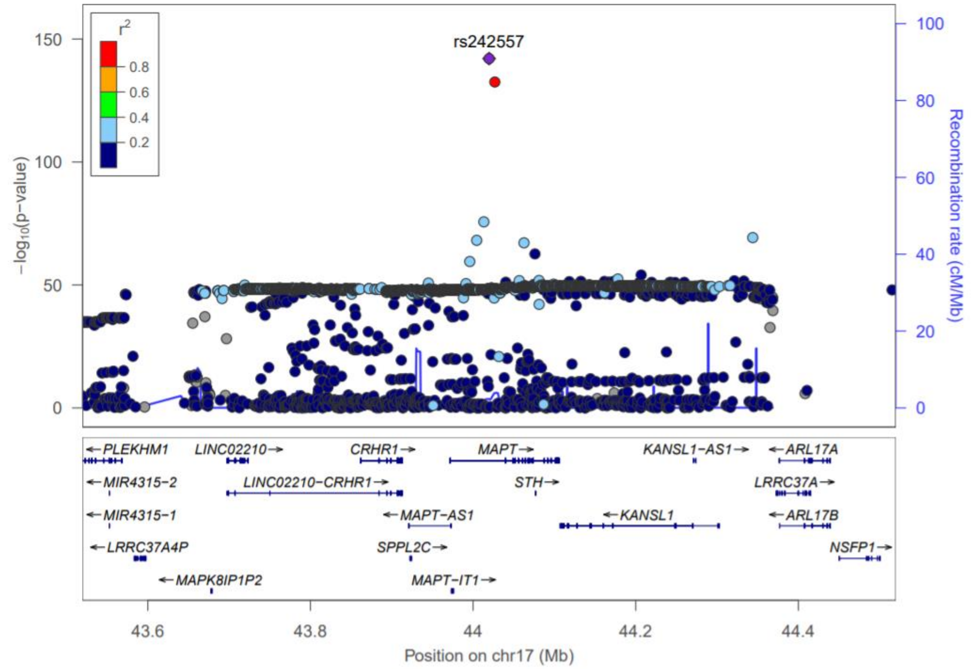

Single nucleotide variants are plotted with their P-values (-log10 values, left y-axis) as a function of build 37 genomic position on chromosome 17 (x-axis). Estimated recombination rates (right y-axis) are plotted to reflect the local linkage disequilibrium (LD) structure around the top associated single nucleotide variant (purple diamond) and correlated proxies (according to a blue to red scale from r2=0 to 1). LD was calculated in European ancestry samples from the 1000 Genomes Project.

Supplementary Figure 27: Locuszoom regional association plots for the lead genetic variant in *BCAS3* in the European-ancestry meta-analysis of circulating total-tau levels

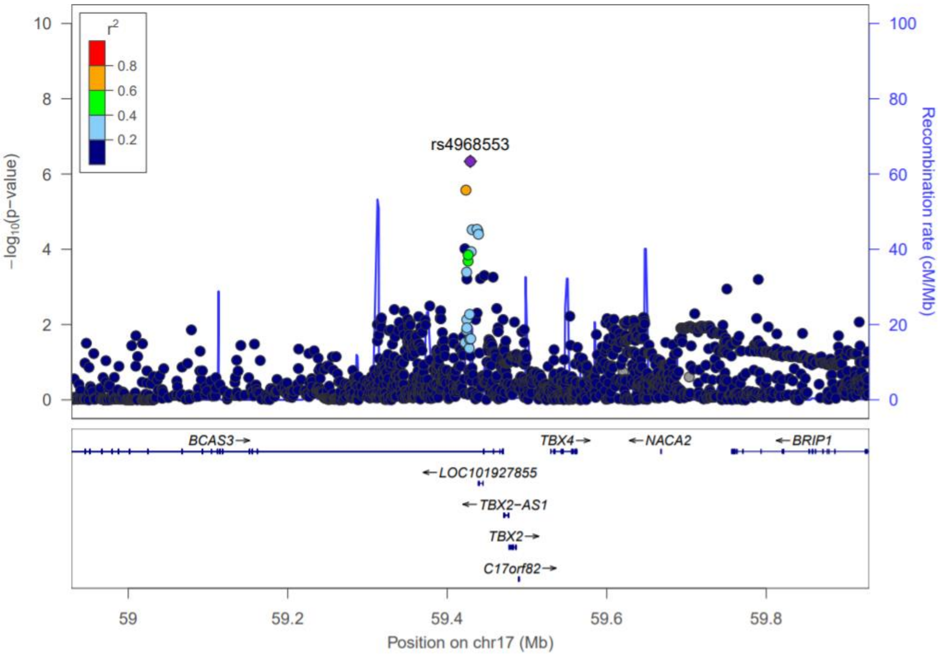

Single nucleotide variants are plotted with their P-values (-log10 values, left y-axis) as a function of build 37 genomic position on chromosome 17 (x-axis). Estimated recombination rates (right y-axis) are plotted to reflect the local linkage disequilibrium (LD) structure around the top associated single nucleotide variant (purple diamond) and correlated proxies (according to a blue to red scale from r2=0 to 1). LD was calculated in European ancestry samples from the 1000 Genomes Project.

Supplementary Figure 28: Differentially expressed genes (DEG) based on 54 tissue types from the Genotype Tissue Expression (GTEx v8) project. Analysis was performed with the Functional Mapping and Annotation of Genome-Wide Association Studies (FUMA GWAS) platform, based on genome-wide signals (P=5×10^{-8}) from the circulating total-tau meta-analysis in Europeans.

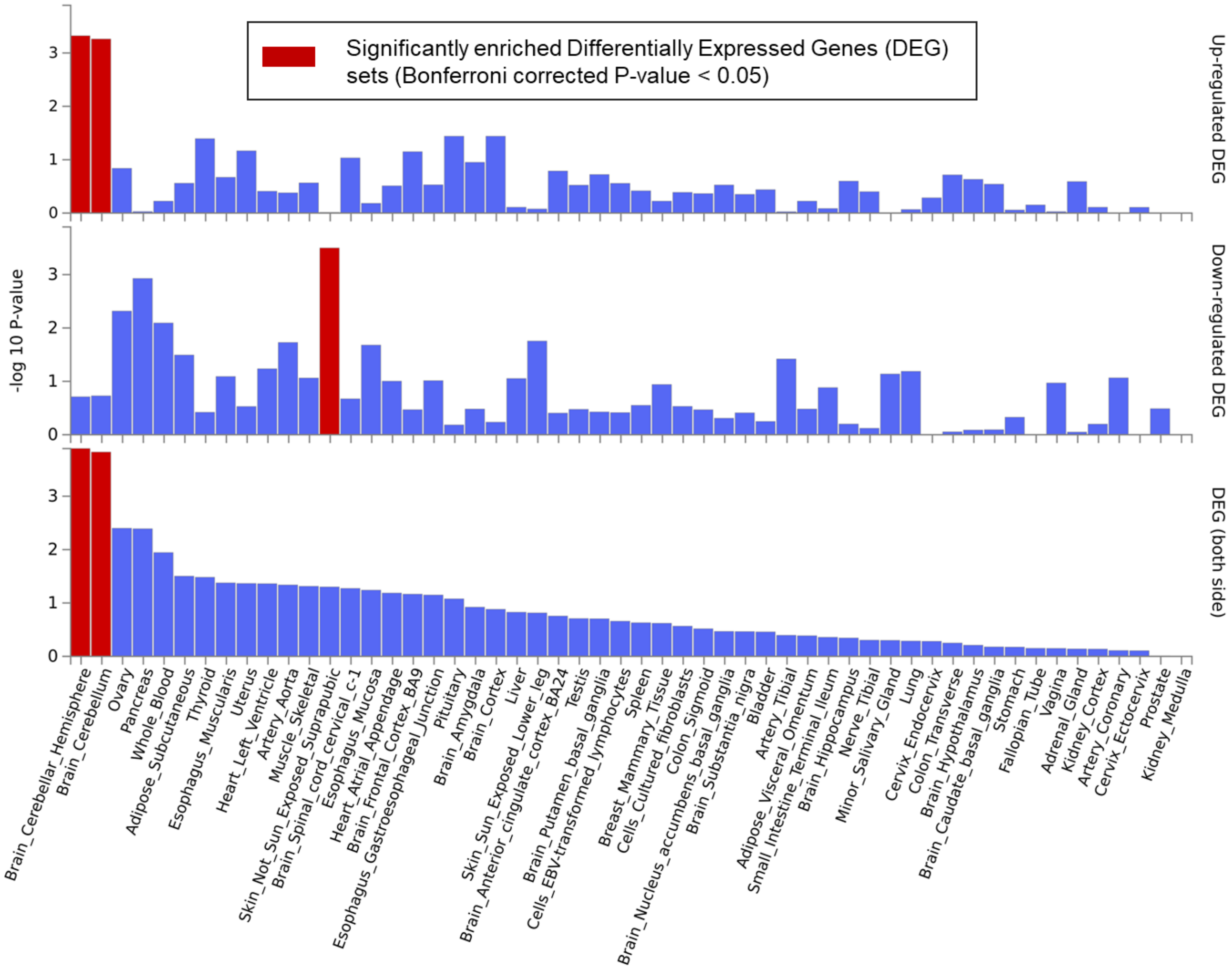

Supplementary Figure 29: Enrichment analysis of input genes in Gene Sets based on GWAS catalog reported genes. Analysis was performed with the Functional Mapping and Annotation of Genome-Wide Association Studies (FUMA GWAS) platform, based on the main signals from the circulating total-tau meta-analysis in Europeans.

A) Input: signals detected at P=5×10^{-8}

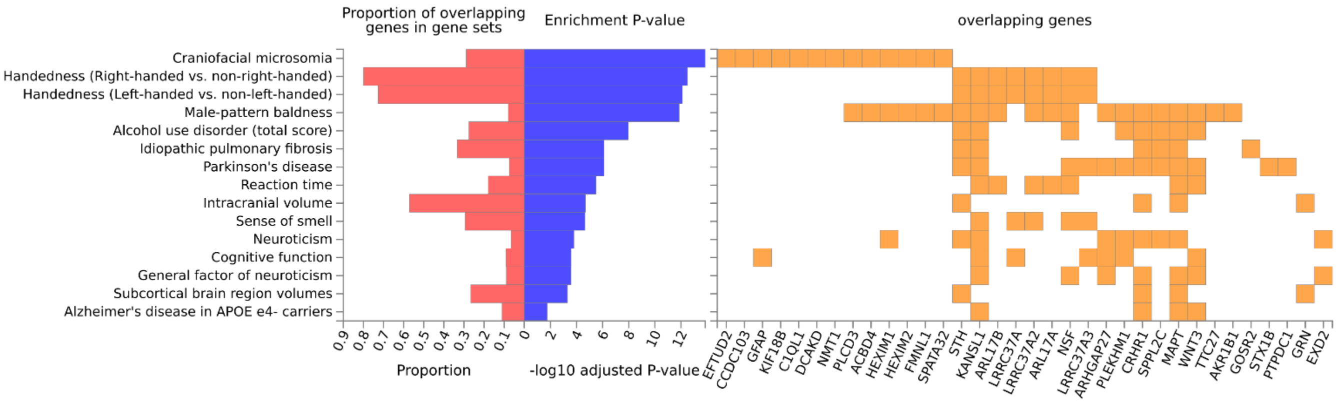

B) Input: signals detected at P=5×10^{-6}

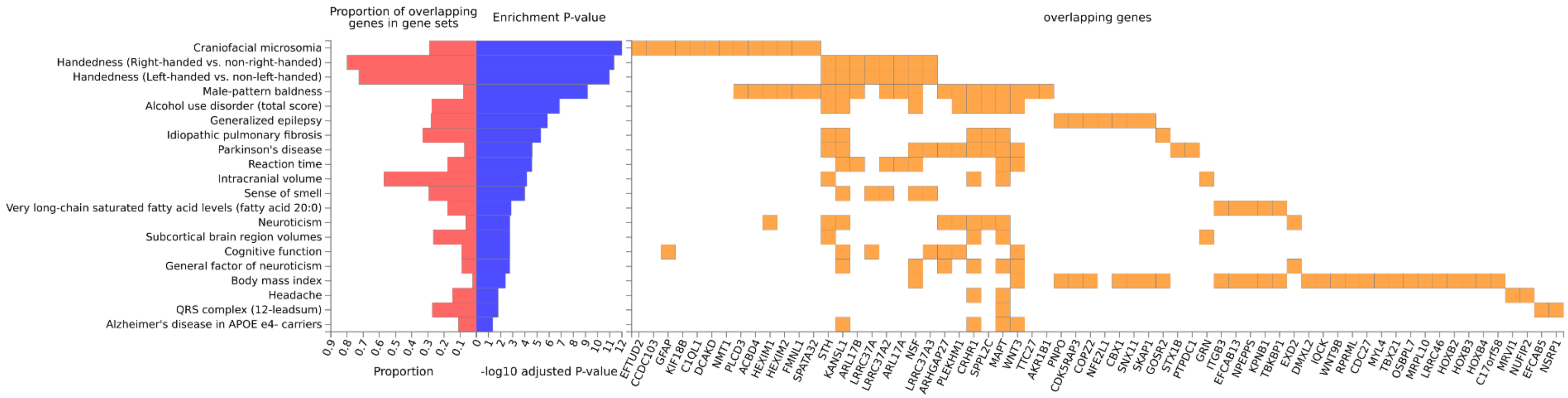

Supplementary Figure 30: Manhattan plots of association P-values for the rare variant aggregation analyses of circulating total-tau levels based on whole exome sequence data and using missense and loss of function variants

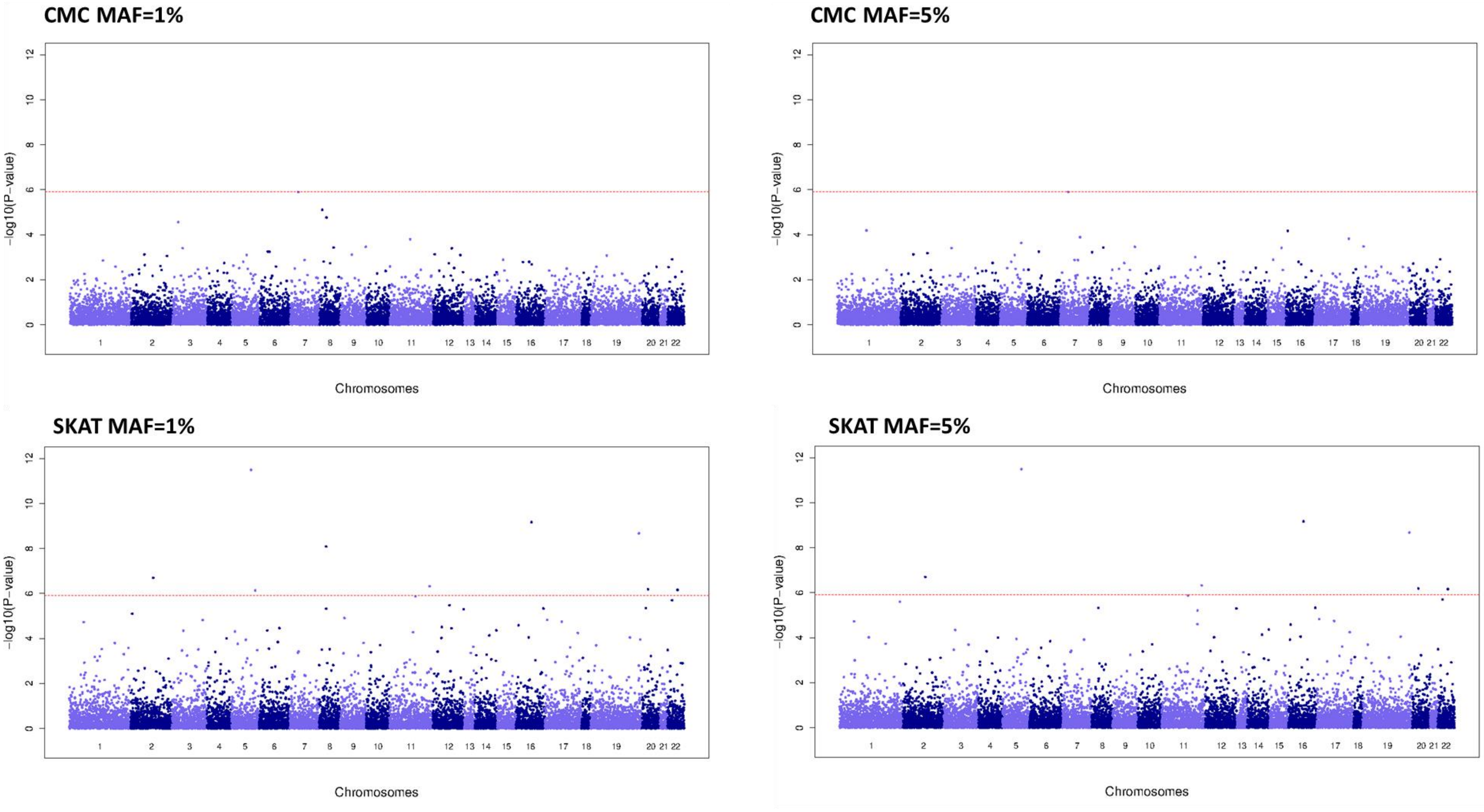

The –log10(*P*)-value for each gene on the y-axis is plotted against the build 37 genomic position on the x-axis (chromosomal coordinate). The dashed horizontal red line indicates the gene level significance threshold of $P = 0.05/20{,}000/2 = 1.25\times10^{-6}$.

Supplementary Figure 31: Manhattan plots of association P-values for the rare variant aggregation analyses of circulating total-tau levels based on whole exome sequence data and using high or moderate impact variants based on Ensembl Variant Effect Predictor functional annotations

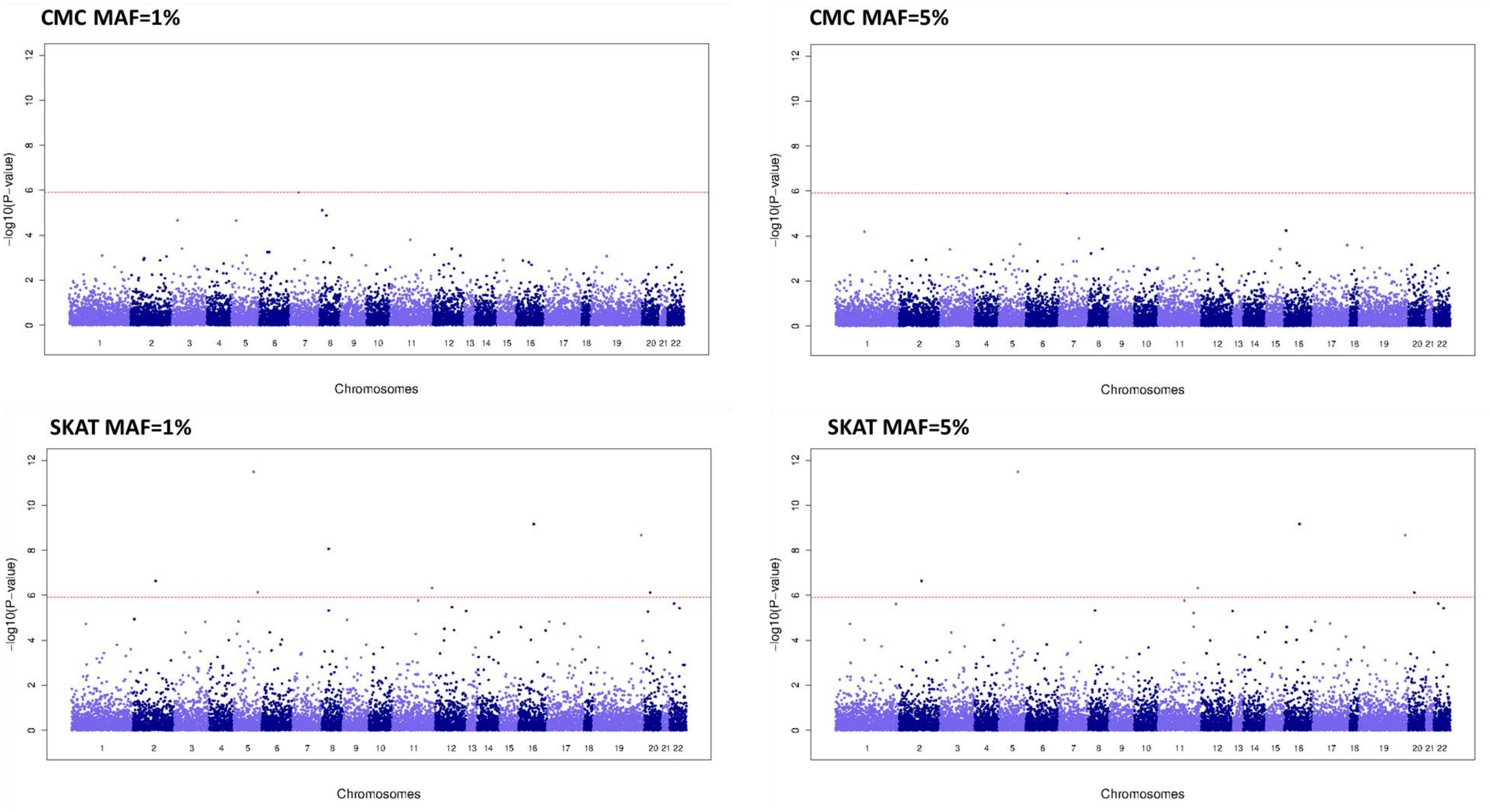

The –log10(P)-value for each gene on the y-axis is plotted against the build 37 genomic position on the x-axis (chromosomal coordinate). The dashed horizontal red line indicates the gene level significance threshold of P= 0.05/20,000/2 = 1.25×10^{-6}.

Supplementary Tables

Supplementary Table 1: Stepwise model selection procedure performed based on the circulating total-tau levels European meta-analysis summary statistics using the Framingham Heart Study Haplotype Reference Consortium imputations as the reference panel, excluding related participants

rsid	Chr	Build 37 Pos (bp)	Effect Allele	Freq	Beta	SE	*P**	N	Freq Geno	BJ	BJ_SE	PJ	LD_r
rs7502280	17	43,670,221	T	0.88	0.17	0.01	5.7E-38	9085.09	0.89	0.09	0.01	2.3E-11	0.25
rs242557	17	44,019,712	A	0.38	0.20	0.01	2.3E-143	11986.3	0.37	0.16	0.01	1.4E-69	0.46
rs2942003	17	44,576,704	T	0.34	0.16	0.01	3.9E-78	9950.89	0.29	0.08	0.01	9.8E-16	0.00

BJ, BJ_SE, PJ: conditional analysis effect sizes, standard errors and P-values respectively

*P: P-values displayed slightly vary from P-values presented in the original European ancestry meta-analysis due to rounded beta and SE values used by the Genome-wide Complex Trait Analysis (https://cnsgenomics.com/software/gcta/#Overview)

Supplementary Table 2: Ancestry-specific meta-analysis results of circulating total-tau levels for the lead genetic variants in each locus passing the threshold of $5×10^{-8}<P<5×10^{-7}$

rsid	Chr	Build 37 Pos (bp)	Eff	NEff	EAF	Beta	SE	*P*	I^2	P_Q	Gene
**African-Americans**											
rs10931428	2	153,864,017	A	T	0.05	-0.55	0.10	1.4E-07	0	0.46	intergenic
rs7609578	3	60,084,971	A	G	0.40	-0.26	0.05	2.7E-07	0	0.41	*FHIT*
rs4607127	3	95,798,669	A	G	0.18	-0.32	0.06	2.6E-07	0	0.95	intergenic
rs62363197	5	71,440,155	A	G	0.05	-0.53	0.11	4.3E-07	88.3	0.004	*MAP1B*
rs182648500	7	17,608,415	C	T	0.03	-0.65	0.13	3.3E-07	0	0.53	intergenic
rs11768686	7	20,899,530	A	G	0.04	-0.57	0.11	2.3E-07	0	0.68	intergenic
rs76339728	7	149,094,017	T	C	0.95	0.63	0.12	1.1E-07	36.9	0.21	intergenic
rs78432219	8	37,041,938	A	G	0.06	-0.46	0.09	1.7E-07	44.7	0.18	intergenic
rs12245909	10	34,406,231	A	C	0.80	0.32	0.06	3.5E-07	81.8	0.004	*PARD3*
rs10881824	10	92,468,690	T	C	0.07	-0.46	0.09	3.0E-07	63.5	0.10	intergenic
rs2396457	14	105,417,766	A	G	0.38	-0.35	0.07	3.2E-07	54.3	0.14	*AHNAK2*
**Europeans**											
rs6686805	1	169,512,643	A	C	0.67	-0.04	0.007	3.4E-07	39.2	0.11	*F5*
rs139843727	3	66,316,022	A	C	0.99	0.22	0.04	2.9E-07	0	0.44	*SLC25A26*
rs4968553	17	59,428,962	G	C	0.84	0.06	0.01	4.6E-07	0	0.62	*BCAS3*

EAF: Effect Allele Frequency, Eff: Effect (Alternate) allele, Neff: Non-Effect (Reference) allele

I^2: I-square heterogeneity statistic; P_Q: Cochran's Q statistic's P-value

Supplementary Table 3: Multi-ancestry meta-analysis results of circulating total-tau levels for the lead genetic variants in each locus passing the threshold of $5\times10^{-8}<P<5\times10^{-7}$ in at least one ancestry-specific meta-analysis

		**Random Effects (RE2)**	**Heterogeneity****		**Europeans**		**African Americans**		
**rsid***	**Marker ID**	***P***	**I^2**	**P_Q**	**EAF**	***P***	**EAF**	***P***	**Gene**
rs6686805	1:169512643:A:C	1.1E-06	66.90	0.08	0.67	3.4E-07	0.51	0.27	*F5*
rs10931428	2:153864017:A:T	5.6E-05	96.37	1.5E-07	0.27	0.97	0.05	1.4E-07	intergenic
rs7609578	3:60084971:A:G	2.9E-05	95.93	7.3E-07	0.32	0.31	0.40	2.7E-07	*FHIT*
rs4607127	3:95798669:A:G	3.0E-05	95.97	6.3E-07	0.24	0.30	0.18	2.6E-07	intergenic
rs62363197	5:71440155:A:G	6.6E-05	95.88	8.3E-07	0.07	0.52	0.05	4.3E-07	*MAP1B*
rs11768686	7:20899530:A:G	4.1E-05	96.37	1.5E-07	0.16	0.26	0.04	2.3E-07	intergenic
rs12245909	10:34406231:A:C	7.7E-06	95.76	1.2E-06	0.98	0.23	0.80	3.4E-07	*PARD3*
rs10881824	10:92468690:C:T	2.6E-05	96.37	1.5E-07	0.06	0.23	0.07	3.0E-07	intergenic
rs4968553	17:59428962:C:G	8.2E-07	0.00	0.40	0.16	4.7E-07	0.64	0.96	*BCAS3*

EAF: Effect Allele Frequency

Han and Eskin's Random Effects model (RE2): Random effects model to detect associations under heterogeneity

* rs111836296 on chr4, rs74710969 on chr5, rs78432219 on chr8, rs182648500 and rs76339728 on chr7 are extremely rare in Europeans, rs139843727 on chr3 is monomorphic in African Americans, and thus they were not included in this multi-ancestry meta-analysis

** I^2: I-square heterogeneity statistic; P_Q: Cochran's Q statistic's P-value

Supplementary Table 4: Look-up of known genetic determinants of neurological diseases in the European meta-analysis of circulating total-tau levels

**Chr**	**Build 37 Pos (bp)**	**Eff**	**NEff**	**EAF**	**Beta**	***P***	**I^2**	**P_Q**	**Traits reported for the locus**
17	44,019,712	A	G	0.38	0.20	**8.9E-143**	42.5	0.07	Plasma total-tau, PSP, PD, AD, WMH
1	169,510,524	A	G	0.68	-0.04	**4.2E-07**	37.5	0.12	stroke
19	44,614,208	T	C	0.83	-0.03	7.6E-04	0	0.78	AD
7	50,225,391	A	G	0.47	-0.02	8.9E-04	0	0.54	AD
14	94,920,647	T	C	0.04	0.06	0.001	0	0.93	AD
7	82,377,068	A	T	0.03	-0.07	0.001	30.0	0.20	AD
9	27,630,562	T	C	0.61	-0.03	0.002	0	0.45	AD
12	40,352,996	T	C	0.73	-0.02	0.002	0	0.47	PD
14	105,385,352	A	C	0.14	-0.03	0.002	0	0.58	Plasma total-tau
3	71,981,089	T	C	0.10	-0.04	0.003	17.4	0.29	AD
12	118,299,481	T	C	0.01	0.09	0.003	61.6	0.02	AD

P-values in bold passed the multiple-testing correction threshold (for the number of tests performed at each locus)

EAF: Effect Allele Frequency, Eff: Effect (alternate) allele, Neff: Non-Effect (reference) allele

I^2: I-square heterogeneity statistic; P_Q: Cochran's Q statistic's P-value

AD: Alzheimer’s Disease, PD: Parkinson’s Disease, PSP: progressive supranuclear palsy, WMH: White Matter Hyperintensities

Supplementary Table 5: Association results of the circulating total-tau genetic risk score with incident AD, stroke and four brain MRI traits performed in the Framingham Heart Study (FHS). The genetic risk score was constructed based on the distinct genome-wide associations (rs242557 and rs376284405) in the circulating total-tau European meta-analysis excluding FHS.

**Outcome**	**N**	**Beta**	**SE**	**P**
Plasma total-tau	6,018	0.31	0.01	**3.5E-97**
Incident AD	140 cases / 2775 controls	-0.38	0.25	0.13
Incident Stroke	149 cases / 3461 controls	0.24	0.22	0.28
Hippocampal volume**	4298	-0.004	0.02	0.85
White Matter Hyperintensities**	3489	-0.17	0.22	0.43
Total brain volume**	4310	-1.49	1.99	0.45
Intracranial volume	4310	15.05	4.13	**2.7E-04**
Intracranial volume*	4167	13.84	4.2	**9.8E-04**

*model adjusted for *APOE4*

** model adjusted for intracranial volume

Results in bold passed the multiple-testing correction threshold of P=0.05/6=0.008 (correction for six traits tested).

Logistic or linear mixed-effects models were used, adjusted for age at baseline or at MRI and sex, while accounting for relatedness. For the brain MRI analyses, participants with dementia, stroke, large brain infarcts, tumor or any other finding that could have affected the scan were excluded.

Supplementary Table 6: Two sample Mendelian Randomization with circulating total-tau levels (exposure) and several neurological traits (outcomes), based on large European GWAS summary statistics (IEU GWAS database: https://gwas.mrcieu.ac.uk/)

Outcome	id.outcome	Consortium	nsnp	B	SE	Pval	Qpval	Method
Family history of Alzheimer's Disease	ebi-a-GCST005921	UKBB	12	0.027	0.048	0.592	0.624	MR Egger
	ebi-a-GCST005921		12	0.005	0.026	0.830	0.685	IVW
Alzheimer's Disease	finn-b-G6_ALZHEIMER	FinnGen	14	0.085	0.183	0.651	0.343	MR Egger
	finn-b-G6_ALZHEIMER		14	0.182	0.112	0.104	0.383	IVW
Alzheimer's Disease /Dementia	ukb-b-14043	UKBB	5	0.002	0.001	0.247	0.292	MR Egger
	ukb-b-14043		5	0.0003	0.001	0.786	0.149	IVW
White Matter Hyperintensities		ISGC	11	-0.235	0.299	0.451	0.065	MR Egger
			11	-0.186	0.138	0.177	0.095	IVW
Stroke	finn-b-C_STROKE	FinnGen	14	0.039	0.139	0.782	0.002	MR Egger
	finn-b-C_STROKE		14	0.010	0.084	0.907	0.003	IVW
**Analysis excluding heterogeneous SNP on chr 1, rs12727861**								
Stroke	finn-b-C_STROKE	FinnGen	13	0.037	0.128	0.776	0.012	MR Egger
	finn-b-C_STROKE		13	-0.0002	0.077	0.998	0.017	IVW
Stroke	ukb-b-6358	UKBB	9	-0.002	0.002	0.378	0.597	MR Egger
	ukb-b-6358		9	-0.001	0.001	0.471	0.656	IVW
Vascular/Stroke	ukb-b-8714	UKBB	10	-0.001	0.002	0.695	0.788	MR Egger
	ukb-b-8714		10	0.001	0.001	0.416	0.744	IVW
Stroke	ukb-d-C_STROKE	UKBB	15	0.001	0.003	0.578	0.098	MR Egger
	ukb-d-C_STROKE		15	0.0003	0.001	0.843	0.119	IVW
Parkinson's Disease	ieu-a-812	Other	2	0.268	1.488	0.857	0.096	IVW
Parkinson's Disease	finn-b-G6_PARKINSON	FinnGen	14	-0.008	0.221	0.971	0.401	MR Egger
	finn-b-G6_PARKINSON		14	0.065	0.135	0.632	0.467	IVW
Parkinson's Disease	ukb-b-16943	UKBB	5	0.001	0.001	0.365	0.715	MR Egger
	ukb-b-16943		5	0.001	0.001	0.262	0.810	IVW

nsnp: SNPs used as instruments for the exposure were selected based on the association with circulating total-tau levels at $P \leq 5 \times 10^{-6}$ in the European meta-analysis; Qpval: heterogeneity test p-value; IVW: Inverse Variance Weighted; UKBB: UK Biobank

Supplementary Table 7: Two sample Mendelian Randomization with circulating total-tau levels (outcome) and several neurological traits (exposure), based on large European GWAS summary statistics (IEU GWAS database: https://gwas.mrcieu.ac.uk/)

id.exposure	nsnp	b	se	pval	Qpval	Method
**Alzheimer's Disease**						
ebi-a-GCST005921	24	0.117	0.114	0.315	0.828	MR-Egger
ebi-a-GCST005921	24	0.031	0.031	0.309	0.839	IVW
finn-b-G6_ALZHEIMER	10	0.015	0.010	0.179	0.602	MR-Egger
finn-b-G6_ALZHEIMER	10	0.012	0.008	0.122	0.674	IVW
ukb-b-14043	3	1.217	5.377	0.858	0.829	MR-Egger
ukb-b-14043	3	-0.838	2.868	0.770	0.882	IVW
**Stroke**						
finn-b-C_STROKE	12	-0.009	0.128	0.948	0.931	MR-Egger
finn-b-C_STROKE	12	-0.024	0.035	0.506	0.959	IVW
ukb-b-6358	4	18.895	16.275	0.366	0.235	MR-Egger
ukb-b-6358	4	6.735	3.306	0.042	0.290	IVW
ukb-b-8714	8	6.041	10.629	0.590	0.056	MR-Egger
ukb-b-8714	8	2.676	2.643	0.311	0.086	IVW
ukb-d-C_STROKE	11	-4.304	3.831	0.290	0.219	MR-Egger
ukb-d-C_STROKE	11	1.188	1.896	0.531	0.120	IVW
**Parkinson's Disease (PD)**						
finn-b-G6_PARKINSON	8	0.024	0.048	0.628	0.041	MR Egger
finn-b-G6_PARKINSON	8	0.013	0.020	0.523	0.065	IVW
ieu-a-812	13	0.097	0.145	0.517	**3.17E-32**	MR Egger
ieu-a-812	13	0.039	0.034	0.255	**3.57E-32**	IVW
ukb-b-16943	2	0.118	5.604	0.983	0.677	IVW
**PD (analysis without 1 heterogeneous SNP on chr 17, rs415430)**						
finn-b-G6_PARKINSON	8	0.024	0.048	0.628	0.041	MR Egger
finn-b-G6_PARKINSON	8	0.013	0.020	0.523	0.065	IVW
ieu-a-812	12	-0.033	0.039	0.415	0.368	MR Egger
ieu-a-812	12	0.0003	0.010	0.972	0.385	IVW
ukb-b-16943	2	0.118	5.604	0.983	0.677	IVW
**White Matter Hyperintensities (WMH)**						
WMH	14	0.138	0.118	0.263	**0.009**	MR Egger
WMH	14	-0.026	0.047	0.578	**0.003**	IVW
**WMH (analysis without 1 heterogeneous SNP on chr 17, rs4793173)**						
WMH	13	0.064	0.097	0.523	0.170	MR Egger
WMH	13	0.010	0.036	0.775	0.202	IVW

nsnp: SNPs used as instruments for the exposure were selected based on the top hits ($P \leq 5 \times 10^{-8}$) in large European GWAS summary statistics (IEU GWAS database); Qpval: heterogeneity test p-value; IVW: Inverse Variance Weighted; UKBB: UK Biobank

Supplementary Table 8: Power calculation for the two sample Mendelian Randomization with circulating total-tau levels (exposure) and several neurological traits (outcomes), based on large European GWAS summary statistics (IEU GWAS database: https://gwas.mrcieu.ac.uk/)

					Power			
**Alzheimer's Disease**	**N**	**Ncases**	**Ncontrols**	**ratio**	**OR=1.05**	**OR=1.10**	**OR=1.20**	**OR=1.50**
finn-a-G6_ALZHEIMER	69,524	1,739	67,785	38.979	8.20%	19.80%	56.50%	99.70%
finn-a-AD	69,524	1,788	67,736	37.884	8.30%	20.10%	57.50%	99.80%
finn-a-F5_ALZHDEMENT	95,388	1,051	94,337	89.759	6.50%	13.70%	38.20%	95.80%
ukb-b-14043	361,264	2,094	359,170	171.523	9.20%	23.20%	65.20%	99.90%
ebi-a-GCST005921	314,278	42,034	272,244	6.477	74.90%	99.90%	100%	100%
finn-b-G6_ALZHEIMER	218,792	3,899	214,893	55.115	13.50%	38.60%	89.10%	100%
**Stroke**	**N**	**Ncases**	**Ncontrols**	**ratio**	**OR=1.05**	**OR=1.10**	**OR=1.20**	**OR=1.50**
ukbb-b-16334	361,925	12,031	349,894	29.083	31.90%	82.90%	100%	100%
ukb-b-8714	461,880	7,055	454,825	64.468	20.80%	61%	99%	100%
ukb-b-6358	462,933	6,116	456,817	74.692	18.70%	55.20%	97.90%	100%
ukbb-a-221	260,486	8,481	252,005	29.714	23.70%	68.10%	99.60%	100%
ukb-d-C_STROKE	361,194	6,146	355,048	57.769	18.70%	55.20%	97.90%	100%
finn-a-C_STROKE	82,564	7,144	75,420	10.557	19.40%	57.20%	98.40%	100%
finn-b-C_STROKE	180,862	18,661	162,201	8.692	42.10%	93.00%	100.00%	100%
**Parkinson's Disease**	**N**	**Ncases**	**Ncontrols**	**ratio**	**OR=1.05**	**OR=1.10**	**OR=1.20**	**OR=1.50**
ukb-b-16943	361,199	2,005	359,194	179.149	9%	22.50%	63.40%	99.90%
finn-a-G6_PARKINSON	69,542	953	68,589	71.972	6.20%	12.80%	35.20%	94%
ieu-a-818	1,672	816	856	1.049	4.70%	7.90%	18.20%	65%
ieu-a-812	5,691	1,713	3978	2.322	6.90%	15.20%	43%	97.80%
finn-b-G6_PARKINSON	218,792	2,162	216,630	100.199	9.30%	23.80%	67%	100.00%
**White matter hyperintensities**	**N**				**beta=0.1**	**beta=0.2**		
Traylor 2018	11,226				85%	100%		

Power calculations were conducted using the power analysis calculator at: https://sb452.shinyapps.io/power/, considering a proportion of variance explained by the SNPs on the exposure of 8%.

Supplementary Table 9: Main results from the meta-analysis of rare-variant aggregation tests conducted using whole exome sequence data from FHS and RSI, and missense and loss of function rare variants

	CMC								SKAT		
**Gene**	**p**	**beta**	**se**	**cmafTotal**	**cmafUsed**	**nsnpsTotal**	**nsnpsUsed**	**nmiss**	**p**	**cmaf**	**nmiss**
**MAF≤1%**											
UBASH3B	0.04	-0.239	0.118	0.337	0.008	148	13	14187	**4.7E-07**	0.008	264389
ZFP28	0.03	-0.209	0.096	0.501	0.009	241	17	12505	**2.1E-09**	0.009	420126
LCT	0.10	-0.108	0.065	0.391	0.022	399	37	31847	**2.0E-07**	0.022	673002
REM1	0.03	-0.215	0.096	0.320	0.010	105	21	16323	**6.5E-07**	0.010	158016
ELFN2	0.03	0.173	0.077	0.015	0.015	206	29	20225	**6.9E-07**	0.015	327057
SLIT3	0.09	-0.085	0.050	0.648	0.035	466	62	47203	**7.3E-07**	0.035	745333
MYO1G	**1.2E-06**	-0.279	0.058	0.581	0.022	339	32	21621	4.3E-04	0.022	549768
RUSF1	2.1E-03	-0.317	0.103	0.009	0.009	183	15	12791	**6.7E-10**	0.009	313728
DELE1	0.24	-0.104	0.089	0.426	0.010	176	22	15667	**3.2E-12**	0.010	268972
NSD3	1.7E-05	-0.376	0.088	0.022	0.011	265	18	14414	**8.1E-09**	0.011	443597
**MAF≤5%**											
UBASH3B	0.04	-0.239	0.118	0.337	0.008	148	13	14187	**4.7E-07**	0.008	264389
ZFP28	0.03	-0.209	0.096	0.501	0.009	241	17	12505	**2.1E-09**	0.009	420126
LCT	0.10	-0.108	0.065	0.391	0.022	399	37	31847	**2.0E-07**	0.022	673002
REM1	0.03	-0.215	0.096	0.320	0.010	105	21	16323	**6.5E-07**	0.010	158016
ELFN2	0.03	0.173	0.077	0.015	0.015	206	29	20225	**6.9E-07**	0.015	327057
SLIT3	0.40	-0.030	0.036	0.648	0.065	466	64	48086	0.01	0.065	745333
MYO1G	**1.2E-06**	-0.279	0.058	0.581	0.022	339	32	21621	4.3E-04	0.022	549768
RUSF1	2.1E-03	-0.317	0.103	0.009	0.009	183	15	12791	**6.7E-10**	0.009	313728
DELE1	0.24	-0.104	0.089	0.426	0.010	176	22	15667	**3.2E-12**	0.010	268972
NSD3	0.01	-0.142	0.056	0.022	0.022	265	19	14414	8.4E-03	0.022	443597

cmaf: cumulative MAF

Main results (bold) presented were selected based on a P-value < 0.05/20,000/2 =1.25×10^{-6} (Bonferroni correction for the number of genes on the genome and number of tests performed) & cumulative minor allele count of 30.

Supplementary Table 10: Main results from the meta-analysis of rare-variant aggregation tests conducted using whole exome sequence data from FHS and RSI, and high or moderate impact variants based on Ensembl Variant Effect Predictor annotations

	CMC								SKAT		
**Gene**	**p**	**beta**	**se**	**cmafTotal**	**cmafUsed**	**nsnpsTotal**	**nsnpsUsed**	**nmiss**	**p**	**cmaf**	**nmiss**
**MAF≤1%**											
UBASH3B	0.04	-0.239	0.118	0.337	0.008	156	13	14187	**4.7E-07**	0.008	282621
ZFP28	0.03	-0.209	0.096	0.501	0.009	248	17	12505	**2.1E-09**	0.009	436079
LCT	0.10	-0.107	0.065	0.392	0.022	413	38	32730	**2.3E-07**	0.022	703512
REM1	0.03	-0.200	0.094	0.321	0.011	108	22	17719	**7.5E-07**	0.011	163970
ELFN2	0.03	0.162	0.074	0.016	0.016	214	30	21108	3.7E-06	0.016	338309
SLIT3	0.09	-0.085	0.050	0.648	0.035	480	62	47203	**7.3E-07**	0.035	777239
MYO1G	**1.2E-06**	-0.279	0.058	0.581	0.022	346	32	21621	4.3E-04	0.022	562929
RUSF1	2.1E-03	-0.317	0.103	0.009	0.009	194	15	12791	**6.7E-10**	0.009	336005
DELE1	0.24	-0.104	0.089	0.426	0.010	183	22	15667	**3.2E-12**	0.010	283529
NSD3	1.3E-05	-0.376	0.086	0.023	0.012	279	19	15810	**8.6E-09**	0.012	473224
**MAF≤5%**											
UBASH3B	0.04	-0.239	0.118	0.337	0.008	156	13	14187	**4.7E-07**	0.008	282621
ZFP28	0.03	-0.209	0.096	0.501	0.009	248	17	12505	**2.1E-09**	0.009	436079
LCT	0.10	-0.107	0.065	0.392	0.022	413	38	32730	**2.3E-07**	0.022	703512
REM1	0.03	-0.200	0.094	0.321	0.011	108	22	17719	**7.5E-07**	0.011	163970
ELFN2	0.03	0.162	0.074	0.016	0.016	214	30	21108	3.7E-06	0.016	338309
SLIT3	0.40	-0.030	0.036	0.648	0.065	480	64	48086	0.01	0.065	777239
MYO1G	**1.2E-06**	-0.279	0.058	0.581	0.022	346	32	21621	4.3E-04	0.022	562929
RUSF1	2.1E-03	-0.317	0.103	0.009	0.009	194	15	12791	**6.7E-10**	0.009	336005
DELE1	0.24	-0.104	0.089	0.426	0.010	183	22	15667	**3.2E-12**	0.010	283529
NSD3	9.7E-03	-0.145	0.056	0.023	0.023	279	20	15810	8.5E-03	0.023	473224

cmaf: cumulative MAF

Main results (bold) presented were selected based on a P-value < 0.05/20,000/2 = 1.25×10^{-6} (Bonferroni correction for the number of genes on the genome and number of tests performed) & cumulative minor allele count of 30.

Supplementary Table 11: Neurological traits reported in the GWAS catalog for the main genes identified in the meta-analyses of circulating total-tau levels

GWAS Catalog Reported Trait	PMID
IL15	
Neurofibrillary tangles (SNP x SNP interaction)	32450446
FHIT	
Caudal anterior-cingulate cortex volume	31530798
Cognitive function	25644384
Intracranial aneurysm	29531279
Diffuse plaques (SNP x SNP interaction)	32450446
Neuritic plaques (SNP x SNP interaction)	32450446
Neurofibrillary tangles (SNP x SNP interaction)	32450446
Total PHF-tau (SNP x SNP interaction)	32450446
Neuroticism	30643256
Pars triangularis volume	31530798
ADAMTS12	
Neurofibrillary tangles	31497858
Neurofibrillary tangles (SNP x SNP interaction)	32450446
Total PHF-tau (SNP x SNP interaction)	32450446
PARD3	
General cognitive ability	29844566
F5	
Hippocampal atrophy	22745009
Ischemic stroke	26732560
BCAS3	
Alzheimer's disease (cognitive decline)	23535033
Medial orbital frontal cortex volume	31530798
UBASH3B	
Total PHF-tau (SNP x SNP interaction)	32450446
SLIT3	
Response to antidepressant	30468137
Total PHF-tau (SNP x SNP interaction)	32450446
Neuritic plaques (SNP x SNP interaction)	32450446
Neurofibrillary tangles (SNP x SNP interaction)	32450446
SLC25A26	
Cortical thickness	34560273
Cortical surface area	34560273
Cortical surface area (MOSTest)	32665545
Brain morphology (MOSTest)	32665545

Supplementary Table 12: Look-up of the main hits (P<10^{-5}) from the published ADNI GWAS of circulating tau levels (Chen *et al*., 2017) in the European-ancestry meta-analysis of GWAS of circulating total-tau levels (seven studies excluding ADNI)

**rsid**	**Chr**	**Build 37 Pos (bp)**	**Eff**	**NEff**	**EAF**	**Beta**	**SE**	***P***	**Direction***	**HetISq**	**HetPVal**	**Gene**
rs2187213	6	162,634,337	A	G	0.35	0.0005	0.008	0.95	+++-++++-	0	0.67	*PARK2*
rs7047280	9	23,297,808	T	C	0.60	-0.0009	0.007	0.90	----+-+-+	31.3	0.17	*ELAVL2*
rs7072793	10	6,106,266	T	C	0.59	0.008	0.007	0.22	++++----+	32.1	0.16	*IL2RA*
rs242557	17	44,019,712	A	G	0.36	0.20	0.008	6.4E-136	+++++++++	48.9	0.05	*MAPT*

*Direction of effects for FHS, RSI, RSII, MEMENTO_1, CARDIA, CHS, VETSA, ARIC, MEMENTO_2

EAF: Effect Allele Frequency, Eff: Effect (alternate) allele, Neff: Non-Effect (reference) allele

Supplementary Table 13: Minor Allele Frequency threshold and imputation reference panel used for each study included in the meta-analyses of circulating total-tau levels

	**Reference Panel**	**N**	**Minor Allele Frequency threshold**
**African American studies**			
CARDIA	1000 Genomes	111	0.09
CHS	Haplotype Reference Consortium	273	0.04
ARIC	1000 Genomes	569	0.02
**TOTAL**		953	
**European studies**			
FHS	Haplotype Reference Consortium	6,018	0.002
RSI	Haplotype Reference Consortium	2,169	0.005
RSII	Haplotype Reference Consortium	960	0.01
MEMENTO_1	Haplotype Reference Consortium	336	0.03
MEMENTO_2	Haplotype Reference Consortium	1,738	0.006
CARDIA	Haplotype Reference Consortium	315	0.03
CHS	Haplotype Reference Consortium	1,396	0.007
VETSA	1000 Genomes	754	0.01
ARIC	Haplotype Reference Consortium	549	0.02
ADNI	Genotyping only	486	0.02
**TOTAL**		14,721	

Supplementary Table 14: Minor Allele Frequency threshold and imputation reference panel used for each study included in the APOE4 carriers meta-analyses of circulating total-tau levels

	Reference Panel	N	Minor Allele Frequency threshold
**African American studies**			
CARDIA	1000 Genomes	--	--
CHS	Haplotype Reference Consortium	--	--
ARIC	1000 Genomes	217	0.05
**TOTAL**		217	
**European studies**			
FHS	Haplotype Reference Consortium	1,268	0.008
RSI	Haplotype Reference Consortium	565	0.02
RSII	Haplotype Reference Consortium	252	0.04
MEMENTO_1	Haplotype Reference Consortium	146	0.07
MEMENTO_2	Haplotype Reference Consortium	472	0.02
CARDIA	Haplotype Reference Consortium	--	--
CHS	Haplotype Reference Consortium	337	0.03
VETSA	1000 Genomes	214	0.05
ARIC	Haplotype Reference Consortium	147	0.07
ADNI	Genotyping only	239	0.04
**TOTAL**		3,640	

Supplementary Table 15: Minor Allele Frequency threshold and imputation reference panel used for each study included in the APOE4 non-carriers meta-analyses of circulating total-tau levels

	**Reference Panel**	**N**	**Minor Allele Frequency threshold**
**African American studies**			
CARDIA	1000 Genomes	--	--
CHS	Haplotype Reference Consortium	178	0.06
ARIC	1000 Genomes	344	0.03
**TOTAL**		522	
**European studies**			
FHS	Haplotype Reference Consortium	4,490	0.002
RSI	Haplotype Reference Consortium	1,523	0.007
RSII	Haplotype Reference Consortium	700	0.01
MEMENTO_1	Haplotype Reference Consortium	208	0.04
MEMENTO_2	Haplotype Reference Consortium	1,250	0.008
CARDIA	Haplotype Reference Consortium	224	0.05
CHS	Haplotype Reference Consortium	1,011	0.008
VETSA	1000 Genomes	540	0.02
ARIC	Haplotype Reference Consortium	381	0.03
ADNI	Genotyping only	247	0.04
**TOTAL**		10,574	

Supplementary Notes

Supplementary Note 1

The Alzheimer's Disease Neuroimaging Initiative (ADNI) Study

The ADNI was launched in 2003 as a public-private partnership, led by Principal Investigator Michael W. Weiner, MD. ADNI is a global research study that actively supports the investigation and development of treatments that slow or stop the progression of Alzheimer’s disease. In this multisite longitudinal study, researchers at 63 sites in the US and Canada track the progression of AD in the human brain with clinical, imaging, genetic and biospecimen biomarkers through the process of normal aging, early mild cognitive impairment (EMCI), and late mild cognitive impairment (LMCI) to dementia or AD. The overall goal of ADNI is to validate biomarkers for use in Alzheimer’s disease clinical treatment trials. The primary goal of ADNI has been to test whether serial magnetic resonance imaging (MRI), positron emission tomography (PET), other biological markers, and clinical and neuropsychological assessment can be combined to measure the progression of mild cognitive impairment (MCI) and early Alzheimer's disease (AD). For up-to-date information, see www.adni-info.org.

Data used in preparation of this article were obtained from the Alzheimer's Disease Neuroimaging Initiative (ADNI) database (adni.loni.usc.edu). Phenotypic and genetic data were downloaded from the data repository hosted at the Laboratory of Neuroimaging (LONI) at the University of Southern California, the LONI Image & Data Archive (IDA). Principal Component Analyses (PCA) were performed using Eigensoft based on pruned genetic data, with exclusion of complex and HLA regions. Ethnic outliers were removed based on 6SD from the mean. Plasma tau was analyzed with the Human Total Tau kit (research use only grade, Quanterix, Lexington, MA) on the Simoa HD-1 analyzer (CE marker). ADNI1 SNP genotype data were used to perform the GWAS (Illumina Human610-Quad BeadChip). Only non-Hispanic whites

ADNI1 participants were included in the GWAS of circulating tau levels. Winsorizing at 4 SD was used to removed outliers. QC on genetic data was performed (call-rate > 0.99, P_{HWE} > 10-4; MAF > 1%). Calculation of an empirical kinship matrix was performed to account for relatedness in the association analyses. Linear mixed-effects models were used to evaluate the association of genetic variants with circulating total-tau levels, adjusted for age, sex, and PCs. The total sample size of participants included in the analyses was N=486.

Supplementary Note 2

The Atherosclerosis Risk in Communities Study (ARIC)

The ARIC study is a prospective population-based study of atherosclerosis and clinical atherosclerotic diseases in 15,792 men and women, including 11,478 white participants, drawn from 4 United States communities (Suburban Minneapolis, Minnesota; Washington County, Maryland; Forsyth County, North Carolina; and Jackson, Mississippi).[1] In the first 3 communities, the sample reflects the demographic composition of the community. In Jackson, only black residents were enrolled. Participants were between age 45 and 64 years at their baseline examination in 1987-1989 when blood was drawn for DNA extraction and participants consented to genetic analysis.

Plasma tau was measured on a subset of ARIC participants with brain MRI data (N=1892) on blood samples collected at Visit 3 (1993-1995) using the Simoa Human Neurology 4-Plex A assay and the Quanterix Simoa HD-X analyzer.

Genotyping was performed using Affy6.0 genotyping chip. Imputation was performed using the Michigan Imputation Server v1.0.2 and 1000G p3v5 AFR for African-ancestry (AA) participants and HRC r1.1 2016 for European-ancestry (EA) participants. Phasing was performed using Eagle v2.3. GWAS was performed using Probabel. Association analyses were adjusted for age, sex, center and PCs (PC4 for AA and PCs 1, and 2 for EA).

Supplementary Note 3

The Coronary Artery Risk Development in Young Adults (CARDIA) Study

The CARDIA study is a prospective, multi-center investigation of the natural history and etiology of cardiovascular disease in African Americans and whites 18-30 years of age at the time of initial examination. The initial examination included 5,115 participants selectively recruited to represent proportionate racial, gender, age, and education groups from four communities: Birmingham, AL; Chicago, IL; Minneapolis, MN; and Oakland, CA. Participants from the Birmingham, Chicago, and Minneapolis centers were recruited from the total community or from selected census tracts. Participants from the Oakland center were randomly recruited from the Kaiser-Permanente health plan membership. Details of the study design have been published.[2] From the time of initiation of the study in 1985-1986, eight follow-up examinations have been conducted at years 2, 5, 7, 10, 15, 20, 25, and 30. DNA extraction for genetic studies was performed at the Y10 examination. After considering availability of adequate amounts of high-quality DNA, appropriate informed consent and genotyping quality control and assurance procedures, genotype data were available on 955 African American and 1711 white individuals. Genotyping was performed using Affy6.0 genotyping chip. Imputation was performed using the Michigan Imputation Server v1.0.2 and 1000G p3v5 AFR for African-ancestry (AA) (EA) participants and HRC r1.1 2016 for European-ancestry participants. GWAS was performed using Probabel. Association analyses were adjusted for age, sex, center, and PCs (PC4 for AA and PCs 1, and 2 for EA).

Plasma tau was quantified on a subset of the cohort (N=709) with a brain MRI on blood samples collected at Y25 using the Simoa Human Neurology 4-Plex A assay and the Quanterix Simoa HD-X analyzer.

Supplementary Note 4

The Cardiovascular Health Study (CHS)

The Cardiovascular Health Study (CHS) is a population-based cohort study of risk factors for coronary heart disease and stroke in adults ≥65 years conducted across four field centers.[3] The original predominantly European ancestry cohort of 5,201 persons was recruited in 1989-1990 from random samples of the Medicare eligibility lists; subsequently, an additional predominantly African American cohort of 687 persons was enrolled for a total sample of 5,888. Blood samples were drawn from all participants at their baseline examination and DNA was subsequently extracted from available samples. CHS was approved by institutional review committees at each field center and individuals in the present analysis had available DNA and gave informed consent including consent to use of genetic information for the study of cardiovascular disease. Serum tau was measured in singlet with the Quanterix single molecule array platform at the CHS Central Laboratory at the University of Vermont using the HD-X analyzer and the Simoa Human Neurology 4-Plex A assay. Preliminary studies on ~200 duplicate samples demonstrated very high reproducibility. The detectable range was 0.096 - 325.60 pg/mL. Inter-assay coefficients of variation were 9.20-12.88%.

For this ancillary study, all participants who underwent routine oral glucose tolerance testing at the 1996-1997 clinic visit were included. Entry criteria for the OGTT included in-person attendance in 1996-1997, fasting status, and absence of anti-diabetic medication.

Genotyping was performed at the General Clinical Research Center's Phenotyping/Genotyping Laboratory at Cedars-Sinai among CHS participants who consented to genetic testing and had DNA available using the Illumina 370CNV BeadChip system (for European ancestry participants, in 2007) or the Illumina HumanOmni1-Quad_v1 BeadChip system (for African American participants, in 2010).

All African Americans with available DNA and appropriate consent were genotyped. European ancestry participants with presence at study baseline of coronary heart disease, congestive

heart failure, peripheral vascular disease, valvular heart disease, stroke or transient ischemic attack or lack of available DNA were excluded from the GWAS study sample.

Beyond laboratory genotyping failures, participants were excluded if they had a call rate<=95% or if their genotype was discordant with known sex or prior genotyping (to identify possible sample swaps). After quality control, genotyping was successful for 3,268 European ancestry and 823 African American participants.

In CHS, the following exclusions were applied to identify a final set of 306,655 autosomal SNPs: call rate < 97%, HWE P < 10-5, > 2 duplicate errors or Mendelian inconsistencies (for reference CEPH trios), heterozygote frequency = 0, SNP not found in HapMap.

Imputation to the HRC r1.1 2016 panel was performed on the Michigan imputation server. SNPs were excluded for variance on the allele dosage ≤0.01.

GWAS analyses were performed using R. Tau values were log2-transformed. Linear regression models were adjusted for age at assay, sex, study site, and principal components (PC1-9 for EA, PC1 for AA).

Supplementary Note 5

The Framingham Heart Study (FHS)

The FHS is a prospective, population-based study that has followed participants from the town of Framingham, MA, to understand the determinants of cardiovascular diseases. The population was almost entirely of European descent at the beginning of the study. The first generation (Original cohort/Gen1), followed since 1948, included 5,209 participants; survivors are still invited to participate in examinations every two years.[4] The second generation (Offspring cohort/Gen2), followed since 1971, comprised 5,124 offspring of the original cohort and spouses of these offspring, including 3,514 biological offspring; they have attended examinations every 4 to 8 years.[5] The third-generation (Gen3), enrolled in 2002, included 4,095 children from the largest Offspring cohort families; they have attended three examinations 4 years apart.[6] All

cohorts are under active surveillance for cardiovascular events, stroke, and dementia. All participants provided written informed consent at each examination. This study was approved by the IRB of the Boston University Medical Center.

Tau quantification

Plasma total-tau was measured in 7,096 FHS participants (exam 28 for Gen1 (2004-2005), exam 8 for Gen2 (2005-2008), and exam 2 for Gen3 (2008-2011)). Fasting blood samples obtained at the FHS clinic were centrifuged, aliquoted and stored at -80°C. Plasma total-tau was quantified using two Quanterix instruments (Lexington, MA): the Simoa™ Tau 2.0 Kit and the Simoa HD-1 analyzer that automatically diluted the samples by 4-fold. The assay was based on a molecule digital enzyme-linked immunosorbent assay (ELISA) with a detection limit of 0.019 pg/mL, which can detect both normal and phosphorylated tau isoforms. The analytical range was between 0.06 and 360 pg/mL. The intra- and inter-assay coefficients of variation were 4.1% and 7.5%, respectively. As a quality control (QC) procedure, we included 292 phantom duplicate samples (11.6% of the samples). The QC procedures identified a set of runs with less ideal correspondence between phantoms and original samples from Gen 3. Therefore, tau measurements were categorized into two batches: a first with ideal quality (N=6,468) and a second with less optimal quality (N=628). We did not detect a significant batch effect and thus, we included all individuals in our analysis.

Genome-wide genotyping and imputations

In the 1990s and early 2000s, DNA samples were collected in the three FHS generations for genetic research. All individuals provided consent for genotyping. In 2007, the FHS began genotyping for the NHLBI funded Single Nucleotide Polymorphism (SNP)-Health Association Resource (SHARe) project using approximately 550 000 SNPs (Affymetrix 250K Nsp and 250K Sty mapping arrays plus Affymetrix 50K gene-centered supplemental array) in 9,274 participants from the three generations (including over 1,500 families). Individuals who did not pass QC criteria (call rate < 97%, extreme heterozygosity or high Mendelian error rate) were

excluded. After QC, 8,481 genotyped individuals were available for imputation, 6,018 of whom had information on plasma total-tau. Five non-European participants were excluded based on principal components analysis.

Imputation was performed on the Michigan Imputation Server using miniMACH3 and the Haplotype Reference Consortium (HRC) reference panel release 1.1 April 2016 17 using SNPs passing the following criteria: call-rate ≥ 97%, Hardy-Weinberg P ≥ 10-6, < 1000 Mendelian errors, and minor allele frequency (MAF) ≥ 1%. Prior to imputation, phasing was performed using the duoHMM algorithm incorporated into SHAPEIT2 to account for parental genotypes.

Supplementary Note 6

The MEMENTO Study

Memento prospectively included, from Jan 2011 to June 2014, 2,323 individuals in French memory presenting with either isolated subjective cognitive complaints (SCCs) or mild cognitive impairment (MCI; defined as test performance 1.5 SD below age, sex, and education-level norms) while not demented (Clinical Dementia Rating [CDR] <1).[7] They were followed every 6 months for 5 years. Tau quantification was performed using Simoa™ Tau 2.0 Kit or HD-X from Quanterix (Lexington, MA). Intra CV was 7.1%, and inter CV was 8.6%. Imputation was performed using the Michigan Imputation Server panel with HRC.r1.1.2016 (predominantly European Ancestry), and phasing was performed using Eagle. PCA software was Plink v1.90. Pre-imputation QC included removing SNPs with MAF<0.01, call-rate<0.98 and HWE<0.001; removing samples with call-rate<0.05, heterozygosity beyond 3SD, failed sex-check using genotype data of X-chromosome, related sample based on IBD (pi_hat>0.1875). PCA outliers were defined beyond 6SD of PC1 and PC2. GWAS software was Plink v1.90. Covariates in the association analyses were age, sex, center, and PCs 1-4.

Supplementary Note 7

The Rotterdam Study (RS1 and RS2)

The Rotterdam Study is a population-based cohort study among inhabitants of a district of Rotterdam (Ommoord), the Netherlands, that aims to examine the determinants of disease and health in the elderly with a focus on neurogeriatric, cardiovascular, bone, and eye disease.[8] In 1990-1993, 7,983 persons aged ≥ 55 years participated and were re-examined every 3 to 4 years (Rotterdam Study I). In 2000-2001, the cohort was expanded by 3,011 persons who were of the same age but had not yet been part of the Rotterdam Study (Rotterdam Study II) and recently moved into the area. All participants had blood collected during their first center visit, which was followed by DNA extraction. Genotyping was done in participants with high-quality extracted DNA in 2007-2008 and was performed at the Human Genotyping Facility, Genetic Laboratory Department of Internal Medicine, Erasmus MC, Rotterdam, The Netherlands. Imputation of SNPs was established using the Michigan Imputation server and the HRC reference panel. More specifically, the SHAPEIT2 software was used (v2.r790) to phase the data and Minimac 3 was employed for imputation to the HRC reference panel (v1.0). QC included deletion of participants with a genotype completion rate (<90%), a low genotype call rate (<95%), sex-mismatches, duplicate pairs (just one participant), uncalled variants in over 5% of the individuals and significant violations of the expected Hardy–Weinberg Equilibrium proportions (P<10−6).

Tau quantification was performed at Quanterix (Lexington, MA, USA) in two batches, using a single molecule array and the (Simoa) HD-1 analyzer platform. The Simoa Human Neurology 3-Plex A assay was used to measure total plasma tau. Samples were tested twice and two quality control (QC) samples were run for total tau assessment. Details on assay performance have been published previously.[9] Data was excluded from analyses when duplicates were present, if single measurements were not available, if the concentration coefficient of variation surpassed

20% or if control samples were not within range. The GWAS software used was rvtest. Covariates in the association analyses were age, sex, and PCs (1-5).

Supplementary Note 8

The Vietnam Era Twin Study of Aging (VETSA)

VETSA is a longitudinal behavior genetics study of cognitive and brain aging.[10-12] There are three key features to the VETSA design. First, the sample has a narrow age range (~10 years), allowing for examination of individual differences in aging trajectories. Second, the initial assessment was in midlife (mean age 56; range 51-60), which provides a baseline for the transition to older age. Third, data previously collected on VETSA participants is also available; of particular importance is a test of general cognitive ability administered at average age 20 and repeated in each wave of the study.

Participants are members of the Vietnam Era Twin Registry, which is housed at the VA Puget Sound Health Care System in Seattle, WA, USA. All of the twins served in some branch of US military service at some time during the Vietnam era (1965-1975). A 1992 study sought to recruit all Registry twins. It enrolled approximately 8000 individuals, including approximately 3300 pairs. VETSA participants were randomly recruited from those 3300 pairs. Eligibility for inclusion was based only on being 51-59 years old at the time of recruitment and willingness of both twins in a pair to participate. Both members of a pair did not need to participate to be included in wave 2 or wave 3. The average interval between waves was approximately 6 years. Additional participants, including, attrition replacement participants, were included at waves 2 and 3. Subsets have multi-modal MRI and neuroendocrine data.

Data collection includes questionnaires filled out at home plus a daylong series of assessments. These include cognitive/neuropsychological assessment of multiple cognitive domains, personality and psychosocial assessments, and health/medical assessments.

There are approximately 55% MZ and 45% DZ twins in the sample. For cognitive, psychosocial, and health/medical data, there are 1291 individuals at wave 1, 1207 at wave 2 data, and 1196 at wave 3. Brain MRIs were obtained from 546 individuals at wave 1, 452 at wave 2, and 525 at wave 3. At wave 1 only, salivary cortisol, testosterone, and DHEAS data were collected on 780 participants.

VETSA participants live throughout the US. The sample is primarily Caucasian (European-American): 86% based on self-report. Only those of European-American ancestry based on SNP data were included in GWAS analyses. The average educational attainment is 13.8 years (SD=2.1). At wave 1, 79% were married, and 78% were employed full-time. Nearly 80% report no combat experience. The sample is similar with respect to health and lifestyle characteristics to American men in their age range based on US Center for Disease Control and Prevention data.

Tau Quantification

Tau high throughput bioassays platforms or single analyte assays using the Quanterix Simoa HD-X or Fujirebio were used in this study. These human-specific immunoassays have been documented for measurements of these components in human plasma in multiple publications from multiple labs including Dr. Robert Rissman at UC San Diego.[13-16] These assays are used routinely in the Rissman lab for clinical trials and all assays were performed according to the manufacturer instructions following strict standard operating procedures for sample handling. All reagents were purchased in bulk to avoid batch effects and all were completed by technicians who were blind to sample characteristics.

Genome-wide Genotyping

Genotyping, QC, and imputation have been described in detail elsewhere.[17] In brief, individuals were genotyped with the Illumina HumanOmniExpress-24 v1.0A beadchip and imputation was performed with 1000 genomes Phase 3 Reference data using MiniMac on the Michigan Imputation Server (https://imputationserver.sph.umich.edu).

The analysis was done with RMW-RareMetalWorker v 4.13.7. Covariates included age, PCs 1-3, -- RMW already incorporates adjustments which take twinness into account including MZ/DZ relationships.

Supplementary Note 9

Rotterdam Study exome sequencing description

In this project, exome-sequencing analysis was performed in a subset of RS-I participants (N=883) who had plasma total-Tau levels. In the RS study, whole exomes of randomly selected subset of 2,628 individuals from the RS-I population were sequenced at the Human Genomics facility of the Department of Internal Medicine, Erasmus Medical Centre, The Netherlands.[18] Sequencing was performed at an average depth of 54×. Whole blood genomic DNA was processed using the Illumina TruSeq DNA Library preparation (Illumina, Inc., San Diego, CA, USA), followed by exome capture using the Nimblegen SeqCap EZ V2 capture kit (Roche Nimblegen, Inc., Madison, WI, USA). Paired-end 2 × 100 bp sequencing was performed at six samples per lane on an Illumina HiSeq2000 sequencer (Illumina, San Diego, CA, USA) using the Illumina TruSeq V3 protocol.[19]

The sequence reads were aligned to human genome build 19 using Burrows–Wheeler Aligner.[20] Subsequently, the aligned reads were processed further using Picard's MarkDuplicates, SAMtools[21] and the Indel Realignment and Base Quality Score Recalibration tools from Genome Analysis Toolkit.[22] Genetic variants were called using the HaplotypeCaller from Genome Analysis Toolkit. Samples with low concordance to genotyping array (< 95%), or that differed 4 standard deviations from the mean on either the number of detected variants per sample, transition to transversion ratio or high heterozygote to homozygote ratio and low call rate (<90%) were removed from the data. Single nucleotide variants (SNVs) with a low call rate (< 90%) and out of Hardy–Weinberg equilibrium (P-value $<10^{-8}$) were also removed from the data.

Supplementary Note 10

Framingham Heart Study exome sequencing description as part of the Cohorts for Heart and Aging Research in Genomic Epidemiology (CHARGE) Consortium

In this project, exome-sequencing analysis was performed in a subset of FHS participants (N=1,396) who had plasma total-Tau levels. Whole exome sequencing of FHS samples in Freeze 5 (N=1,702 FHS samples sequenced) was completed as part of a collaborative sequencing effort by the CHARGE Consortium.

The exome was captured using NimbleGen SeqCap EZ VCRome (Roche, Basel, Switzerland). The enriched library was then sequenced in paired-end mode using a single lane by Illumina HiSeq platform (HiSeq 2000 or HiSeq 2500) at Human Genome Sequencing Center at Baylor College of Medicine. The Mercury pipeline[23] was used to process sequencing data. The reads were mapped to the human genome reference sequence (NCBI Genome Build 37, 2009) using Burrows-Wheeler Aligner.[20, 21] Single nucleotide variants (SNVs) and indels were called using the Atlas2 suite.[24, 25] The mean read depth was 92x, and more than 92% of target regions were covered by at least 20 unique reads. The mean depth of coverage among FHS samples was 84-fold for targeted regions.

Rigorous quality control was performed to exclude low-quality variants or samples and has been described in detail previously. Briefly, all SNV calls were filtered on the following: low SNV posterior probability (<0.95), low variant read count (<3), variant read ratio <0.25 or >0.75, strand-bias of more than 99% variant reads in a single strand direction, and total coverage less than 10-fold. Variants were also excluded if they were outside exon capture regions, monomorphic, had a missing rate higher than 20%, a mappability score less than 0.8, and a mean depth of coverage higher than 500. Variants not meeting Hardy-Weinberg equilibrium ($p<5\times10^{-6}$) in ancestry-specific groups were excluded. Samples were excluded if they had missingness higher than 20%, or if they fell more than 6 standard deviations (SD) in the FHS

samples for mean depth, number of singletons, heterozygote to homozygote ratio, or transition to transversion ratio.

Supplementary Note 11

Study-specific Acknowledgements

ADNI

Data used in preparation of this article were obtained from the Alzheimer's Disease Neuroimaging Initiative (ADNI) database (adni.loni.usc.edu). As such, the investigators within the ADNI contributed to the design and implementation of ADNI and/or provided data but did not participate in analysis or writing of this report. A complete listing of ADNI investigators can be found at:

http://adni.loni.usc.edu/wp-content/uploads/how_to_apply/ADNI_Acknowledgement_List.pdf

Data collection and sharing for this project was funded by the Alzheimer's Disease Neuroimaging Initiative (ADNI) (National Institutes of Health Grant U01 AG024904) and DOD ADNI (Department of Defense award number W81XWH-12-2-0012). ADNI is funded by the National Institute on Aging, the National Institute of Biomedical Imaging and Bioengineering, and through generous contributions from the following: AbbVie, Alzheimer's Association; Alzheimer's Drug Discovery Foundation; Araclon Biotech; BioClinica, Inc.; Biogen; Bristol-Myers Squibb Company; CereSpir, Inc.; Cogstate; Eisai Inc.; Elan Pharmaceuticals, Inc.; Eli Lilly and Company; EuroImmun; F. Hoffmann-La Roche Ltd and its affiliated company Genentech, Inc.; Fujirebio; GE Healthcare; IXICO Ltd.;Janssen Alzheimer Immunotherapy Research & Development, LLC.; Johnson & Johnson Pharmaceutical Research & Development LLC.; Lumosity; Lundbeck; Merck & Co., Inc.;Meso Scale Diagnostics, LLC.; NeuroRx Research; Neurotrack Technologies; Novartis Pharmaceuticals Corporation; Pfizer Inc.; Piramal Imaging; Servier; Takeda Pharmaceutical Company; and Transition Therapeutics. The Canadian Institutes of Health Research is providing funds to support ADNI clinical sites in Canada. Private

sector contributions are facilitated by the Foundation for the National Institutes of Health (www.fnih.org). The grantee organization is the Northern California Institute for Research and Education, and the study is coordinated by the Alzheimer's Therapeutic Research Institute at the University of Southern California. ADNI data are disseminated by the Laboratory for Neuro Imaging at the University of Southern California.

ARIC

The Atherosclerosis Risk in Communities study has been funded in whole or in part with Federal funds from the National Heart, Lung, and Blood Institute, National Institutes of Health, Department of Health and Human Services (contract numbers HHSN268201700001I, HHSN268201700002I,
HHSN268201700003I, HHSN268201700004I and HHSN268201700005I), R01HL087641, R01HL059367 and R01HL086694; National Human Genome Research Institute contract U01HG004402; and National Institutes of Health contract HHSN268200625226C. The authors thank the staff and participants of the ARIC study for their important contributions. Infrastructure was partly supported by Grant Number UL1RR025005, a component of the National Institutes of Health and NIH Roadmap for Medical Research. Support for this GWAS analysis was provided by grants UH3-NS100605 and U01-AG052409.

CARDIA

The Coronary Artery Risk Development in Young Adults Study (CARDIA) is conducted and supported by the National Heart, Lung, and Blood Institute (NHLBI) in collaboration with the University of Alabama at Birmingham (HHSN268201800005I & HHSN268201800007I), Northwestern University (HHSN268201800003I), University of Minnesota (HHSN268201800006I), and Kaiser Foundation Research Institute (HHSN268201800004I). CARDIA was also partially supported by the Intramural Research Program of the National Institute on Aging (NIA) and an intra-agency agreement between NIA and NHLBI (AG0005). Genotyping was funded as part of the NHLBI Candidate-gene Association Resource (N01-HC-

65226) and the NHGRI Gene Environment Association Studies (GENEVA) (U01-HG004729, U01-HG04424, and U01-HG004446). Support for this GWAS analysis was provided by grants UH3-NS100605 and U01-AG052409. This manuscript has been reviewed and approved by CARDIA for scientific content.

CHARGE

The CHARGE cohorts are supported in part by the National Heart, Lung, and Blood Institute (NHLBI) infrastructure grants R01HL105756 (Psaty), RC2HL102419 (Boerwinkle) and the neurology working group is supported by the National Institute on Aging (NIA) R01 grant AG033193.

CHS

Cardiovascular Health Study (CHS): This CHS research was supported by NHLBI contracts HHSN268201200036C, HSN268200800007C, HHSN268201800001C, N01HC55222, N01HC85079, N01HC85080, N01HC85081, N01HC85082, N01HC85083, N01HC85086, 75N92021D00006 and NHLBI grants U01HL080295, R01HL087652, R01HL105756, R01HL103612, R01HL120393, and U01HL130114 with additional contribution from the National Institute of Neurological Disorders and Stroke (NINDS). Additional support was provided through K24AG065525, R01AG053325, R01AG023629 and R01AG033193 from the National Institute on Aging (NIA). A full list of principal CHS investigators and institutions can be found at CHS-NHLBI.org.

The provision of genotyping data was supported in part by the National Center for Advancing Translational Sciences, CTSI grant UL1TR001881, and the National Institute of Diabetes and Digestive and Kidney Disease Diabetes Research Center (DRC) grant DK063491 to the Southern California Diabetes Endocrinology Research Center.

The content is solely the responsibility of the authors and does not necessarily represent the official views of the National Institutes of Health.

FHS

We thank the Framingham Heart Study (FHS) participants, as well as the study team (especially the investigators and staff of the neurology team) for their contributions and dedication to the study. The authors are pleased to acknowledge that the computational work reported on in this paper was performed on the Shared Computing Cluster, which is administered by Boston University Research Computing Services. URL: www.bu.edu/tech/support/research/.

This work was supported by the National Heart, Lung and Blood Institute's Framingham Heart Study (Contract No. N01-HC-25195, No. HHSN268201500001I and No. 75N92019D00031). This study was also supported by grants from the National Institute of Aging (R01s AG054076, AG049607, RF1 AG059421, U01 AG049505, U01 AG052409, and U01 AG058589), the National Institute of Neurological Disorders and Stroke (R01 NS017950), the National Heart, Lung and Blood Institute (UH3 NS100605, R01 HL093029, U01 HL096917). Dr. DeCarli is supported by the UCD Alzheimer's Disease Center (P30 AG 010129). Dr. Pase is supported by a Heart Foundation Future Leader Fellowship (GNT102052). Dr. Sarnowski is supported by the NIA K99AG066849. The funders had no role in study design, data collection, analysis, decision to publish, or preparation of the manuscript.

MEMENTO

The MEMENTO cohort was sponsored by the Fondation Plan Alzheimer (Alzheimer Plan 2008–2012). This work was also supported by the following: CIC 1401-EC, Bordeaux University Hospital, Inserm, the University of Bordeaux, and a grant (European Alzheimer & Dementia BioBank, EADB) from the EU Joint Program – Neurodegenerative Disease Research (JPND). S Debette is supported by a grant overseen by the French National Research Agency (ANR) as part of the "Investment for the Future" Programme ANR-18-RHUS-002, the EU JPND, the ERC and the EU H2020 under grant agreements No 643417, 640643, 667375, and 754517. Part of the computations were performed at the Bordeaux Bioinformatics Center (CBiB), University of Bordeaux and at the CREDIM (Centre de Ressource et Développement en Informatique

Médicale) at University of Bordeaux, on a server infrastructure supported by the Fondation Claude Pompidou. Inserm UMR1167 is also funded by Inserm, Institut Pasteur de Lille, the Lille Métropole Communauté Urbaine, and the French government's LABEX DISTALZ program (development of innovative strategies for a transdisciplinary approach to Alzheimer's disease).
VETSA
The content is the responsibility of the authors and does not necessarily represent official views of the NIA, NIH, or VA. The U.S. Department of Veterans Affairs, Department of Defense; National Personnel Records Center, National Archives and Records Administration; National Opinion Research Center; National Research Council, National Academy of Sciences; and the Institute for Survey Research, Temple University provided invaluable assistance in the creation of the VET Registry. The Cooperative Studies Program of the U.S. Department of Veterans Affairs provided financial support for development and maintenance of the Vietnam Era Twin Registry. We would also like to acknowledge the continued cooperation and participation of the members of the VET Registry and their families.
This work was supported by U.S. National Institute on Aging grants R01AG050595, R01AG022381, R01AG03798, R01AG062483, and P01AG055367.
RS
The Rotterdam Study is funded by Erasmus Medical Center and Erasmus University, Rotterdam, Netherlands Organization for the Health Research and Development (ZonMw), the Research Institute for Diseases in the Elderly (RIDE), the Ministry of Education, Culture and Science, the Ministry for Health, Welfare and Sports, the European Commission (DG XII), and the Municipality of Rotterdam. This Study is further supported by NWO (Vici 918.76.619). The authors are grateful to the study participants, the staff from the Rotterdam Study and the participating general practitioners and pharmacists. The generation and management of genome-wide association study genotype data for the Rotterdam Study is supported by the Netherlands Organisation of Scientific Research NWO Investments (nr. 175.010.2005.011, 911-

03-012). This study is funded by the Research Institute for Diseases in the Elderly (014-93-015; RIDE2), the Netherlands Genomics Initiative (NGI)/Netherlands Organisation for Scientific Research (NWO) project nr. 050-060-810. The work of CMvD is supported by the NGI Center of Medical Systems Biology. We thank Pascal Arp, Mila Jhamai, Marijn Verkerk, Lizbeth Herrera and Marjolein Peters for their help in creating the GWAS database, and Karol Estrada and Maksim V. Struchalin for their support in creation and analysis of imputed data. The plasma concentrations of total-tau were assessed through the Janssen Prevention Center in Leiden, the Netherlands, on anonymized plasma samples without knowledge of disease status. Janssen had no role in study design and data collection.

Supplementary Note 12

ADNI Group Acknowledgements

Michael Weiner, MD (UC San Francisco, Principal Investigator, Executive Committee); Paul Aisen, MD (UC San Diego, ADCS PI and Director of Coordinating Center Clinical Core, Executive Committee, Clinical Core Leaders); Ronald Petersen, MD, PhD (Mayo Clinic, Rochester, Executive Committee, Clinical Core Leader); Clifford R. Jack, Jr., MD (Mayo Clinic, Rochester, Executive Committee, MRI Core Leader); William Jagust, MD (UC Berkeley, Executive Committee; PET Core Leader); John Q. Trojanowki, MD, PhD (U Pennsylvania, Executive Committee, Biomarkers Core Leader); Arthur W. Toga, PhD (USC, Executive Committee, Informatics Core Leader); Laurel Beckett, PhD (UC Davis, Executive Committee, Biostatistics Core Leader); Robert C. Green, MD, MPH (Brigham and Women's Hospital, Harvard Medical School, Executive Committee and Chair of Data and Publication Committee); Andrew J. Saykin, PsyD (Indiana University, Executive Committee, Genetics Core Leader); John Morris, MD (Washington University St. Louis, Executive Committee, Neuropathology Core Leader); Leslie M. Shaw (University of Pennsylvania, Executive

Committee, Biomarkers Core Leader); Enchi Liu, PhD (Janssen Alzheimer Immunotherapy, ADNI 2 Private Partner Scientific Board Chair); Tom Montine, MD, PhD (University of Washington) ; Ronald G. Thomas, PhD (UC San Diego); Michael Donohue, PhD (UC San Diego); Sarah Walter, MSc (UC San Diego); Devon Gessert (UC San Diego); Tamie Sather, MS (UC San Diego,); Gus Jiminez, MBS (UC San Diego); Danielle Harvey, PhD (UC Davis;); Michael Donohue, PhD (UC San Diego); Matthew Bernstein, PhD (Mayo Clinic, Rochester); Nick Fox, MD (University of London); Paul Thompson, PhD (USC School of Medicine); Norbert Schuff, PhD (UCSF MRI); Charles DeCArli, MD (UC Davis); Bret Borowski, RT (Mayo Clinic); Jeff Gunter, PhD (Mayo Clinic); Matt Senjem, MS (Mayo Clinic); Prashanthi Vemuri, PhD (Mayo Clinic); David Jones, MD (Mayo Clinic); Kejal Kantarci (Mayo Clinic); Chad Ward (Mayo Clinic); Robert A. Koeppe, PhD (University of Michigan, PET Core Leader); Norm Foster, MD (University of Utah); Eric M. Reiman, MD (Banner Alzheimer's Institute); Kewei Chen, PhD (Banner Alzheimer's Institute); Chet Mathis, MD (University of Pittsburgh); Susan Landau, PhD (UC Berkeley); Nigel J. Cairns, PhD, MRCPath (Washington University St. Louis); Erin Householder (Washington University St. Louis); Lisa Taylor Reinwald, BA, HTL (Washington University St. Louis); Virginia Lee, PhD, MBA (UPenn School of Medicine); Magdalena Korecka, PhD (UPenn School of Medicine); Michal Figurski, PhD (UPenn School of Medicine); Karen Crawford (USC); Scott Neu, PhD (USC); Tatiana M. Foroud, PhD (Indiana University); Steven Potkin, MD UC (UC Irvine); Li Shen, PhD (Indiana University); Faber Kelley, MS, CCRC (Indiana University); Sungeun Kim, PhD (Indiana University); Kwangsik Nho, PhD (Indiana University); Zaven Kachaturian, PhD (Khachaturian, Radebaugh & Associates, Inc and Alzheimer's Association's Ronald and Nancy Reagan's Research Institute); Richard Frank, MD, PhD (General Electric); Peter J. Snyder, PhD (Brown University); Susan Molchan, PhD (National Institute on Aging/ National Institutes of Health); Jeffrey Kaye, MD (Oregon Health and Science University); Joseph Quinn, MD (Oregon Health and Science University); Betty Lind, BS (Oregon Health and Science University);

Raina Carter, BA (Oregon Health and Science University); Sara Dolen, BS (Oregon Health and Science University); Lon S. Schneider, MD (University of Southern California); Sonia Pawluczyk, MD (University of Southern California); Mauricio Beccera, BS (University of Southern California); Liberty Teodoro, RN (University of Southern California); Bryan M. Spann, DO, PhD (University of Southern California); James Brewer, MD, PhD (University of California San Diego); Helen Vanderswag, RN (University of California San Diego); Adam Fleisher, MD (University of California San Diego); Judith L. Heidebrink, MD, MS (University of Michigan); Joanne L. Lord, LPN, BA, CCRC (University of Michigan); Ronald Petersen, MD, PhD (Mayo Clinic, Rochester); Sara S. Mason, RN (Mayo Clinic, Rochester); Colleen S. Albers, RN (Mayo Clinic, Rochester); David Knopman, MD (Mayo Clinic, Rochester); Kris Johnson, RN (Mayo Clinic, Rochester); Rachelle S. Doody, MD, PhD (Baylor College of Medicine); Javier Villanueva Meyer, MD (Baylor College of Medicine); Munir Chowdhury, MBBS, MS (Baylor College of Medicine); Susan Rountree, MD (Baylor College of Medicine); Mimi Dang, MD (Baylor College of Medicine); Yaakov Stern, PhD (Columbia University Medical Center); Lawrence S. Honig, MD, PhD (Columbia University Medical Center); Karen L. Bell, MD (Columbia University Medical Center); Beau Ances, MD (Washington University, St. Louis); John C. Morris, MD (Washington University, St. Louis); Maria Carroll, RN, MSN (Washington University, St. Louis); Sue Leon, RN, MSN (Washington University, St. Louis); Erin Householder, MS, CCRP (Washington University, St. Louis); Mark A. Mintun, MD (Washington University, St. Louis); Stacy Schneider, APRN, BC, GNP (Washington University, St. Louis); Angela Oliver, RN, BSN, MSG ; Daniel Marson, JD, PhD (University of Alabama Birmingham); Randall Griffith, PhD, ABPP (University of Alabama Birmingham); David Clark, MD (University of Alabama Birmingham); David Geldmacher, MD (University of Alabama Birmingham); John Brockington, MD (University of Alabama Birmingham); Erik Roberson, MD (University of Alabama Birmingham); Hillel Grossman, MD (Mount Sinai School of Medicine); Effie Mitsis, PhD (Mount Sinai School of Medicine); Leyla deToledo-

Morrell, PhD (Rush University Medical Center); Raj C. Shah, MD (Rush University Medical Center); Ranjan Duara, MD (Wien Center); Daniel Varon, MD (Wien Center); Maria T. Greig, HP (Wien Center); Peggy Roberts, CNA (Wien Center); Marilyn Albert, PhD (Johns Hopkins University); Chiadi Onyike, MD (Johns Hopkins University); Daniel D'Agostino II, BS (Johns Hopkins University); Stephanie Kielb, BS (Johns Hopkins University); James E. Galvin, MD, MPH (New York University); Dana M. Pogorelec (New York University); Brittany Cerbone (New York University); Christina A. Michel (New York University); Henry Rusinek, PhD (New York University); Mony J de Leon, EdD (New York University); Lidia Glodzik, MD, PhD (New York University); Susan De Santi, PhD (New York University); P. Murali Doraiswamy, MD (Duke University Medical Center); Jeffrey R. Petrella, MD (Duke University Medical Center); Terence Z. Wong, MD (Duke University Medical Center); Steven E. Arnold, MD (University of Pennsylvania); Jason H. Karlawish, MD (University of Pennsylvania); David Wolk, MD (University of Pennsylvania); Charles D. Smith, MD (University of Kentucky); Greg Jicha, MD (University of Kentucky); Peter Hardy, PhD (University of Kentucky); Partha Sinha, PhD (University of Kentucky); Elizabeth Oates, MD (University of Kentucky); Gary Conrad, MD (University of Kentucky); Oscar L. Lopez, MD (University of Pittsburgh); MaryAnn Oakley, MA (University of Pittsburgh); Donna M. Simpson, CRNP, MPH (University of Pittsburgh); Anton P. Porsteinsson, MD (University of Rochester Medical Center); Bonnie S. Goldstein, MS, NP (University of Rochester Medical Center); Kim Martin, RN (University of Rochester Medical Center); Kelly M. Makino, BS (University of Rochester Medical Center); M. Saleem Ismail, MD (University of Rochester Medical Center); Connie Brand, RN (University of Rochester Medical Center); Ruth A. Mulnard, DNSc, RN, FAAN (University of California, Irvine); Gaby Thai, MD (University of California, Irvine); Catherine Mc Adams Ortiz, MSN, RN, A/GNP (University of California, Irvine); Kyle Womack, MD (University of Texas Southwestern Medical School); Dana Mathews, MD, PhD (University of Texas Southwestern Medical School); Mary Quiceno, MD (University of Texas Southwestern Medical School); Ramon Diaz

Arrastia, MD, PhD (University of Texas Southwestern Medical School); Richard King, MD (University of Texas Southwestern Medical School); Myron Weiner, MD (University of Texas Southwestern Medical School); Kristen Martin Cook, MA (University of Texas Southwestern Medical School); Michael DeVous, PhD (University of Texas Southwestern Medical School); Allan I. Levey, MD, PhD (Emory University); James J. Lah, MD, PhD (Emory University); Janet S. Cellar, DNP, PMHCNS BC (Emory University); Jeffrey M. Burns, MD (University of Kansas, Medical Center); Heather S. Anderson, MD (University of Kansas, Medical Center); Russell H. Swerdlow, MD (University of Kansas, Medical Center); Liana Apostolova, MD (University of California, Los Angeles); Kathleen Tingus, PhD (University of California, Los Angeles); Ellen Woo, PhD (University of California, Los Angeles); Daniel H.S. Silverman, MD, PhD (University of California, Los Angeles); Po H. Lu, PsyD (University of California, Los Angeles); George Bartzokis, MD (University of California, Los Angeles); Neill R Graff Radford, MBBCH, FRCP (London) (Mayo Clinic, Jacksonville); Francine Parfitt, MSH, CCRC (Mayo Clinic, Jacksonville); Tracy Kendall, BA, CCRP (Mayo Clinic, Jacksonville); Heather Johnson, MLS, CCRP (Mayo Clinic, Jacksonville); Martin R. Farlow, MD (Indiana University); Ann Marie Hake, MD (Indiana University); Brandy R. Matthews, MD (Indiana University); Scott Herring, RN, CCRC (Indiana University); Cynthia Hunt, BS, CCRP (Indiana University); Christopher H. van Dyck, MD (Yale University School of Medicine); Richard E. Carson, PhD (Yale University School of Medicine); Martha G. MacAvoy, PhD (Yale University School of Medicine); Howard Chertkow, MD (McGill Univ., Montreal Jewish General Hospital); Howard Bergman, MD (McGill Univ., Montreal Jewish General Hospital); Chris Hosein, Med (McGill Univ., Montreal Jewish General Hospital); Sandra Black, MD, FRCPC (Sunnybrook Health Sciences, Ontario); Dr Bojana Stefanovic (Sunnybrook Health Sciences, Ontario); Curtis Caldwell, PhD (Sunnybrook Health Sciences, Ontario); Ging Yuek Robin Hsiung, MD, MHSc, FRCPC (U.B.C. Clinic for AD & Related Disorders); Howard Feldman, MD, FRCPC (U.B.C. Clinic for AD & Related Disorders); Benita Mudge, BS (U.B.C. Clinic for AD & Related

Disorders); Michele Assaly, MA Past (U.B.C. Clinic for AD & Related Disorders); Andrew Kertesz, MD (Cognitive Neurology St. Joseph's, Ontario); John Rogers, MD (Cognitive Neurology St. Joseph's, Ontario); Dick Trost, PhD (Cognitive Neurology St. Joseph's, Ontario); Charles Bernick, MD (Cleveland Clinic Lou Ruvo Center for Brain Health); Donna Munic, PhD (Cleveland Clinic Lou Ruvo Center for Brain Health); Diana Kerwin, MD (Northwestern University); Marek Marsel Mesulam, MD (Northwestern University); Kristine Lipowski, BA (Northwestern University); Chuang Kuo Wu, MD, PhD (Northwestern University); Nancy Johnson, PhD (Northwestern University); Carl Sadowsky, MD (Premiere Research Inst (Palm Beach Neurology)); Walter Martinez, MD (Premiere Research Inst (Palm Beach Neurology)); Teresa Villena, MD (Premiere Research Inst (Palm Beach Neurology)); Raymond Scott Turner, MD, PhD (Georgetown University Medical Center); Kathleen Johnson, NP (Georgetown University Medical Center); Brigid Reynolds, NP (Georgetown University Medical Center); Reisa A. Sperling, MD (Brigham and Women's Hospital); Keith A. Johnson, MD (Brigham and Women's Hospital); Gad Marshall, MD (Brigham and Women's Hospital); Meghan Frey (Brigham and Women's Hospital); Jerome Yesavage, MD (Stanford University); Joy L. Taylor, PhD (Stanford University); Barton Lane, MD (Stanford University); Allyson Rosen, PhD (Stanford University); Jared Tinklenberg, MD (Stanford University); Marwan N. Sabbagh, MD (Banner Sun Health Research Institute); Christine M. Belden, PsyD (Banner Sun Health Research Institute); Sandra A. Jacobson, MD (Banner Sun Health Research Institute); Sherye A. Sirrel, MS (Banner Sun Health Research Institute); Neil Kowall, MD (Boston University); Ronald Killiany, PhD (Boston University); Andrew E. Budson, MD (Boston University); Alexander Norbash, MD (Boston University); Patricia Lynn Johnson, BA (Boston University); Thomas O. Obisesan, MD, MPH (Howard University); Saba Wolday, MSc (Howard University); Joanne Allard, PhD (Howard University); Alan Lerner, MD (Case Western Reserve University); Paula Ogrocki, PhD (Case Western Reserve University); Leon Hudson, MPH (Case Western Reserve University); Evan Fletcher, PhD (University of

California, Davis Sacramento); Owen Carmichael, PhD (University of California, Davis Sacramento); John Olichney, MD (University of California, Davis Sacramento); Charles DeCarli, MD (University of California, Davis Sacramento); Smita Kittur, MD (Neurological Care of CNY); Michael Borrie, MB ChB (Parkwood Hospital); T Y Lee, PhD (Parkwood Hospital); Dr Rob Bartha, PhD (Parkwood Hospital); Sterling Johnson, PhD (University of Wisconsin); Sanjay Asthana, MD (University of Wisconsin); Cynthia M. Carlsson, MD (University of Wisconsin); Steven G. Potkin, MD (University of California, Irvine BIC); Adrian Preda, MD (University of California, Irvine BIC); Dana Nguyen, PhD (University of California, Irvine BIC); Pierre Tariot, MD (Banner Alzheimer’s Institute); Adam Fleisher, MD (Banner Alzheimer’s Institute); Stephanie Reeder, BA (Banner Alzheimer’s Institute); Vernice Bates, MD (Dent Neurologic Institute); Horacio Capote, MD (Dent Neurologic Institute); Michelle Rainka, PharmD, CCRP (Dent Neurologic Institute); Douglas W. Scharre, MD (Ohio State University); Maria Kataki, MD, PhD (Ohio State University); Anahita Adeli, MD (Ohio State University); Earl A. Zimmerman, MD (Albany Medical College); Dzintra Celmins, MD (Albany Medical College); Alice D. Brown, FNP (Albany Medical College); Godfrey D. Pearlson, MD (Hartford Hosp, Olin Neuropsychiatry Research Center); Karen Blank, MD (Hartford Hosp, Olin Neuropsychiatry Research Center); Karen Anderson, RN (Hartford Hosp, Olin Neuropsychiatry Research Center); Robert B. Santulli, MD (Dartmouth Hitchcock Medical Center); Tamar J. Kitzmiller (Dartmouth Hitchcock Medical Center); Eben S. Schwartz, PhD (Dartmouth Hitchcock Medical Center); Kaycee M. Sink, MD, MAS (Wake Forest University Health Sciences); Jeff D. Williamson, MD, MHS (Wake Forest University Health Sciences); Pradeep Garg, PhD (Wake Forest University Health Sciences); Franklin Watkins, MD (Wake Forest University Health Sciences); Brian R. Ott, MD (Rhode Island Hospital); Henry Querfurth, MD (Rhode Island Hospital); Geoffrey Tremont, PhD (Rhode Island Hospital); Stephen Salloway, MD, MS (Butler Hospital); Paul Malloy, PhD (Butler Hospital); Stephen Correia, PhD (Butler Hospital); Howard J. Rosen, MD (UC San Francisco); Bruce L. Miller,

MD (UC San Francisco); Jacobo Mintzer, MD, MBA (Medical University South Carolina); Kenneth Spicer, MD, PhD (Medical University South Carolina); David Bachman, MD (Medical University South Carolina); Elizabether Finger, MD (St. Josephs Health Care); Stephen Pasternak, MD (St. Josephs Health Care); Irina Rachinsky, MD (St. Josephs Health Care); John Rogers, MD (St. Josephs Health Care); Andrew Kertesz, MD (St. Josephs Health Care); Dick Drost, MD (St. Josephs Health Care); Nunzio Pomara, MD (Nathan Kline Institute); Raymundo Hernando, MD (Nathan Kline Institute); Antero Sarrael, MD (Nathan Kline Institute); Susan K. Schultz, MD (University of Iowa College of Medicine, Iowa City); Laura L. Boles Ponto, PhD (University of Iowa College of Medicine, Iowa City); Hyungsub Shim, MD (University of Iowa College of Medicine, Iowa City); Karen Elizabeth Smith, RN (University of Iowa College of Medicine, Iowa City); Norman Relkin, MD, PhD (Cornell University); Gloria Chaing, MD (Cornell University); Lisa Raudin, PhD (Cornell University); Amanda Smith, MD (University of South Florida: USF Health Byrd Alzheimer's Institute); Kristin Fargher, MD (University of South Florida: USF Health Byrd Alzheimer's Institute); Balebail Ashok Raj, MD (University of South Florida: USF Health Byrd Alzheimer's Institute)

Supplementary References

1. The Atherosclerosis Risk in Communities (ARIC) Study: design and objectives. The ARIC investigators. *Am. J. Epidemiol.* **129**, 687-702 (1989).

2. Friedman, G. D. *et al*. CARDIA: study design, recruitment, and some characteristics of the examined subjects. *J. Clin. Epidemiol.* **41**, 1105-1116 (1988).

3. Fried, L. P. *et al*. The Cardiovascular Health Study: design and rationale. *Ann. Epidemiol.* **1**, 263-276 (1991).

4. Dawber, T. R. & Kannel, W. B. The Framingham study. An epidemiological approach to coronary heart disease. *Circulation* **34**, 553-555 (1966).

5. Feinleib, M., Kannel, W. B., Garrison, R. J., McNamara, P. M. & Castelli, W. P. The Framingham Offspring Study. Design and preliminary data. *Prev. Med.* **4**, 518-525 (1975).

6. Splansky, G. L. *et al*. The Third Generation Cohort of the National Heart, Lung, and Blood Institute's Framingham Heart Study: design, recruitment, and initial examination. *Am. J. Epidemiol.* **165**, 1328-1335 (2007).

7. Dufouil, C. *et al*. Cognitive and imaging markers in non-demented subjects attending a memory clinic: study design and baseline findings of the MEMENTO cohort. *Alzheimers Res. Ther.* **9**, 67-017-0288-0 (2017).

8. Ikram, M. A. *et al*. Objectives, design and main findings until 2020 from the Rotterdam Study. *Eur. J. Epidemiol.* **35**, 483-517 (2020).

9. de Wolf, F. *et al*. Plasma tau, neurofilament light chain and amyloid-beta levels and risk of dementia; a population-based cohort study. *Brain* **143**, 1220-1232 (2020).

10. Kremen, W. S. *et al*. Genes, environment, and time: the Vietnam Era Twin Study of Aging (VETSA). *Twin Res. Hum. Genet.* **9**, 1009-1022 (2006).

11. Kremen, W. S., Franz, C. E. & Lyons, M. J. VETSA: the Vietnam Era Twin Study of Aging. *Twin Res. Hum. Genet.* **16**, 399-402 (2013).

12. Kremen, W. S., Franz, C. E. & Lyons, M. J. Current Status of the Vietnam Era Twin Study of Aging (VETSA). *Twin Res. Hum. Genet.* **22**, 783-787 (2019).

13. Winston, C. N. *et al*. Prediction of conversion from mild cognitive impairment to dementia with neuronally derived blood exosome protein profile. *Alzheimers Dement. (Amst)* **3**, 63-72 (2016).

14. O'Bryant, S. E. *et al*. A blood screening tool for detecting mild cognitive impairment and Alzheimer's disease among community-dwelling Mexican Americans and non-Hispanic Whites:

A method for increasing representation of diverse populations in clinical research. *Alzheimers Dement.* (2021).
15. O'Bryant, S. E. *et al*. A Precision Medicine Approach to Treating Alzheimer's Disease Using Rosiglitazone Therapy: A Biomarker Analysis of the REFLECT Trials. *J. Alzheimers Dis.* **81**, 557-568 (2021).
16. Kellar, D. *et al*. Intranasal Insulin Reduces White Matter Hyperintensity Progression in Association with Improvements in Cognition and CSF Biomarker Profiles in Mild Cognitive Impairment and Alzheimer's Disease. *J. Prev. Alzheimers Dis.* **8**, 240-248 (2021).
17. Logue, M. W. *et al*. Use of an Alzheimer's disease polygenic risk score to identify mild cognitive impairment in adults in their 50s. *Mol. Psychiatry* **24**, 421-430 (2019).
18. van Rooij, J. G. J. *et al*. Population-specific genetic variation in large sequencing data sets: why more data is still better. *Eur. J. Hum. Genet.* **25**, 1173-1175 (2017).
19. Amin, N. *et al*. Exome-sequencing in a large population-based study reveals a rare Asn396Ser variant in the LIPG gene associated with depressive symptoms. *Mol. Psychiatry* **22**, 634 (2017).
20. Li, H. & Durbin, R. Fast and accurate short read alignment with Burrows-Wheeler transform. *Bioinformatics* **25**, 1754-1760 (2009).
21. Li, H. *et al*. The Sequence Alignment/Map format and SAMtools. *Bioinformatics* **25**, 2078-2079 (2009).
22. McKenna, A. *et al*. The Genome Analysis Toolkit: a MapReduce framework for analyzing next-generation DNA sequencing data. *Genome Res.* **20**, 1297-1303 (2010).
23. Reid, J. G. *et al*. Launching genomics into the cloud: deployment of Mercury, a next generation sequence analysis pipeline. *BMC Bioinformatics* **15**, 30-2105-15-30 (2014).
24. Challis, D. *et al*. An integrative variant analysis suite for whole exome next-generation sequencing data. *BMC Bioinformatics* **13**, 8-2105-13-8 (2012).
25. Danecek, P. *et al*. The variant call format and VCFtools. *Bioinformatics* **27**, 2156-2158 (2011).
