# Supplementary material for: Meta-analysis of genome-wide association studies identifies ancestry-specific associations underlying circulating total tau levels
Source: Commun Biol. 2022 Apr 8;5:336. doi: 10.1038/s42003-022-03287-y (PMC8993877; doi:10.1038/s42003-022-03287-y)
Supplement: Supplementary file 2 — NR Reporting Summary [file 42003_2022_3287_MOESM2_ESM.pdf]

nature portfolio

Corresponding author(s): Chloé Sarnowski

Last updated by author(s): Mar 2, 2022

Reporting Summary

Nature Portfolio wishes to improve the reproducibility of the work that we publish. This form provides structure for consistency and transparency in reporting. For further information on Nature Portfolio policies, see our Editorial Policies and the Editorial Policy Checklist.

Statistics

For all statistical analyses, confirm that the following items are present in the figure legend, table legend, main text, or Methods section.

n/a	Confirmed	
☐	☒	The exact sample size (*n*) for each experimental group/condition, given as a discrete number and unit of measurement
☐	☒	A statement on whether measurements were taken from distinct samples or whether the same sample was measured repeatedly
☐	☒	The statistical test(s) used AND whether they are one- or two-sided<br>*Only common tests should be described solely by name; describe more complex techniques in the Methods section.*
☐	☒	A description of all covariates tested
☐	☒	A description of any assumptions or corrections, such as tests of normality and adjustment for multiple comparisons
☐	☒	A full description of the statistical parameters including central tendency (e.g. means) or other basic estimates (e.g. regression coefficient) AND variation (e.g. standard deviation) or associated estimates of uncertainty (e.g. confidence intervals)
☐	☒	For null hypothesis testing, the test statistic (e.g. *F*, *t*, *r*) with confidence intervals, effect sizes, degrees of freedom and *P* value noted<br>*Give P values as exact values whenever suitable.*
☒	☐	For Bayesian analysis, information on the choice of priors and Markov chain Monte Carlo settings
☒	☐	For hierarchical and complex designs, identification of the appropriate level for tests and full reporting of outcomes
☐	☒	Estimates of effect sizes (e.g. Cohen's *d*, Pearson's *r*), indicating how they were calculated

Our web collection on statistics for biologists contains articles on many of the points above.

Software and code

Policy information about availability of computer code

Data collection	No software was used as only summary statistics from each study were collected.
Data analysis	METAL, Metasoft, Locuszoom, TwosampleMR, FUMA, SeqMeta

For manuscripts utilizing custom algorithms or software that are central to the research but not yet described in published literature, software must be made available to editors and reviewers. We strongly encourage code deposition in a community repository (e.g. GitHub). See the Nature Portfolio guidelines for submitting code & software for further information.

Data

Policy information about availability of data

All manuscripts must include a data availability statement. This statement should provide the following information, where applicable:

- Accession codes, unique identifiers, or web links for publicly available datasets
- A description of any restrictions on data availability
- For clinical datasets or third party data, please ensure that the statement adheres to our policy

All data supporting the findings of this study are available either within the article or the supplementary information. Summary statistics from the ancestry-specific meta-analyses of circulating levels of total-tau have been deposited and are publicly accessible on GWAS catalog FTP (GCST90095001-GCST90096000/GCST90095138/ & GCST90095001-GCST90096000/GCST90095139/). Genome-wide summary statistics for complex disorders used in the secondary analyses were downloaded from public repositories (GWAS catalog:https://www.ebi.ac.uk/gwas/downloads/summary-statistics; and IEU GWAS database: https://gwas.mrcieu.ac.uk/).

Field-specific reporting

Please select the one below that is the best fit for your research. If you are not sure, read the appropriate sections before making your selection.

☒ Life sciences ☐ Behavioural & social sciences ☐ Ecological, evolutionary & environmental sciences

For a reference copy of the document with all sections, see nature.com/documents/nr-reporting-summary-flat.pdf

Life sciences study design

All studies must disclose on these points even when the disclosure is negative.

Sample size	Sample size was achieved as described in the Methods section.
Data exclusions	Exclusion criteria were pre-established and include subjects who withdrawn from the studies, samples or SNPs that fail quality control, samples with missing or extreme phenotypic values, and ethnic outliers based on principal components analyses (participants that do not cluster with Europeans or African-Americans)
Replication	No replication analysis has been performed in this manuscript. However, GWAS results from eight cohorts were meta-analyzed to achieve a total sample size of > 15,000 participants, representing two population groups. Results presented were consistent across studies as shown on Forest plots. The availability of studies with circulating total-tau levels and genetic data is limited.
Randomization	Not relevant as study was observational
Blinding	Not relevant as study was observational

Reporting for specific materials, systems and methods

We require information from authors about some types of materials, experimental systems and methods used in many studies. Here, indicate whether each material, system or method listed is relevant to your study. If you are not sure if a list item applies to your research, read the appropriate section before selecting a response.

Materials & experimental systems

n/a	Involved in the study	
☒	☐	Antibodies
☒	☐	Eukaryotic cell lines
☒	☐	Palaeontology and archaeology
☒	☐	Animals and other organisms
☐	☒	Human research participants
☒	☐	Clinical data
☒	☐	Dual use research of concern

Methods

n/a	Involved in the study	
☒	☐	ChIP-seq
☒	☐	Flow cytometry
☒	☐	MRI-based neuroimaging

Human research participants

Policy information about studies involving human research participants

Population characteristics	We included in our meta-analyses 15,674 participants from eight studies representing two major ancestries: Europeans (N=14,721) and African Americans (N=953). A description of each study is included in the Supplementary Notes 1 to 8. Proportion of males varied from 35% (CHS African Americans) to 100% (VETSA). Mean age ranged from 49 years (3.5) for CARDIA African Americans to 78 years (4.3) for CHS African Americans.
Recruitment	We have analyzed GWAS results from eight studies (7 cohorts from the CHARGE consortium and ADNI). Details about recruitment of participants for each study is available in the Supplementary Notes 1 to 8.
Ethics oversight	All participants provided written informed consent for genetic testing and analyses.

Note that full information on the approval of the study protocol must also be provided in the manuscript.
